# Proximal proteomics analysis reveals DNA polymerase δ subunit 3 is a new MCM2 binding partner and promotes parental histones inheritance in mammalian cells

**DOI:** 10.1038/s41418-025-01619-z

**Published:** 2025-11-27

**Authors:** Yaping Sun, Xiaoyan Liang, Fang Liu, Wenjuan Zhao, Jiaqi Zhou, Yue Li, Yuan Yao, Ziwei Zhang, Gang Li, Kuiming Chan, Daoqin Zhang, Zhiquan Wang, Yuan Gao, Chuanhe Yu, Yuchun Wu, Xing Kang, Lingyu Qiu, Nan Li, Haiyun Gan

**Affiliations:** 1 Shenzhen Key Laboratory of Synthetic Genomics, Guangdong Provincial Key Laboratory of Synthetic Genomics, State Key Laboratory of Quantitative Synthetic Biology, Shenzhen Institute of Synthetic Biology, Shenzhen Institutes of Advanced Technology, Chinese Academy of Sciences, Shenzhen, China; 2 Research Center of Molecular Diagnostics and Sequencing, Research Institute of Tsinghua University in Shenzhen, Shenzhen, China; 3 Shenzhen Synthetic Biology Infrastructure, Shenzhen, China; 4 Shenzhen State Key Laboratory of Genome Manipulation and Biosynthesis, State Key Laboratory of Quantitative Synthetic Biology, Shenzhen Institute of Synthetic Biology, Shenzhen Institutes of Advanced Technology, Chinese Academy of Sciences, Shenzhen, Guangdong China; 5 School of Pharmacy, Faculty of Medicine, Macau University of Science and Technology, Macau, China; 6 Institute for Biological Electron Microscopy, Key Laboratory of Molecular Design for Plant Cell Factory of Guangdong Higher Education Institutes, Department of Chemical Biology & Department of Biology, School of Life Sciences, Southern University of Science and Technology, Shenzhen, China; 7 Centre of Reproduction, Development and Aging, Institute of Translational Medicine, Faculty of Health Sciences, University of Macau, Macau, China; 8 Department of Biomedical Sciences, City University of Hong Kong, Hong Kong, China; 9 Division of Critical Care Medicine, Department of Pediatrics, Stanford University School of Medicine, Stanford, CA USA; 10 Division of Hematology, Department of Medicine, Mayo Clinic, Rochester, MN USA; 11 Department of Pharmacology, Case Western Reserve University, School of Medicine, Cleveland, OH USA; 12 Hormel Institute, University of Minnesota, Austin, MN USA

**Keywords:** Epigenetics, Epigenetics

## Abstract

In mammalian cells, MCM2 and POLE3/4 safeguard the symmetrical segregation of parental histones to the leading and lagging strands of newly synthesized DNA. However, the identity of additional proteins involved in parental histone distribution remains elusive. We used TurboID proximity labeling to identify interaction partners of MCM2 and POLE3/4 in mouse cells. This approach provided a candidate protein library potentially involved in the MCM2 and POLE3/POLE4-mediated process of parental histone segregation. DNA polymerase δ subunit 3 (POLD3) was a protein whose intensity differed between the interactomes of wild-type MCM2 and its histone-binding mutant. We showed POLD3 bound to both MCM2 and the histone (H3-H4)_2_ tetramers. Moreover, MCM2’s histone binding affected interactions between POLD3 and histone H3. More importantly, POLD3 was required for the symmetrical transfer of parental histones H3-H4 to the leading and lagging strands of newly synthesized DNA in mouse cells. In short, our findings establish that POLD3 forms a protein complex with MCM2 and histone (H3-H4)_2_ tetramers, functioning as a novel histone chaperone to regulate parental histone segregation in mammalian cells.

## Introduction

During DNA replication, the recycling of post-translationally modified parental histones is a pivotal step in preserving epigenetic memory [1]. Recent studies have revealed that parental histones in eukaryotic cells are segregated to the leading and lagging strands of the DNA replication fork in a nearly symmetrical manner. In mammalian cells, there is a slight preference for the leading strand, while in yeast, the preference is observed for the lagging strand [2, 3]. Understanding the molecular mechanisms responsible for this process is important because impaired parental histone inheritance disturbs cell fate and promotes tumor progression [4, 5]. Research in yeast and mammalian cells has identified several histone chaperones that play an important role in the transfer of parental and new histones to newly synthesized DNA. Minichromosome maintenance complex component 2 (MCM2), a component of the MCM2-6 replicative complex, and DNA polymerase alpha 1(POLA1), a primase subunit of lagging strand polymerase alpha complex, that are essential for initiating DNA replication, are members of these histone chaperones in mammalian and yeast cells [2, 3, 6, 7]. In addition, Dpb3 and Dpb4, two subunits of the leading-strand DNA polymerase ε in budding yeast cells and the homologs of the mammalian proteins POLE3 and POLE4, have also been identified as members of these histone chaperones [7–9]. In yeast, the MCM2- Chromosome transmission fidelity protein 4 (Ctf4) -DNA polymerase α (Polα) axis is a well-characterized pathway facilitating the transfer of parental histones to lagging strands during DNA replication [3]. However, conducting similar signaling pathway analysis in mammalian cells presents some challenges. These include the longer timelines required for gene editing and the complexity of signaling pathways, which slow the identification of additional histone chaperones. In mammalian cells, the mechanisms by which MCM2 regulates histone segregation remain poorly understood. The role of WD repeat and HMG-box DNA-binding protein 1 (WDHD1)—the human and mouse homolog of *Saccharomyces cerevesiae* Ctf4 [10]—in parental histone distribution remains to be elucidated. To address this gap, we set out to determine the identity of additional chaperone molecules involved in parental histone segregation during DNA replication in mammalian cells.

We hypothesized that proteins interacting with MCM2, POLE3, and POLE4 play functional roles in the signaling pathways involved in parental histone segregation. To investigate this, we employed mass spectrometry (MS)-based proximity labeling (PL) methods, which offer comprehensive identification of protein-protein interaction networks [11]. Unlike traditional co-immunoprecipitation (co-IP) approaches, PL techniques such as BioID, miniTurbo, and TurboID allow the detection of both transient and strong protein-protein interactions in living cells, with an estimated short-range labeling radius of 10–20 nm [12, 13]. Among different PL methods, TurboID offers significantly higher efficiency, enabling 10-minute labeling in cells with lesser-toxic and more easily deliverable biotin than other PL methods. Using TurboID proximity labeling with MCM2, POLE3, and POLE4 as bait proteins, we successfully identified their interactive partners in mammalian cells. Subsequent biochemical analysis revealed that polymerase δ subunit 3 (POLD3) formed a protein complex with MCM2 and histone (H3-H4)_2_ tetramers, acting as a novel histone chaperone to regulate parental histone segregation in mammalian cells. Interestingly, WDHD1, despite being a homolog of yeast Ctf4, did not disrupt the symmetric distribution of parental histones in mammalian cells. These findings offer valuable insights into the mechanisms governing parental histone inheritance in eukaryotic cells.

## Results

### In mouse cells, the MCM2 and POLE3/POLE4 interactomes, as measured with TurboID proximity labeling, are consistent with known biological functions of MCM2, POLE3, and POLE4

#### Proteomic profiling of MCM2 and POLE3/POLE4 interactomes

**Fig. 1:**
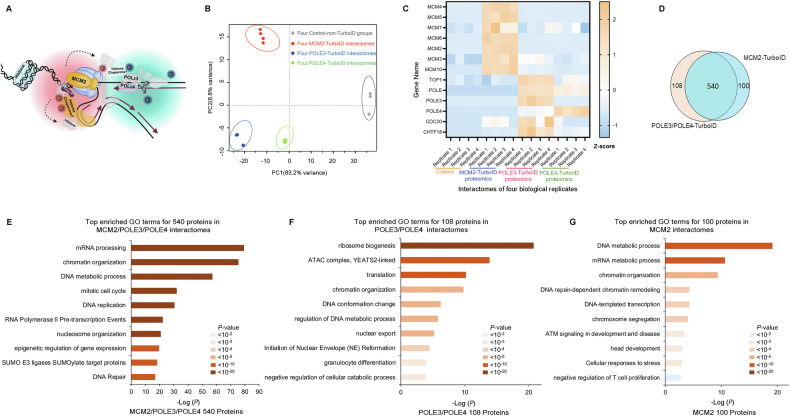
In mouse cells, the MCM2 and POLE3/POLE4 interactomes, as measured with TurboID proximity labeling, are consistent with known biological functions of MCM2, POLE3, and POLE4. **A** A model of MCM2-, POLE3- and POLE4-TurboID proximity labeling in a mouse embryonic fibroblast cell line (NIH3T3); The B letter in the chart represents biotin; Arrows represent the transfer of histones. **B** Principal component analysis (PCA) showed statistical discrimination among the TurboID proximity labeling replicates for MCM2, POLE3 and POLE4. **C** Heat map illustrating specific enrichment of proteins close to MCM2, POLE3 and POLE4 at the replication fork. The X-axis represented interactomes of four biological replicates, Y-axis referred to gene names. **D** Venn diagram of proteins enriched in the TurboID proximity labeling data for POLE3/POLE4 and MCM2 showed the overlap of these interactomes. **E**–**G** GO-term analysis of proteins enriched in the (**E**) MCM2/POLE3/POLE4 interactomes, (**F**) POLE3/POLE4 interactomes, and (**G**) MCM2 interactome.

To systematically investigate the interactomes of MCM2, POLE3, and POLE4 during histone transfer, we fused these bait proteins with the TurboID protein labelling enzyme and overexpressed them in a mouse embryonic fibroblast cell line (NIH3T3 cells; Fig. 1A and Supplementary Fig. 1). To minimize non-specific background labeling during TurboID experiments, we replaced standard fetal bovine serum (FBS), which contains biotin, with dialyzed FBS devoid of biotin in the cell culture medium. This approach reduces the availability of exogenous biotin, ensuring that labeling is specific to the intended targets (Supplementary Fig. 2A, B). Biotinylated proteins were affinity-purified using streptavidin beads and subjected to trypsin digestion and LC-MS/MS with four replicates (Supplementary Fig. 1 and Supplementary Fig. 3).

We hypothesized that TurboID proximity labeling could identify proteins closely associated with MCM2 or POLE3/POLE4 during DNA replication. These proteins would represent potential candidates involved in the process of parental histone distribution. Principal component analysis demonstrated clear clustering of TurboID proximity labeling data replicates for each bait protein (Fig. 1B). In the interactomes of MCM2, POLE3, and POLE4, enrichment parameters for identifying associated proteins were defined as follows: (1) a fold-change greater than 2 compared to control (MCM2/Control >2, POLE3/Control >2, or POLE4/Control >2); (2) detection in at least two of four replicates; (3) *P*-value < 0.05. POLE3 and POLE4 are known to form a protein complex to interact with (H3-H4)_2_ tetramer and regulate parental histone distribution [7, 9]. Therefore, we integrated the POLE3 and POLE4 interactomes, considering proteins enriched in both as potential histone chaperone candidates. Using this approach, the MCM2 interactome identified 640 enriched proteins, while the combined POLE3/POLE4 interactome identified 648 enriched proteins associated with their respective bait proteins during DNA replication (Supplementary Fig. 4).

#### Bioinformatic analysis validated the biological relevance of our interactome datasets and bait proteins

Notably, MCM2 exists in both soluble and chromatin-bound forms within cells, with both forms capable of binding histones H3 and H4 [14]. Thus, we do not specifically exclude the single molecule form or the Cdc45-MCM2–7-Go Ichi Ni San (CMG) complex form of MCM2 in proximity labeling proteomics analysis. In the MCM2 interactome, known MCM2 binding partners, such as MCM2-7 and MCM10, were enriched solely, consistent with MCM2’s function in initiating DNA synthesis [15, 16] (Fig. 1C). Proteins primarily associated with the leading strand, such as CHTF18 and CDC20, were enriched in the POLE3 and POLE4 interactomes, consistent with the function of POLE3 and POLE4 in forming leading strand polymerases [17] (Fig. 1C). Substantial overlap (540 proteins) existed between the MCM2 and POLE3/POLE4 interactomes, with only 100 proteins solely occurring in the MCM2 interactome and only 108 solely occurring in the POLE3/POLE4 interactome (Fig. 1D). Gene Ontology (GO) enrichment analysis revealed the proteins shared between the interactomes were associated with mRNA processing, chromatin organization, mitotic cell cycle, and DNA replication (Fig. 1E). This finding aligns with the established roles of MCM2, POLE3 and POLE4 on parental histone segregation [3, 9] and RNA processing, such as transcription [18, 19]. Among proteins specific to the POLE3/POLE4 interactome, those involved in the acetyltransferase complex ATAC were enriched (Fig. 1F) [20, 21]. This finding was consistent with a report that POLE4, in concert with POLE3, is a component of the ATAC complex [22]. Among proteins specific to the MCM2 interactome, those involved in ataxia-telangiectasia mutated (ATM) protein signaling in development and disease were enriched (Fig. 1G), consistent with a previous report showing MCM2 is a direct substrate of ATM protein kinase [23]. Together, these findings validate the biological relevance of our interactome datasets, demonstrating that the identified proteins are functionally linked to MCM2 or POLE3/POLE4 during DNA replication.

### In mouse cells, network analysis of histone chaperones around protein MCM2 and proteins POLE3/POLE4 during DNA replication provide a candidate protein library joining in parental histone segregation

#### Construction of histone chaperone interactomes around protein MCM2 and proteins POLE3/POLE4

Histone chaperones play an important role in parental and new histone deposition during DNA replication. To systematically identify proteins potentially involved in parental histone transfer, we constructed protein-protein interaction (PPI) networks based on the MCM2 and POLE3/POLE4 TurboID proteomics datasets using the STRING database (v12.0, https://string-db.org/), which integrates experimental, computational, and co-expression evidence to infer functional associations [24]. In this analysis, we used known histone chaperones identified in each dataset as seed nodes to construct candidate interaction networks. For the MCM2-TurboID dataset, the seed chaperones included SSRP1, ATAD2, NASP, RPA1, CHAF1A, CHAF1B, BRD4, DNAJC9, NPM1, and POLA1. For the POLE3/POLE4-TurboID dataset, these were SSRP1, DNAJC9, ATAD2, NASP, RPA1, CHAF1A, CHAF1B, NAP1L1, NAP1L4, BRD4, and NPM1. We then retrieved their first-shell interactors to generate respective interactomes enriched for factors that may contribute to histone recycling.

**Fig. 2:**
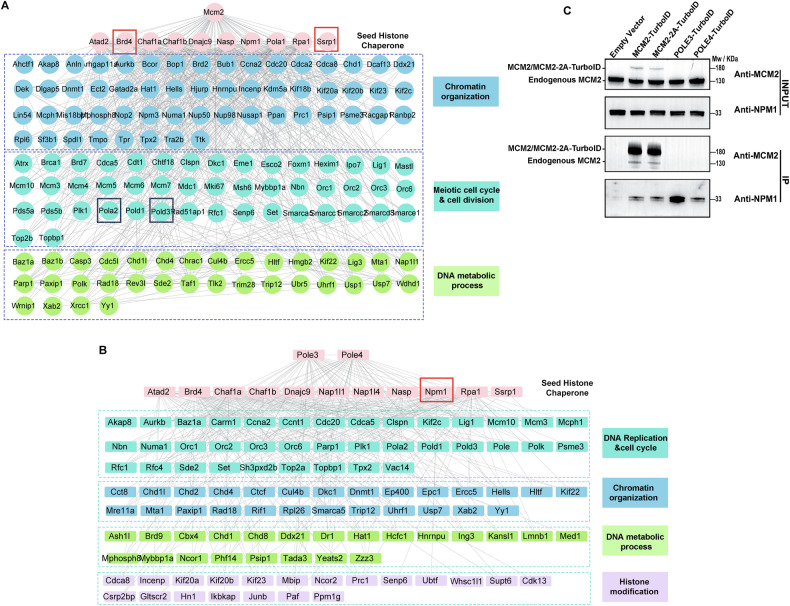
Network analyses of histone chaperones enriched in MCM2 and POLE3/POLE4 interactomes identify candidate proteins involved in parental histone segregation. **A**, **B** Protein–protein interaction (PPI) networks were constructed from MCM2-TurboID (**A**) and POLE3/POLE4-TurboID (**B**) proximity labeling proteomic datasets, using the STRING database (v12.0). Known histone chaperones identified in each dataset were used as seed nodes to extract directly interacting proteins (first-shell interactors). Proteins were included if they met all the following criteria: (1) MCM2/Control > 2, POLE3/Control > 2, or POLE4/Control > 2; (2) proteins quantified at least twice across four replicates; (3) *P*-value < 0.05. The POLE3 and POLE4 interactomes were combined, and proteins common to both were considered as histone chaperone candidates in the POLE3/POLE4 interactomes. Proteins associated with RNA-related functions were excluded from this analysis. Node colors indicated functional classification: Pink: known histone chaperones in the corresponding dataset (e.g., SSRP1, BRD4, CHAF1A); Light blue: chromatin organization; Green cyan: cell cycle & cell division or DNA replication & cell cycle; Green: DNA metabolic process; Purple: histone modification. Red box: validated interactors (BRD4, SSRP1, and NPM1); Dark blue box: DNA polymerase subunits (e.g., POLD3, POLA2). **C** Immunoprecipitation blot of TurboID proximity labeling data showed similar levels of the wild-type MCM2-TurboID and histone-binding mutant MCM2-2A-TurboID fusion proteins in NIH3T3 cells, but higher levels of NPM1 expression for the POLE3 vs MCM2 interactome.

The MCM2-centered interactome comprised 189 proteins directly connected to these seed chaperones, most functioning in DNA metabolic process, cell cycle, chromatin organization and DNA replication (Fig. 2A and Supplementary Fig. 5A). In the POLE3/POLE4 interactome, 146 proteins were identified, closely enriched for functions in the DNA metabolic process, chromosome remodeling, and cell cycle (Fig. 2B and Supplementary Fig. 5B).

#### Analysis of histone chaperone interactomes around protein MCM2 and proteins POLE3/POLE4

Several of the seed chaperones have been previously implicated in histone dynamics during replication. For instance, the chromatin assembly factor-1 (CAF-1) complex, consisting of CHAF1A, CHAF1B, and RBBP4, has been shown to deposit new H3-H4 dimers at the replication fork [25, 26]. Similarly, ATAD2 has been reported to facilitate histone deposition on DNA, contributing to chromatin structure formation [27]. The histone chaperone RPA aids in the assembly of H3-H4 onto DNA to form nucleosomes [28].

However, there are still many histone chaperone proteins and their interactors, whose roles in histone recycling are poorly understood. For example, the helicase lymphoid-specific (HELLS) and Aurora kinase B (AURKB) proteins, both identified in the MCM2 and POLE3/POLE4 interactomes, are known to regulate histone H3 phosphorylation and participate in chromatin remodeling [29, 30]. Bromodomain protein BRD4 has been shown to possess a histone chaperone activity in vitro [31] and interact with histone acetylation in vitro and in vivo [32]. SSRP1 has been previously recognized as a histone chaperone involved in chromatin regulation [33]. To validate our proteomic findings, we tested whether BRD4 and SSRP1, two central nodes in the MCM2 interactome, physically interacted with MCM2 in cells. Co-IP using an anti-MCM2 antibody in wild type 293 T successfully pulled down both BRD4 and SSRP1 (Supplementary Fig. 6). Importantly, this interaction was lost when using the MCM2-2A mutant 293 T cells, which carries alanine substitutions at residues critical for binding histone and fails to mediate parental histone transfer [14] (Supplementary Fig. 6). These results suggest that BRD4 and SSRP1 interacted with MCM2 in a histone-dependent manner, further supporting their potential involvement in the parental histone handoff pathway (Supplementary Fig. 6).

As a histone chaperone, NPM1 has been shown to interact with MCM2 and participate in the inheritance of chromatin [34]. Interestingly, in our POLE3-TurboID proteomics data, NPM1 exhibited a higher intensity than in the MCM2-TurboID proteomics data. We also validated this result using immunoprecipitation (Fig. 2C). It is possible that NPM1 collaborates with POLE3 in the leading strand in regulating chromatin inheritance. These observations underscore the complexity and diversity of histone chaperone functions, warranting further investigation into their specific roles in histone recycling during DNA replication in mammalian cells.

#### Polymerase subunits as potential histone chaperone network members

Notably, previous research have also showed that the POLE3/POLE4 subunit of leading-strand DNA polymerase ε and POLA1 primase subunit of lagging strand polymerase alpha complex participated in regulating parental histone recycling [7, 9]. Interestingly, we also identified other polymerase molecules, such as DNA polymerase delta subunit 3 (POLD3) and DNA polymerase alpha accessory subunit (POLA2), as members of the candidate histone chaperone networks associated with MCM2. Whether POLD3 and POLA2 function similarly in parental histone segregation remains to be determined.

Therefore, through the analysis of MCM2, POLE3 and POLE4 interactomes, we identified a potential candidate protein library involved in the process of parental histone segregation. This provides valuable insight into the MCM2- and POLE3/POLE4-based signaling pathways regulating parental histone distribution in mammalian cells, offering a foundation for further exploration of this critical mechanism.

### In mouse cells, comparative interactome and eSPAN analysis show POLD3, but not WDHD1, participates in parental histone distribution during DNA replication

#### Refining candidate proteins in parental histone segregation via comparative interactome analysis of MCM2-TurboID and MCM2-2A-TurboID

**Fig. 3:**
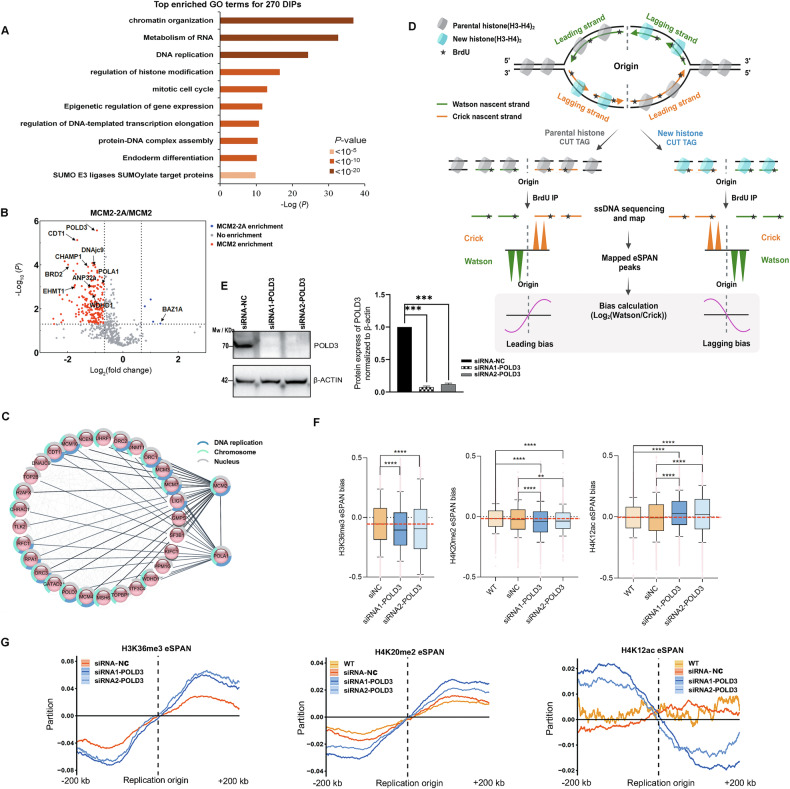
In mouse cells, comparative interactomes and eSPAN analysis reveal POLD3 participates in parental histone distribution during DNA replication. **A** Pathway enrichment analysis of differentially interactive proteins (DIPs) from TurboID proximity labeling of MCM2 and its histone binding mutant MCM2-2A in mouse NIH3T3 cells shows that the identified DIPs proteins align with MCM2’s role in regulating parental histone segregation. **B** A volcano plot of DIPs in (**A**) reveals 245 proteins were low-enriched in MCM2-2A interactomes. In MCM2-2A interactome, statistically significant proteins [a fold change (MCM2-2A/MCM2) ≥ 1.60 or ≤ 0.62 and *P* < 0.05] are shown as red (low-enriched) and blue (high-enriched) dots, while gray circles represent proteins without enrichment. The X-axis represents log_2_ (fold change), Y-axis means *P*-value (Tukey’s post-hoc test). **C** Network diagram of protein-protein interactions (PPIs) of DIPs highlights the potential candidate proteins closely associated with MCM2 and POLA1 based on STRING database, further narrowing the ranges of parental histone chaperone candidates. The interaction groups include DNA replication (dark blue), chromosome (light blue), and nucleus (grey). **D** Schematic illustrating how eSPAN identifies strand bias in the deposition of parental and new histones during DNA replication. **E** Western blot confirmed the successful knockdown of POLD3 expression in E14TG2a cells using siRNA. siRNA-NC represented cells transfected with a negative control siRNA. Protein expression levels of POLD3 were quantified by ImageJ. ***, *P* < 0.001 by the unpaired, two-tailed Student’s t-test; **F** Box plots showing the average bias of H4K20me2, H3K36me3, and H4K12ac eSPAN signals at initiation zones (*n* = 20,000) in WT, WT-siRNA-NC, and POLD3-knockdown mouse ES cells, each with two repeats shown. POLD3-knockdown cells showed significantly greater bias (Mann–Whitney U test). **, *P* < 0.01; ****, *P* < 0.0001. **G** Representative line plots illustrating average strand bias, with POLD3-knockdown cells displaying stronger deviation from baseline compared to WT. WT referred to wildtype ES cells.

To refine the selection of candidate proteins involved in MCM2-based parental histone segregation, we conducted TurboID proximity labeling on a mutant MCM2 (MCM2-2A) that contains mutations at Y81A and Y90A in histone-binding domain (HBD) of MCM2 [14]. These mutations disrupt the symmetric segregation of parental histones in mammalian cells. Based on the differing effects of MCM2 and MCM2-2A on histone segregation, we hypothesized that proteins enriched in the MCM2 interactomes but not in the MCM2-2A interactomes may represent potential histone chaperone candidates involved in this process. The MCM2-TurboID and MCM2-2A-TurboID fusion proteins were expressed at similar levels in mouse NIH3T3 cells (Fig. 2C), showing the comparability of the two interactomes. In our proximity labelling proteomics, we identified H3 and H4 proteins, which showed reduced enrichment in MCM2-2A-TurboID proteomics compared to MCM2-TurboID proteomics (Supplementary Fig. 7). This reduction aligns with the known function of the MCM2-2A mutant, which exhibits a loss of histone binding. For the comparative proteomics analysis, we set the following enrichment parameters to identify candidate proteins involved in MCM2-based parental histone segregation: (1) proteins were quantified at least twice across the four replicates; (2) MCM2/Control > 2 with a P-value < 0.05; (3) a fold change (MCM2-2A/MCM2) ≥ 1.60 or ≤ 0.62 with a P-value < 0.05. From this analysis, we identified 664 proteins, of which 270 were classified as differentially interacting. GO functional enrichment analysis revealed that the differentially interactive proteins are closely associated with processes such as chromatin organization, RNA metabolism, DNA replication, regulation of histone modification, and cell cycle regulation (Fig. 3A). This aligns with the role of MCM2 in regulating parental histone segregation and supports our hypothesis that comparing the MCM2 and MCM2-2A interactomes may reveal key proteins involved in this process. Of the 270 differentially interactive proteins between MCM2 and MCM2-2A, 245 were low-enriched in the MCM2-2A vs MCM2 interactome (Fig. 3B), which were considered as potential histone chaperone candidates. To further refine the selection of candidate proteins, we conducted interaction analysis of MCM2 and POLA1 using the STRING database and BiNGO, identifying 29 proteins as interactors among the low-enriched proteins (Fig. 3C) [35].

#### WDHD1 depletion does not affect parental histone distribution in mouse embryonic stem cells

Many of these low-enriched proteins in MCM2-2A interactomes are known to interact with histones, including WDHD1, DNAJC9, CHRAC1, and DNMT1. In yeast, the homolog of WDHD1, Ctf4, has been shown to facilitate the transfer of parental histones to lagging strands as part of the Mcm2-Ctf4-Pola1sigaling axis [3]. To determine whether WDHD1 plays a similar role in mammals, we knocked down WDHD1 expression with small interfering (si)RNAs in mouse E14TG2a embryonic stem cells (Supplementary Fig. 8A). Enrichment and sequencing of protein-associated nascent DNA (eSPAN) analysis (Fig. 3D) showed WDHD1-depleted and wild-type E14TG2a cells displayed a similar parental histone distribution pattern (Supplementary Fig. 8B). Therefore, the MCM2-WDHD1 pathway does not appear to function in parental histone segregation in mammalian cells.

#### POLD3 depletion biases parental histone segregation toward the leading strand

POLD3, a subunit of lagging strand DNA polymerase δ, was the protein whose low enrichment in the MCM2-2A vs MCM2 interactome had the most significant *P*-value (Fig. 3B), and it was also identified as a potential interaction partner of MCM2 and POLA1 (Figs. 2A, 3C). Therefore, to investigate whether POLD3 is involved in parental histone segregation in mammalian cells, we depleted POLD3 expression in mouse E14TG2a embryonic stem cells using siRNAs (Fig. 3E). We employed eSPAN to track parental H3-H4 histones (using H4K20me2- and H3K36me3-specific antibodies) and newly synthesized H3-H4 histones (using H4K12ac-specific antibodies) [7]. Although parental H3-H4 histones showed a slight leading-strand bias in wild-type mouse E14TG2a embryonic stem cells, they showed a strong leading-strand bias in POLD3-depleted cells (Fig. 3F, G, left). Conversely, whereas newly synthesized histones showed a nearly symmetrical segregation in wild-type E14TG2a cells, they showed a pronounced lagging-strand bias in POLD3-depleted cells (Fig. 3F, G, right). Flow cytometry and immunofluorescence analysis revealed that knocking down POLD3 expression in E14TG2a cells did not affect cell cycle progression (Supplementary Fig. 9). Cell viability assays also showed that treatment with POLD3-specific shRNA for 48 hours resulted in growth comparable to that of the non-knockdown control group (Supplementary Fig. 10A). DNA fiber assays further revealed only a mild reduction in replication fork speed (from ~1.25 kb/min to ~1.0 kb/min) upon siRNA-mediated knockdown of POLD3 (Supplementary Fig. 10B, C). These results suggest that the bias in parental histone segregation observed in POLD3-depleted cells is not caused by disruptions in the cell cycles. Together, these findings indicate that POLD3 participates in depositing parental H3-H4 histones onto the lagging strand during DNA replication in mammalian cells.

#### POLD3 as a new histone chaperone, directly interacts with (H3-H4)_2_ tetramer

**Fig. 4:**
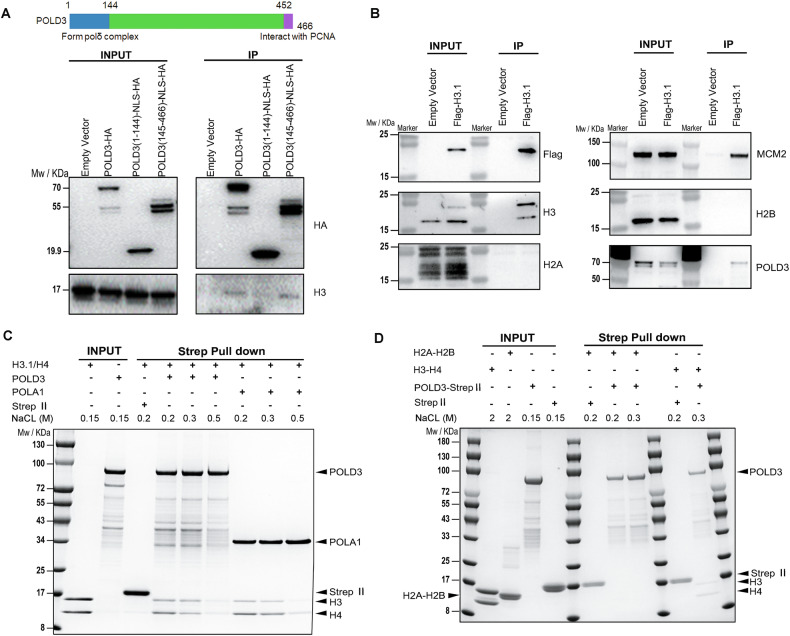
In human 293 T cells and outside of cells, POLD3 directly interacts with histone (H3-H4)_2_ tetramers. **A** Top: The known functional domain of POLD3; Bottom: Western blot analysis showing that POLD3-HA co-immunoprecipitated with histone H3 in 293 T cells. This assay further revealed that POLD3’s C-terminal fragment (amino acids 145-466), but not its-N terminal fragment (amino acids 1-144), interacted with histone H3. **B** Western blot using Flag-tagged histone H3 in 293 T cells demonstrated co-immunoprecipitation of POLD3, histone H3, and MCM2 from soluble cell extract, confirming their interaction. H2A and H2B were tested to exclude the interaction from nucleosomes. **C** In vitro interaction analysis using purified full-length strep-tagged POLD3 protein and purified (H3-H4)_2_ tetramer complex revealed strep-tagged POLD3 directly interacted with histone (H3-H4)_2_ tetramers, similar to the known parental histone chaperone, POLA1. Proteins and histones were stained with Coomassie Brilliant Blue (CBB). **D** In vitro interactions using purified full-length strep-tagged POLD3 protein, (H3-H4)_2_ tetramer complex and H2A-H2B dimer complex revealed that strep-tagged POLD3 did not interact with histone H2A-H2B dimers. Proteins and histones were stained with CBB.

The above eSPAN results showed the symmetric segregation of parental H3-H4 histones in POLD3-depleted cells was destroyed (Fig. 3F, G). To determine whether POLD3 acts as a histone chaperone during parental histone transfer in mammalian cells, we conducted co-IP assays between POLD3 and histone H3. Co-IP showed POLD3-hemagglutinin (HA) interacted with histone H3 in human epithelial-like kidney 293 T cells, consistent with a histone chaperone role (Fig. 4A). Next, we sought to dissect the different functional domains of full-length POLD3. It is known that POLD3’s N-terminal fragment (amino acids 1-144) interacts with POLD1, POLD2, and POLD4 to form a polymerase complex (PDB: 3E0J, 6TNY, 6TNZ, 6S1O), and its C-terminal fragment (amino acids 452-466) interacts with PCNA for DNA replication (PDB:1U76) (Fig. 4A, top) [36–38]. We hypothesized that the histone binding ability of POLD3 operates independently of the polymerase δ complex, which consists of POLD1, POLD2, POLD3, and POLD4. To test this hypothesis, we overexpressed two truncated POLD3-HA fragments (amino acids 1-144, 145-466) in 293 T cells. Co-IP showed POLD3’s C-terminal fragment (amino acids 145-466) in our experiment, but not its N-terminal fragment (amino acids 1-144), interacted with histone H3 (Fig. 4A, bottom), which was consistent with our hypothesis. To exclude potential interference from endogenous POLD3, we generated HeLa cell lines with doxycycline (Dox)-inducible shRNA-mediated knockdown of endogenous POLD3, followed by overexpression of either truncated POLD3-HA fragments or full-length POLD3-HA. Western blot analysis confirmed efficient depletion of endogenous POLD3 in cells expressing the truncated constructs, whereas in cells overexpressing full-length POLD3-HA, POLD3 levels remained largely unchanged due to persistent expression of the exogenous POLD3-HA protein (Supplementary Fig. 11A). Under these knockdown conditions, IP results showed that the C-terminal fragment retained its ability to bind histone H3, similar to the full-length protein, whereas the N-terminal fragment did not (Supplementary Fig. 11B). Collectively, these results demonstrate that the histone-binding function of POLD3 is primarily mediated by its C-terminal region and likely occurs independently of its incorporation into the DNA polymerase δ complex.

Furthermore, an IP assay using Flag-tagged histone H3 in 293 T cells also provided additional support for the connection between POLD3, histone H3, and MCM2 (Fig. 4B). In the co-IP assay using Flag-tagged histone H3, we employed ethidium bromide (EB) to reduce the interaction between proteins and DNA and did not detect H2A and H2B (Fig. 4B). These findings indicate that the interaction between POLD3 and H3 does not originate from nucleosomes.

Parental histone heritance is based on the form of (H3-H4)_2_ tetramers, which interact with histone H2A-H2B dimer to deposit back into new replicated DNA strands and form the nucleosome [39]. Thus, to confirm POLD3 as a histone chaperone to directly interact with (H3-H4)_2_ tetramers, we conducted a streptavidin pulldown assay using purified POLD3 and histone (H3-H4)_2_ tetramers. The pulldown assay showed POLD3 directly interacted with histone (H3-H4)_2_ tetramers, similar to the known parental histone chaperone, POLA1 (amino acids 1-200), which has been shown as parental histone binding domain [7] (Fig. 4C and Supplementary Fig. 12). Binding between POLD3 and histone (H3-H4)_2_ tetramers remained detectable even under high salt concentrations, suggesting a strong and stable interaction. A further pull-down assay using purified POLD3 and histone H2A-H2B dimers showed POLD3 cannot interact with histone H2A-H2B dimers (Fig. 4D). Therefore, our findings indicate that POLD3 functions as a novel histone chaperone, capable of directly interacting with (H3-H4)₂ tetramers, but not with H2A-H2B dimers.

### MCM2, POLD3 and (H3-H4)_2_ tetramer interacted with each other to form the protein complex

#### The interaction between MCM2 and POLD3

**Fig. 5:**
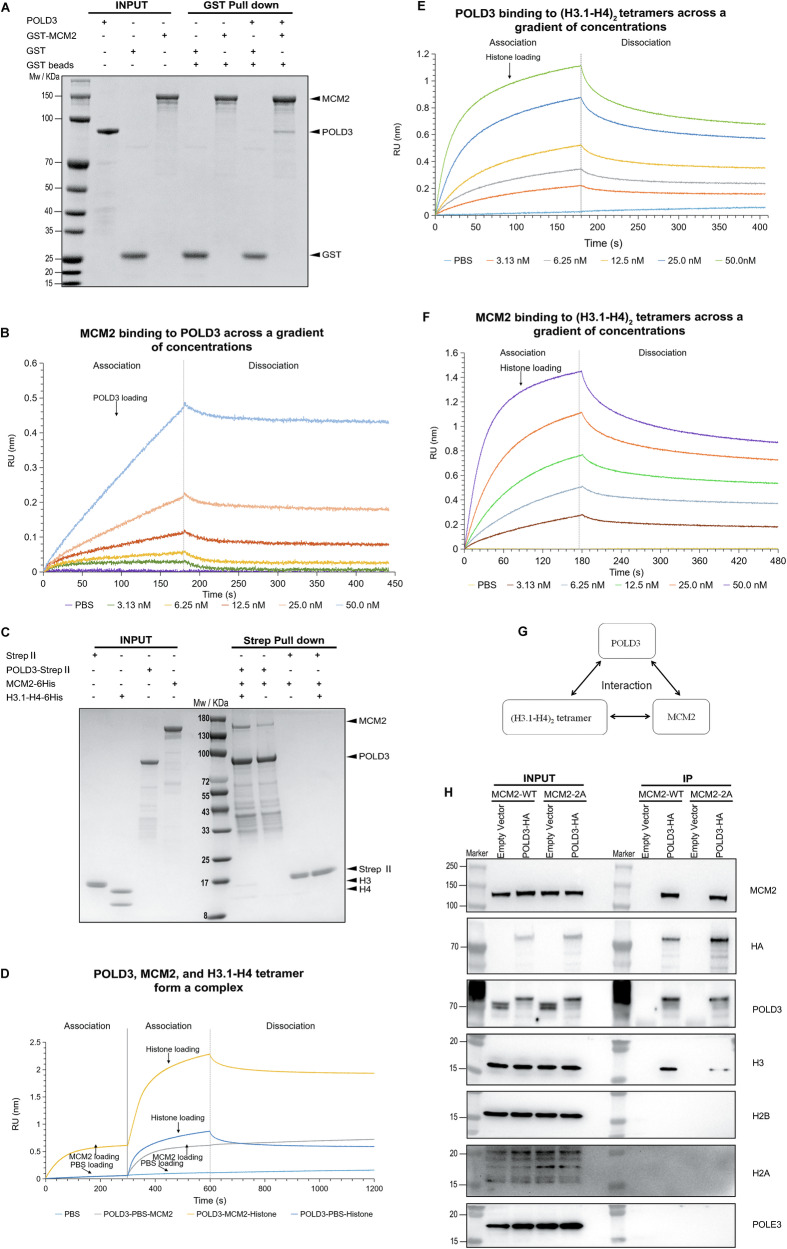
Interaction of MCM2, POLD3, and (H3-H4)_2_ tetramers to form a protein complex. **A** Pulldown assay using purified GST-MCM2 and POLD3 revealed a subtle yet clear interaction between MCM2 and POLD3 in vitro; **B** Biolayer interferometry (BLI) assays measuring the specific binding of POLD3 (at concentrations of 0, 3.13, 6.25, 12.5, 25.0, and 50.0 nM) to immobilized MCM2 confirmed a concentration-dependent interaction between MCM2 and POLD3. **C** A pulldown assay performed with purified strep-tagged POLD3 showed that POLD3, MCM2, and the histone (H3.1-H4)_2_ tetramers formed a protein complex in vitro; **D** BLI assays using immobilized POLD3 further supported the formation of a complex containing POLD3, MCM2 and the histone (H3.1-H4)_2_ tetramers. **E** BLI assays measuring the specific binding of (H3.1-H4)_2_ tetramers (at concentrations of 0, 3.13, 6.25, 12.5, 25.0, 50.0 nM) to immobilized POLD3 showed a strong, concentration-dependent interaction between POLD3 and the (H3.1-H4)_2_ tetramers. **F** BLI assays measuring the specific binding of (H3.1-H4)_2_ tetramers (at concentrations of 0, 3.13, 6.25, 12.5, 25.0, 50.0 nM) to immobilized MCM2 indicated a strong, concentration-dependent interaction between MCM2 and the (H3.1-H4)_2_ tetramers. **G** Schematic illustrating the triangular interaction between MCM2, POLD3 and (H3-H4)_2_ tetramers. **H** Pull-down assay of HA-tagged POLD3 in soluble cell extracts from wild type 293 T cells and MCM2-2A mutant 293 T cells showed that POLD3-HA pulled down significantly less histone H3 and did not interact with histones H2A and H2B in MCM2-2A mutant vs wild-type cells. Western blot was representative of two biological replicates.

Having shown that Flag-tagged histone H3, POLD3, and MCM2 from soluble 293 T cell extract could co-immunoprecipitate, we wanted to further confirm the directly interactive relationship between POLD3 and MCM2. To do so, we performed a pulldown assay focusing on purified GST-tagged MCM2 and purified POLD3. This assay revealed a subtle but clear and direct interaction between MCM2 and POLD3 (Fig. 5A). We also performed biolayer interferometry (BLI) assays to further validate the interaction. Using immobilized MCM2, the BLI analysis demonstrated that MCM2 interacted with POLD3, with an equilibrium dissociation constant (K_D_) value of just 10.20 nM (Fig. 5B). To test the effect of the histone-binding domain (HBD) of MCM2 (MCM2-HBD, amino acids 51-160) on the interaction between MCM2 and POLD3, we purified MCM2-HBD and MCM2-2A histone-binding domain (MCM2-2A-HBD) to perform BLI assays. Using immobilized POLD3, the BLI data demonstrated that POLD3 interacted with MCM2, but did not interact with MCM2-HBD or MCM2-2A-HBD (Supplementary Fig. 13). Collectively, these assays confirm the interaction between MCM2 and POLD3 and demonstrate that it occurs independent of MCM2’s histone-binding domain.

#### Triangular interaction among MCM2, POLD3, and (H3-H4)_2_ tetramers

Our data indicate that POLD3, rather than WDHD1, functions as a histone chaperone responsible for regulating the symmetric inheritance of parental histones in mammalian cells, similar to the role of MCM2 [2]. To further analyze the directly interactive relationship between MCM2, POLD3 and (H3.1-H4)_2_ tetramer, we conducted a pulldown assay of all these proteins. The results indicate that purified strep-tagged POLD3, MCM2, and histone (H3.1-H4)_2_ tetramers directly form a protein complex (Fig. 5C). Additional BLI assays also supported the existence of a protein complex containing POLD3, MCM2 and the histone (H3.1-H4)_2_ tetramer (Fig. 5D). In addition, immunoprecipitation experiments using an anti-MCM2 antibody in 293 T cells further confirmed that MCM2 was closely associated with both POLD3 and histone H3 in vivo (Supplementary Fig. 14). We hypothesized that the interaction between these proteins occurs in a mutually dependent triangular manner. To test this hypothesis, we conducted additional BLI assays. Our BLI results showed that the interaction between POLD3 and (H3.1-H4)_2_ tetramers was very strong, K_D_ value of which is just 2.70 nM, similar to the interaction between MCM2 and (H3.1-H4)_2_ tetramers, K_D_ value of which is just 2.17 nM (Fig. 5E, F). The K_D_ values obtained from the BLI assays—10.20 nM for the interaction between MCM2 and POLD3, 2.17 nM for MCM2 binding with (H3.1-H4)_2_ tetramers, and 2.70 nM for POLD3 binding with (H3.1-H4)_2_ tetramers—suggest that both MCM2 and POLD3 bind more readily to (H3.1-H4)_2_ tetramers than to each other. Therefore, all of the findings confirm a triangular interaction between MCM2, POLD3 and (H3-H4)_2_ tetramers (Fig. 5G).

#### Impact of MCM2 histone-binding ability on POLD3–histone association

Based on the above interaction results between MCM2, POLD3, and (H3-H4)_2_ tetramers, we hypothesize that although the histone-binding ability of MCM2 does not affect the interaction between MCM2 and POLD3, it may influence the binding of POLD3 to histones. Due to the known histone binding site of MCM2-2A (Y81A and Y90A) for the lack of histone interaction, we set out to test the hypothesis in mammalian cells. We overexpressed POLD3-HA in wild-type and MCM2-2A mutant 293 T cells. POLD3-HA did not pull down POLE3, a subunit of the polymerase ε complex at replication forks, used as a negative control, in either cell type (Fig. 5H). In MCM2-2A mutant cells, in which parental histones cannot be transferred to downstream proteins, POLD3-HA pulled down significantly less histone H3 than in wild-type cells (Fig. 5H). This finding suggests that the histone binding ability of MCM2 might affect the interaction between POLD3 and histone H3. Additionally, this assay showed that POLD3-HA did not co-immunoprecipitate histones H2A or H2B in either wild-type or MCM2-2A mutant 293 T cells, suggesting that the histone H3 associated with POLD3 is not derived from intact nucleosomes in mammalian cells (Fig. 5H). These findings support our hypothesis that the inability of MCM2 to bind histones disrupts the interaction between POLD3 and histones, which are not associated with the nucleosome.

Finally, we also investigated the degree of strand bias at different replication origins in MCM2-2A mutant and POLD3-knockdown mouse E14TG2a embryonic stem cells. Interestingly, the results showed a high degree of correlation between the degree of strand bias at different replication origins, as measured by eSPAN data, between the two cell types (Supplementary Fig. 15).

All of these findings suggest that POLD3, in complex with MCM2 and histone (H3-H4)_2_ tetramers, functions as a novel histone chaperone involved in the regulation of parental histone segregation in mammalian cells.

## Discussion

In this study, our goal was to identify novel proteins involved in the symmetric distribution of parental histones to the lagging and leading strands of newly synthesized DNA in mammalian cells. Previous reports have shown that MCM2, POLE3 and POLE4 act as histone chaperones, playing critical roles in the segregation of parental histones [2, 9, 40]. Through MCM2, POLE3, and POLE4 interactomes, we have identified a candidate protein library associated with the MCM2- or POLE3/POLE4-driven signaling pathways that regulate parental histone transfer in mammalian cells. Our interactome data in mouse cells implicated POLD3, a subunit of lagging strand DNA polymerase δ, as a protein involved in parental histone transfer. We conducted a variety of experiments in mouse and human cells, as well as outside cells, to demonstrate that POLD3 directly interacts with histone (H3-H4)_2_ tetramers, forms a protein complex with MCM2 and histone (H3-H4)_2_ tetramers, and participates in parental histone transfer during DNA replication. Other researchers have proposed that POLD3 plays an important role in DNA damage repair by regulating the 53BP1, RIF1, ATR, and ATM pathways [41]. However, to the best of our knowledge, our findings provide the first evidence that POLD3 may participate in parental histone distribution in mammalian cells. Interestingly, a recent study also reported that a POLD3 ortholog in *Saccharomyces cerevisiae*, Pol32, was also shown to act as a parental histone chaperone [42].

POLD3 is not the only DNA polymerase subunit that has been found to play an important role in parental histone transfer during DNA replication. POLA1, a subunit of the lagging-strand DNA polymerase α complex, and POLE3 and POLE4, subunits of the leading-strand DNA polymerase ε, have also been reported to regulate the symmetric segregation of parental histone in mouse cells [7, 9]. POLA1 and POLD3 may be instrumental in transferring parental histones to the lagging strand, while POLE3/POLE4 is likely to primarily participate in the inheritance of parental histone on the leading strand.

Our data also suggests that POLD3, MCM2, and histone (H3-H4)_2_ tetramers interact in a bi-directional triangular relationship, forming a protein complex within the cells. Notably, the binding affinities of MCM2 with (H3-H4)_2_ tetramers and POLD3 with (H3-H4)_2_ tetramers are stronger than the interaction between MCM2 with POLD3. This suggests that the three-protein complex may dissociate into separate complexes, such as POLD3 bound to (H3-H4)_2_ tetramers or MCM2 bound to (H3-H4)_2_ tetramers. Interestingly, we discovered that the ability of POLD3 to interact with histone H3 was impaired in MCM2-2A mutant human 293 T cells, indicating that MCM2’s histone binding ability influences the interaction between POLD3 and histone proteins. These findings imply a process in which (H3-H4)_2_ tetramers are transferred from MCM2 to POLD3 in mammalian cells. Further biochemical experiments are required to confirm this process.

Homologs of the mammalian protein WDHD1 have been reported to function in parental histone distribution and heterochromatin propagation including Ctf4 protein from *S. cerevesiae* and Mcl1 protein from the fission yeast *Schizosaccharomyces pombe* [43]. In addition, in *S. cerevisiae*, Mcm2-Ctf4-Pola1 signaling axis has been showed to play a role in the process of parental histone distribution [3]. However, different from Ctf4 and Mcl1, WDHD1 has been reported to anchor lagging-strand DNA polymerase alpha (Polα) by SeqB domain and connect with CMG complex by unknown participants other than Go Ichi Ni San [44]. Besides, instead of Ctf4 trimer, dimeric WDHD1 link the CMG complex to Polα [45, 46]. We also showed that in mouse cells, knocking down WDHD1 expression did not affect parental histone distribution patterns, unlike the action of Ctf4 and Mcl1 in yeast. Our data suggest that the MCM2-POLD3-(H3-H4)_2_ tetramer complex, rather than MCM2-WDHD1 signaling axis, may play an essential role in symmetric segregation of parental histones in mammalian cells. However, this data does not rule out the possibility that WDHD1 is involved in the parental histone transfer process. They highlight the differences that exist between yeast and mammalian cells, illustrating the need for caution in translating findings from the former to the latter.

Although we conducted extensive assays to elucidate the role of POLD3 in parental histone transfer in this study, the interactome data for MCM2, POLE3, and POLE4 collected in this study may also include other known histone chaperones or potential candidates that warrant further investigation. For example, SSRP1 and DNAjc9 were enriched in both of the MCM2 and POLE3/POLE4 interactomes. SSRP1 has been previously recognized as a histone chaperone involved in chromatin regulation [33]. Our co-immunoprecipitation using an anti-MCM2 antibody successfully pulled down SSRP1 in wild type 293 T cells. Importantly, this interaction was lost in MCM2-2A mutant cell lines, which carry alanine substitutions at residues critical for histone binding and fail to mediate parental histone transfer. DNAJC9 has been reported to interact with histone H3-H4 dimers and MCM2 [47]. These proteins might also be important participants in histone transfer during DNA replication. Further research on these proteins may provide valuable insights into the mechanisms of histone transfer and segregation.

In conclusion, this study provides a potential candidate protein library involved in the MCM2- or POLE3/POLE4-based signaling pathway of parental histone segregation. Our findings establish that POLD3 functions as a parental histone chaperone, contributing to the symmetric distribution of parental histones during DNA replication in mammalian cells. Given the complexity of mammalian cell differentiation compared to yeast, proper inheritance of parental histones is crucial for maintaining or altering cellular fate of mammalian cells [4, 48]. This research fills a gap in our understanding of the mechanism underlying parental histone transfer to the new synthesized DNA strand in mammalian cells. Our data support the existence of the protein complex composed of MCM2, POLD3 and (H3-H4)_2_ tetramers, which interact in a triangular relationship. MCM2-7 helicase unwinds double-stranded DNA and DNA polymerase δ synthesize DNA strand during replication [49]. Based on their functional role at the replication fork, we hypothesize MCM2 may transfer parental histone to POLD3. Future studies are needed to further elucidate this process.

### Limitations of the study

This study has several limitations. First, in our TurboID proximity labelling experiments, we used a lentiviral system to overexpress bait proteins rather than using CRISPR to generate TurboID knock-in cells. Overexpression by the lentiviral system may generate false positive results for bait-proximal proteins. We believe this potential problem is unlikely to affect our results, as we expressed bait proteins at levels lower than their endogenous counterparts (Supplementary Fig. 16). Second, in our study, due to the technical limitation, we did not identify the specific sites on POLD3 that mediate its interactions with histones and MCM2. Identifying these critical amino acid sites would enable the generation of stable cell lines with defective POLD3, facilitating further investigations into its role as a histone chaperone and its interaction with MCM2. Finally, future studies should also focus on exploring the impact of POLD3’s histone chaperone function on genetic development and cell differentiation in mammals, which would provide deeper insights into its broader biological roles.

## Materials and methods

### Cell culture

NIH3T3 (mouse embryonic fibroblast), HeLa (henrietta lacks cells) and 293 T (human epithelial-like kidney) cells were obtained from ATCC and were cultured in Dulbecco’s Modified Eagle Medium (DMEM) supplemented with 100 mg/ml streptomycin, 100 U/ml penicillin, and 10% fetal bovine serum (PAN-Biotech, Adenbach, Bavaria, Germany) at 37 °C and 5% CO_2_.

Mouse E14TG2a embryonic stem cells were a gift from the Institute of Biophysics, Chinese Academy of Sciences. E14TG2a cells were cultured on gelatin-coated dishes in complete medium containing DMEM/F-12, 15% (v/v) fetal bovine serum (Gibco; Thermo Fisher Scientific, Inc., Waltham, MA, USA), 1 mM sodium pyruvate (Gibco; Thermo Fisher Scientific, Inc., Waltham, MA, USA), 1% penicillin/streptomycin (Thermo Fisher Scientific, USA), 1% MEM nonessential amino acids (Gibco; Thermo Fisher Scientific, Inc., Waltham, MA, USA), 2 mM GlutaMAX (Gibco, Thermo Fisher Scientific, USA), 10 ng/mL mouse leukemia inhibitory factor (Millipore, Billerica, MA), and 55 μM β-mercaptoethanol (Sigma-Aldrich, St. Louis, MO, USA) in a 37 °C incubator with a humidified, 5% CO_2_ atmosphere. Cells were passaged using trypsin-EDTA (Gibco; Thermo Fisher Scientific, Inc., Waltham, MA, USA).

### Construction of 293 T MCM2-2A mutant cells

293 T MCM2-2A mutant cells were created as previously described [5]. Briefly, Tyr81 and Tyr90 of MCM2 were mutated to Ala by CRISPR–Cas9 system following the standard protocol [50]. Oligonucleotides were synthesized and inserted into pX459 vector (Addgene cat. no. 48139). 293 T cells were transfected with pX459 vector and a single-stranded oligonucleotide donor using electroporation. After transfection, 293 T cells were selected using 2 μg/mL puromycin (No.540411, Sigma-Aldrich, St. Louis, MO, USA) for 4 days and replated as single cells. Single-cell clones were picked after 5–10 days and expanded for genotyping.

### Plasmids construction

Mouse MCM2-TurboID-3xHA pET28b plasmid and MCM2-2A-TurboID-3xHA pET28b plasmid were obtained from YouBio company (HuNan, China). *MCM2-TurboID-3xHA* and *MCM2-2A-TurboID-3xHA* fragments were amplified by PCR using the above plasmids, digested with restriction endonucleases EcoRI/BamHI, and ligated into pcDNA3.

*POLE3* and *POLE4* were amplified by PCR from E14TG2a cell cDNA and inserted into MCM2-TurboID-pcDNA3 plasmids in place of *MCM2*.

*H3.1*, *POLD3*, and truncated versions of *POLD3* (DNA encoding amino acids 1-144 and 145-466) were amplified by PCR from cDNA obtained from a human breast adenocarcinoma cell line, MCF-7. Full and truncated *POLD3* fragments were inserted into pcDNA3 plasmids with 3x NLS and 3x HA tags. *H3.1* was inserted into pcDNA3 plasmids with 3x FLAG.

The full-length human *POLD3* gene and a truncated version of the *POLA1* gene (amino acids 1-200) were synthesized and codon-optimized by Genescript company in China. Both genes were inserted into the plasmid pET28b which included a 6x His tag at the N terminus and a twin-strep tag at the C terminus to facilitate protein purification.

The genes encoding MCM2, MCM2-HBD (amino acids 51-160) and MCM2-2A-HBD were amplified from cDNA derived from human MCF-7 breast adenocarcinoma cells. These genes were inserted into pGEX-C5X plasmids, where they were fused with a GST tag at the N terminus and a His tag at the C terminus for subsequent protein purification.

### Creation of mouse NIH3T3 cell lines stably expressing TurboID fusion proteins

The lenti-packaging plasmid psPAX2, the lenti-envelope plasmid pMD2G and target plasmids containing pcDNA3, MCM2-TurboID-pcDNA3, MCM2-2A-TurboID-pcDNA3, POLE3-TurboID-pcDNA3, or POLE4-TurboID-pcDNA3 were co-transfected into human 293 T cells using lipofectamine 3000 (No. L3000001, Invitrogen, USA) to produce lentiviral particles for the overexpression of the respective proteins, following the manufacturer’s protocol. Culture supernatants containing virus particles were harvested at 48- and 72-h post-transfection and filtered through 0.45-μm pore-size filters. Then, mouse NIH3T3 cells were infected with these supernatants for 48 h. Stably transfected cells were selected by adding 2 μg/mL puromycin (No.540411, Sigma-Aldrich, St. Louis, MO, USA) to the medium, which was changed every 2 days. Selection was terminated when uninfected control cells were completely dead. The five stably expressing cell lines were designated as NIH3T3-Control which just expresses puromycin N-acetyltransferase (Pac), POLE3-TurboID-NIH3T3, POLE4-TurboID-NIH3T3, MCM2-TurboID-NIH3T3, and MCM2-2A-TurboID-NIH3T3.

### Creation of human 293 T cell lines stably expressing tagged H3.1, POLD3, and truncated versions of POLD3

The psPAX2, pMD2G and target plasmids containing H3.1-Flag-pcDNA3, POLD3-HA-pcDNA3, or truncated versions of POLD3-HA-pcDNA3, were co-transfected into human 293 T cells using lipofectamine 3000 to generate lentiviral particles for protein overexpression of H3.1, POLD3, or truncated versions of POLD3, according to the manufacturer’s instructions. Culture supernatants containing virus particles were harvested. Then, based on the requirement of the assays, wild-type or MCM2-2A mutant 293 T cells were infected with these supernatants for 48 h. Stably transfected cells were selected by adding 2 μg/mL puromycin to the medium, which was changed every 2 days. Selection was terminated when uninfected control cells were completely dead. The stably expressing cell lines were designated as POLD3-HA-293T, POLD3-HA-293T(MCM2-2A), H3.1-Flag-293T, POLD3(1-144)-293T and POLD3(145-466)-293T.

### Establishment of HeLa cell lines expressing full-length and truncated forms of POLD3 with doxycycline-inducible knockdown of endogenous POLD3

To generate inducible POLD3 knockdown cell lines, a modified PiggyBac transposon plasmid containing a TRE-driven shRNA targeting human *POLD3* and a separate EF1α promoter driving GFP-T2A-puromycin expression (PiggyBac-TRE-shPOLD3-EF1α-TetR-GFP-T2A-Puro) was constructed. The shRNA sequence targeting POLD3 was: 5′- GGCCTCTGTTCAATACTGA -3′.

The Hela cells were co-transfected with this PiggyBac construct plasmid and the transposase-expressing plasmid (PBase) using Lipofectamine 3000, following the manufacturer’s instructions. At 48 h post-transfection, the culture medium was supplemented with 5 μg/mL puromycin for stable selection, and medium was changed every 2 days. GFP fluorescence was monitored to confirm transgene integration. Puromycin selection was terminated when uninfected control cells were fully eliminated. Subsequently, based on the doxycycline-inducible POLD3 knockdown cell line (shPOLD3-Hela) described above, we established cell lines stably overexpressing full-length POLD3 and truncated POLD3. The generation of these overexpression cell lines followed the same procedure as that used for establishing POLD3-overexpressing 293 T cells described previously.

To induce shRNA expression, cells were treated with 1 μg/mL doxycycline for 48 h. The knockdown efficiency of POLD3 was validated by western blot. These cell lines were designated as POLD3-HA-shPOLD3-Hela, POLD3(1-144)- shPOLD3-Hela and POLD3(145-466)- shPOLD3-Hela.

### Immunofluorescent staining to optimize TurboID proximity labeling process

NIH3T3 cells with expression of MCM2-TurboID-HA were incubated in DMEM medium with normal FBS or dialyzed FBS for 14 days and treated with 500 μM biotin in dimethyl sulfoxide (DMSO) for various durations (0 min, 10 min, 30 min and 60 min). Small molecules such as salt, biotin and various low-molecular-weight proteins have been removed from dialyzed FBS. Next, the medium was removed, and cells were coated with a layer of 4% formaldehyde diluted with 1x PBS at room temperature (RT) for 20 min. Cells were incubated with 100 nM glycine for 5 min and washed three times with 1x PBS for 5 min each time. Specimens were then incubated in sealing buffer (1x PBS/5% BSA/0.3% Triton™ X-100) at RT for 60 min. After that, we incubated the cells overnight at 4°C with Streptavidin−Cy3™ according to the dilution ratio recommended in the instruction. Cells were rinsed three times with 1x PBS containing 0.1% Tween-20, for 5 min each time. DAPI was prepared with anti-fluorescence quenching reagent from Beyotime Biotechnology, and confocal microscopy was employed to observe cells after staining. All experiments were independently repeated three times.

### TurboID-based proximity labeling proteomics analysis for POLE3, POLE4, MCM2, and MCM2-2A in mouse NIH3T3 cells

#### Cell synchronization of bait-TurboID cell lines

As parental histone recycling occurs during DNA replication, we synchronized bait-TurboID cells in S-phase using a double thymidine block treatment (Supplementary Fig. 17). NIH3T3 cells overexpressing POLE3-TurboID, POLE4-TurboID, MCM2-TurboID, and MCM2-2A-TurboID were incubated in 15-cm culture dishes in DMEM medium with dialyzed FBS for 14 days. After being cultured for 14 days, cells were digested and seeded in 15-cm culture dishes and incubated with 3 mM thymidine for 18 h. Thymidine was then replaced by DMEM medium with dialyzed FBS for 7–8 h. Cells were treated again with 3 mM thymidine for 12 h. Finally, cells were released from G1 phase by washing them with pre-warmed 1x PBS and incubated in fresh pre-warmed DMEM media with dialyzed FBS. Cells were collected 0-, 4-, 8- and 12 h post-release for cell cycle analysis, conducted with DNA and PI staining using flow cytometry. This experiment was performed with three biological replicates.

At 4-hour post release from G1 phase, most cells have entered S phase, and expression of bait proteins did not impact cell cycle duration (Supplementary Fig. 17). After considering labeling efficiency during biotin incubation, we decided to use cells released from G1 phase for 3.5–4.5 h for our TurboID proximity labeling experiment.

#### Sample preparation

After cell cycle synchronization, NIH3T3 cells expressing bait-TurboID proteins were treated with 500 μM biotin in DMSO for 1 h. Biotin labeling was stopped by transferring the cells to ice and washing them five times with ice-cold PBS. Next, cells were collected, lysed with RIPA lysis buffer (50 mM Tris-HCl pH 7.5, 150 mM NaCl, 1.5 mM MgCl2, 1 mM EGTA, 0.1% SDS, 1% NP-40, 0.4% sodium deoxycholate, 1 mM DTT, 1 mM PMSF, and protease inhibitor cocktail tablet) on ice for 10 min and sonicated for 5 min. Samples were centrifuged for 10 min at 12,000 × *g*, and protein concentrations were calculated using the BCA kit (ThermoFisher Scientific, Rockford, IL, USA). To pull down biotinylated proteins, we combined 800 μg proteins with 200 μL streptavidin-coupled magnetic beads slurry, which had been previously washed and equilibrated. The mixture was put on a rotating wheel for 3 h at 4 °C. Then, the supernatant was removed and beads were washed for 5 min every time at RT on a rotating wheel, one time with wash buffer 1 [50 mM Tris-HCl (pH 7.5), 1% SDS], three times with wash buffer 2 [50 mM Tris-HCl (pH 7.5), 150 mM NaCl, 1.5 mM MgCl2, 1 mM EGTA, 0.1% SDS, 1% NP-40, 1 mM DTT], twice with wash buffer 3 (8 M urea), twice with wash buffer 4 (30% ACN), and three times with buffer 5 (20 mM NH_4_HCO_3_). A fraction of the beads (5%) was boiled in laemmli buffer for western blotting to confirm successful biotin labeling of the proteins. The remaining beads were subjected to liquid chromatography coupled with tandem mass spectrometry (LC-MS/MS) to identify proteins around the bait proteins. Four biological replicates were performed for sample preparation to ensure reliability and reproducibility of the data.

#### LC-MS/MS

To carry out on-bead trypsin digestion, the beads were reduced and alkylated for 30 min at 37 °C using 10 mM TCEP and 40 mM 2-chloroacetamide. After alkylation, mass spectrometry grade trypsin (Promega, USA) was added for overnight digestion at 37 °C. The digestion was quenched with formic acid at a final concentration of 0.1% (vol/vol). Beads were removed by magnetic grate. Acidifying peptide samples were desalted on C18 StageTips, dried completely in a SpeedVac centrifuge at 45 °C, and stored at -80°C. Desalted peptides were separated at 300 nL/min on an analytical column (15 cm × 75 μm inner diameter, No.164534, ThermoFisher Scientific, Rockford, IL, USA) with pre column (Acclaim PepMap 100 75 μm × 2 cm C18, No.164535, ThermoFisher Scientific, Rockford, IL, USA). Nano-LC was performed using the UltiMate 3000 nanosystem (ThermoFisher Scientific, Rockford, IL, USA), with mobile phases buffer A (H_2_O/0.1% FA) and buffer B (80% ACN/0.1% FA/H_2_O). A 60 min gradient was applied as follows: 3–8% buffer B for 3 min; 8%–28% buffer B for 30 min; 28%–38% buffer B for 10 min; 38%–99% buffer B for 1 min; and 99% buffer B for 4 min.

The UltiMate 3000 nanosystem was connected online to a Thermo Scientific Q-Exactive HF mass spectrometer (ThermoFisher Scientific, Rockford, IL, USA) operating in positive ion and data-dependent acquisition modes. The automatic gain control (AGC) target value and maximum injection time for the full MS scan were set as 3 × 10^6 ^ions and 20 ms, respectively. Each MS scan was acquired at high resolution (60,000 at m/z 200) with a mass range of 350–2000 m/z. The AGC target value and the maximum injection time for MS/MS were set as 5 × 10^4^ and 50 ms, respectively. The dynamic exclusion time was 30 s. MS/MS spectra were captured at resolution 15,000 at m/z 200. Nano electrospray ion source settings included a spray voltage of 2.1 kV without sheath gas flow, and the capillary temperature was set to 275  °C.

#### Analysis of MS data

First, we identified all proteins in the MS data for each bait protein (POLE3-TurboID, POLE4-TurboID, MCM2-TurboID, and MCM2-2A-TurboID). Raw MS data were analyzed with MaxQuant software, version 1.6.10.43. The UniProt mouse protein database (updated in April 2019) was used for the database search. Label-free quantification was used, and 1% false discovery rate (FDR) filtering and matching between runs were enabled. The database search parameters were as follows: (1) trypsin and lysC were chosen as digestion enzymes, and no more than two missed cleavages were permitted; (2) cysteine carbamidomethylation was set as a fixed modification, and methionine oxidation was chosen as a variable modification.

Next, to identify proteins enriched around MCM2, POLE3, and POLE4, we performed a proteomics analysis. Enrichment criteria were set as follows: (1) a fold-change greater than 2 compared to control (MCM2/Control >2, POLE3/Control >2, or POLE4/Control >2); (2) detection in at least two of four replicates; (3) *P*-value < 0.05. We integrated the POLE3 and POLE4 interactomes, considering proteins enriched in both as potential histone chaperone candidates. Additionally, we also performed a proteomics analysis to identify differentially enriched proteins between MCM2-2A and MCM2 interactomes. For the comparative proteomics analysis, enrichment parameters were set as (1) proteins were quantified at least twice across the four replicates; (2) MCM2/Control > 2 with a *P*-value < 0.05; (3) a fold change (MCM2-2A/MCM2) ≥ 1.60 or ≤ 0.62 with a *P*-value < 0.05. These criteria ensured the identification of proteins with significant changes in abundance related to histone distribution. For all proteomics data, the mean ± standard deviation (SD) was calculated for each group. Variance among groups was evaluated by visual inspection and confirmed using Levene’s test, and data were found to be approximately homogeneous. Statistical significance was assessed using one-way ANOVA, followed by Tukey’s post-hoc test for pairwise comparisons. These analyses assume approximate normal distribution and homogeneity of variance across groups. Principal component analysis, volcano plots and heatmaps were generated with R statistical software. Volcano plots displayed mean values of log_2_(fold change) and their corresponding P-values for the four biological triplicates. Our network analysis of histone chaperones around protein MCM2 and proteins POLE3/POLE4 during DNA replication was visualized using Cytoscape v.3.8.0. Gene Ontology (GO) enrichment analysis for enriched proteins was performed using Metascape (https://metascape.org) [51] to identify key biological processes and molecular functions associated with the candidate proteins. P-values for GO enrichment analysis were calculated based on the cumulative hypergeometric distribution. This analysis provided insights into the potential roles of these proteins in cellular processes related to parental histone inheritance. All MS proteomics data were uploaded via the PRoteomics IDEntifications database (PRIDE: PXD048030), publicly accessible at http://proteomecentral.proteomexchange.org. Username: reviewer_pxd048030@ebi.ac.uk. Password: 0Xnyh3Fg.

### siRNA knockdown of WDHD1 and POLD3 in mouse E14TG2a embryonic stem cells

We obtained siRNA sequences targeting mouse WDHD1 [5’-CCACGACCUGCUGUAGCUAUAUUAU-3’ (siRNA1-WDHD1), 5’-UCUGUUGGGAUUAUUCGCUGCUAUA-3’ (siRNA2-WDHD1)] and POLD3 [5′-CAGUAGUGAGAGAAGAUAAAC-3′ (siRNA1-POLD3), 5′-AGAAAGUAUGAACAGUCAAAU-3′ (siRNA2-POLD3), as well as a negative control siRNA sequence [5′-UUCUCCGAACGUGUCACGUTT-3′ (siRNA-NC)] from Gene Pharma (Shanghai, China). To transfect siRNA into E14TG2a cells, 100 nM siRNA was incubated with Lipofectamine 3000 for 20 min and diluted in Opti-MEM (No. 31985070, Life Technologies, Carlsbad, CA). Following that, freshly passaged cells (1.8 × 10^6^) and the siRNA-lipofectamine mixture were plated in six-well cell culture plates. After 6 h incubation, the transfection medium was replaced with fresh DMEM complete medium. Forty-eight hours after transfection, proteins were extracted, and the efficiency of siRNA knockdown was evaluated by western blot. β-ACTIN was used as a loading control. Detailed antibody information was listed in Supplementary Table 1. All experiments were independently performed three times. Protein expression levels were quantified by ImageJ and presented as mean ± standard deviation (SD). Variance between groups was assessed by visual inspection of SD and confirmed to be similar, and data were assumed to be approximately normally distributed, allowing the use of two-tailed Student’s t-test for comparisons between two groups.

### EdU cell proliferation assay using mouse E14TG2a cells transfected with siRNA

To measure cell proliferation in E14TG2a cells transfected with siRNA, we used a BeyoClick™ EDU-488 Imaging Kit (No.C0071S, Beyotime Biotechnology, China), following the manufacturer’s instructions. In brief, after treatment with siRNA, cells were incubated with 10 μM EdU for 2 h at 37 °C. After washing them with PBS, cells were fixed with 4% paraformaldehyde at RT for 15 min, then washed and permeabilized with 0.1% Triton X-100 for another 1 h. Cells were then incubated with Click Reaction Mixture for 30 min at RT and protected from light. For fluorescence microscopy, cells were incubated with DAPI for 5 min. For fluorescence-activated cell sorting (FACS) analysis, cells were washed twice in 1% BSA solution and incubated with propidium iodide (PI) and 20 µg/mL RNase A for 30 min in the dark. Samples were stored at 4 °C until analysis by BD FACSCelesta (BD Biosciences, San Jose, California, USA). Each experiment was conducted in three independent biological replicates. FACS data were processed by software FlowJo.

### Cell viability assay and DNA fiber assay using POLD3-knockdown human cells

The doxycycline (Dox)-inducible POLD3 knockdown HeLa cell line (shPOLD3-Hela) was cultured and treated with 1 μg/mL Dox to induce gene knockdown. Following induction, cell viability was assessed using the cell counting kit-8 (Supplementary Table 2) according to the manufacturer’s instructions. This experiment was performed with three biological replicates.

For DNA fiber assays, POLD3 knockdown was performed in 293 T cells using siRNA (5’-GAUGCUGUAUGAUUAUGUUTT-3’, siRNA1-hPOLD3; 5’-GGCCUCUGUUCAAUACUGATT-3’, siRNA2-hPOLD3; 5′-UUCUCCGAACGUGUCACGUTT-3′, siRNA-Ctrl; GenePharma, Shanghai, China). Knockdown efficiency was verified by western blot with anti-POLD3 antibody, with H3 as a loading control. After siRNA treatment, cells were labeled with 40 μM 5-Chloro-2-deoxyuridine (CldU) for 20–30 min, washed three times with pre-warmed PBS, and then labeled with 100 μM 5-Iodo-2-deoxyuridine (IdU) for 20–30 min. Labeled cells were trypsinized, resuspended in cold PBS, and mixed with unlabeled cells at a 1:2 ratio to a final concentration of 2.5 × 10⁵ cells/mL.

For DNA fiber spreading, 2.5 μL of the cell suspension was pipetted onto a clean glass slide for 3 min, followed by 7.5 μL lysis buffer (200 mM Tris-HCl pH 7.5, 50 mM EDTA, 0.5% SDS) for 5 min at room temperature. Slides were tilted ( ~ 15°) to spread DNA fibers and were air-dried in the dark. Samples were fixed in cold methanol:acetic acid (3:1, vol/vol) overnight at 4°C. DNA was denatured in HCl at 37 °C for 60–80 min, washed twice with PBS, and blocked with 2% BSA in washing buffer (0.1% Tween-20 in PBS) at room temperature for 20–40 min.

Slides were incubated with primary antibodies—mouse anti-IdU and rat anti-CldU—in 1% BSA buffer (60 μL/slide, 37 °C, 1.5 h). After washing, secondary antibodies were applied: donkey anti-mouse Alexa Fluor 594 and donkey anti-rat Alexa Fluor 488 for 45 min at 37°C or 1 h at room temperature. Slides were washed, air-dried, and mounted with antifade reagent. Detailed antibody information was listed in Supplementary Table 1.

DNA fibers were imaged using a fluorescence microscope equipped with a 60× to 100× oil immersion objective lens. Data are presented as mean ± SD from *n* = [200] fibers per condition. Statistical significance was determined using Mann–Whitney U test.

### eSPAN analysis of strand bias for WDHD1 and POLD3 during DNA replication

eSPAN was performed in wild-type or WDHD1 and POLD3 siRNA-knockdown mouse E14TG2a cells according to Zhang’s protocol [52]. Briefly, exponentially growing cells were treated with 50 μM BrdU (No. B5002, Sigma-Aldrich, St. Louis, MO, USA) for 30 min and harvested. Next, 1 × 10^6^ cells were incubated with 30 μL pre-washed concanavalin A-coated magnetic beads at RT for 20 min, after which primary antibodies (H3K36me3, H4K20me2, and H4K12ac) were incubated with cells in 200 μL antibody buffer overnight at 4 °C. To make secondary-antibody–pAG-Tn5 complex, secondary antibody [rabbit-anti mice IgG H&L (No. ab46540, Abcam, Cambridge, UK)] and pAG-Tn5 (No.M058-YH01, Novoprotein, China) were incubated for 1 h. After overnight at 4 °C, beads were washed once with Dig-wash buffer and incubated with 2 μg secondary-antibody–pAG-Tn5 complex in 200 μL Dig-300 buffer at RT for 1 h. Tagmentation was performed at 37 °C for 1 h. Reactions were stopped by adding 15 mM EDTA, 0.1% SDS, and 100 µg/mL proteinase K and incubating at 50 °C overnight. Detailed antibody information was listed in Supplementary Table 1.

Supernatants were purified using chromatin immunoprecipitation (ChIP) columns (No. D5205, Zymo Research, Irvine, CA, USA) and subjected to an oligo replacement reaction. Briefly, DNA products were mixed with 0.5 mM deoxynucleotide triphosphate mix, 0.5 μM mosaic end adapter B (ME-B1) and 1x ampligase buffer using an annealing program (50 °C, 1 min; 45 °C, 10 min; ramp to 37 °C at 0.1 °C/s and hold). The repairing reaction was performed by adding T4 DNA polymerase and ampligase at 37 °C for 1 h. The reaction products were boiled and immediately chilled on ice.

Samples were further diluted with 1 mL ice-cold BrdU IP buffer and mixed with 1 μL *E. coli* tRNA (Sigma-Aldrich, St. Louis, MO, USA, 10 mg/mL) and BrdU antibodies (No. 555627, BD Pharmingen, San Diego, CA, USA) at 4 °C for 2 h. Prewashed protein G beads (25 µL; No. 17061802, GE Healthcare, UK) were mixed with each sample and rotated at 4 °C for 1 h. After washing three times, beads were incubated with elution buffer [50 mM Tris-HCl (pH 8.0), 10 mM EDTA and 1% SDS] for 15 min at 65 °C. DNA was purified with the DNA Clean & Concentrator-5 Kit (Zymo Research, Irvine, CA, USA), and library PCR was performed with standard Illumina Nextera indexing primers. Libraries were sequenced using paired-end (2 × 150 bp) sequencing on the Illumina NovaSeq 6000.

To determine the relative levels of parental and newly synthesized histones on each DNA strand in knockdown versus WT cells, we applied the following procedure. eSPAN signals were separated into left and right regions relative to the replication origins. Each replication region was further divided into four quadrants: Watson strand on the left (WL) and Crick strand on the right (CR), corresponding to lagging strands; Crick strand on the left (CL) and Watson strand on the right (WR), corresponding to leading strands. Within each quadrant, eSPAN raw read counts were normalized to the total mapped reads and the associated BrdU signal, providing a measure of histone enrichment per unit of newly synthesized DNA. Finally, the normalized eSPAN values were used to calculate the relative enrichment of histones in knockdown cells compared to WT according to the references [7, 42].

### Immunoprecipitation in vivo and western blot to measure the association between POLD3, MCM2, and histone H3

#### Co-immunoprecipitation (co-IP) using epitope-tagged constructs

Wild-type 293 T cells, 293 T cells carrying the plasmids POLD3-HA, POLD3(1–144)-NLS-HA, POLD3(145–466)-NLS-HA, or Flag-H3.1, and 293 T MCM2-2A mutant cells carrying the plasmid POLD3-HA (see Plasmid construction section of the Methods) were collected, washed, and spined down at 900 rpm 4 °C for 5 min. Then we discarded the supernatant and resuspended the cells in 1 mL cold cell lysis buffer (HEPES-KOH, PH 7.4, 1 mM EDTA, 1x protease inhibitor, 10% glycerol, 200 mM NaCl, 0.5% NP-40, and 0.02 μM PMSF), after which we added 5 μL of DNase I (Supplementary Table 2) to each sample and rotated them for 30 min at 4 °C; The supernatant was transferred into a new tube, and protein concentration was determined using the BCA protein assay; We added 7.5 μL ethidium bromide (EB) to each sample and incubated the samples on ice for 30 min, prior to centrifugation. Supernatant was transferred to new tubes. Beads [anti-HA (No. 26181, ThermoFisher Scientific, Rockford, IL, USA) or anti-Flag M2 (No. A2220, Sigma-Aldrich, St. Louis, MO, USA)] were washed with 1 mL cold cell lysis buffer three times. We used 60 μL sample as input for the western blot, and the rest of the samples were incubated with 60 μL beads at 4 °C for 3 h. Beads were washed with 1 mL cold wash buffer six times at 4 °C. To elute the co-IP products, 1x elution buffer (10 mM Tris-HCl pH 8.0, 150 mM NaCl, 1% SDS, 10 mM EDTA, 1x Protease Inhibitor) were used. The co-IP results were evaluated using western blots.

HA-tagged immunoprecipitation in doxycycline-induced POLD3 knockdown HeLa cell lines overexpressing full-length or truncated POLD3 was performed as described above for HA-tagged immunoprecipitation in 293 T cells. All experiments were independently repeated two times.

#### Co-immunoprecipitation using MCM2 antibody

Wild-type 293 T cells or MCM2-2A mutant 293 T cells were collected and lysed in cell lysis buffer (50 mM HEPES pH 7.4, 200 mM NaCl, 10% glycerol, 1 mM EDTA, 0.5% NP-40, supplemented with protease inhibitors). Lysates were centrifuged at maximum speed for 15 min, and the supernatants were incubated overnight at 4 °C with 2–4 μg of the MCM2 antibodies or IgG (as a negative control). Protein A/G magnetic beads (Supplementary Table 2) were added and incubated for 1 h at 4 °C. The beads were then washed extensively with the washing buffer (50 mM HEPES pH 7.4, 100 mM NaCl, 10% glycerol, 1 mM EDTA, 0.05% NP-40, supplemented with protease inhibitors) and resuspended in protein loading buffer for boiling. Finally, the immunoprecipitated proteins were resolved and analyzed by western blots. All experiments were independently repeated two times. Detailed antibody information was listed in Supplementary Table 1.

### Purification of POLD3, POLA1, MCM2, (H3.1-H4)_2_ tetramer, and H2A-H2B dimer proteins for pulldown assays

POLD3, truncated POLA1 (1 to 200 amino acids), MCM2, MCM2-HBD, and MCM2-2A-HBD proteins were expressed in BL21 *E. coli* cells (ToloBio, Wuxi, China) under standard conditions. Briefly, the expression plasmids were transferred into *E. coli* BL21 cells, which were then cultured in 200 mL of LB medium at 37 °C with shaking (200 rpm) until the OD600 reached 0.6. Protein expression was induced by adding IPTG to a final concentration of 1.2 mM, and the incubation temperature was lowered to 16°C for 14–16 h. Afterward, cells were harvested by centrifugation at 8,000 rpm for 10 minutes. POLD3, truncated POLA1, MCM2, MCM2-HBD, and MCM2-2A-HBD were purified using Ni-NTA affinity columns.

Histone plasmids (H3.1-H4 or H2A-H2B; a gift from Southern University of Science and Technology, China) were also transferred into *E. coli* BL21 cells. A single clone was cultured at 37 °C until an OD_600_ value of 2 was achieved, at which point 0.5 mM IPTG was added, and the culture was shaken for 5 h. After protein expression, the bacteria were centrifuged at 8000 rpm for 10 min and collected. Collected bacteria were resuspended in 20 mM Tris-HCl, pH 7.5 with 1 M NaCl and broken using ultrasound plus. Supernatant proteins were further purified with heparin and linearly eluted with 500 mM–2 M NaCl buffer. The purified components were collected and subjected to a second round of purification on a HiLoad 16/600 Superdex 200 column. Protein concentrations were calculated with the Pierce BCA Protein Assay Kit (ThermoFisher Scientific, Rockford, IL, USA).

### Immunoprecipitation in vitro to measure the interaction between POLD3, MCM2, H2A-H2B dimer and the (H3.1-H4)_2_ tetramers

To verify the interaction between POLD3 and MCM2, 50 μL of Glutathione Sepharose 4B beads (Supplementary Table 2) were suspended in 200 μL binding buffer [20 mM Tris-HCl (pH 7.5), 500 mM NaCl] and incubated with 1.0 nmol of GST-tagged MCM2 at 4 °C for 30 min. Subsequently, 1.5 nmol of POLD3 was added, and the mixture was incubated at 4 °C for an additional 2 h. The beads were washed four times with 1 mL of washing buffer [20 mM Tris-HCl (pH 7.5), 500 mM NaCl, 0.5% (v/v) Triton X-100]. Finally, 50 μL of SDS-PAGE loading buffer was added for analysis by 4–20% reducing SDS-PAGE.

To assess the interaction between POLD3 and (H3.1-H4)_2_ tetramers or H2A-H2B dimer, 50 μL of Strep-Tactin XT Sepharose beads (No. 29401324, Cytiva, Marlborough, MA, USA) were suspended in 200 μL binding buffer [20 mM Tris-HCl (pH 8.0), varying NaCl concentrations (200, 300, or 500 mM), 1 mM EDTA (pH 8.0), 5% glycerol, and 0.2% Triton X-100, with 1x protease inhibitor]. The beads were incubated with 1.5 nmol of Strep II-tagged POLD3 or truncated POLA1 (as a positive control for (H3-H4)_2_ tetramer binding) at 4 °C for 30 min. Then, 1.5 nmol of (H3.1-H4)_2_ tetramers or H2A-H2B dimer (1:1 molar ratio) were added and incubated at 4 °C for another 2 h. The Sepharose beads were washed four times (5–10 min each time) with 1 mL washing buffer [20 mM Tris-HCl (pH 8.0), 1 M NaCl, 5% glycerol, and 0.5% Triton X-100] and then incubated with 40 μL elution buffer [50 mM biotin, 100 mM Tris-HCl (pH 8.0), 150 mM NaCl, and 1 mM EDTA (pH 8.0)] on a rotation wheel at 4 °C for 30 min. The supernatant solution was collected. All samples were analyzed by 4–20% reducing SDS-PAGE.

To verify the complex formation of POLD3, MCM2, and histones, we suspended 50 μL Strep-Tactin XT Sepharose beads with 200 μL binding buffer [20 mM Tris-HCl (pH 8.0), 300 mM NaCl concentrations, 1 mM EDTA (pH 8.0), 5% glycerol, and 0.2% Triton X-100, supplemented with 1x protease inhibitor]. The beads were then incubated with 1.5 nmol of Strep II-tagged POLD3 at 4 °C for 30 min. Subsequently, 1.5 nmol of (H3.1-H4)_2_ tetramers were added to the mixture and incubated at 4 °C for 2 h. The beads were then washed three times with the washing buffer, after which the beads were incubated with 1.5 nmol MCM2-His at 4 °C for another 2 h. After washing three times, proteins were eluted with elution buffer at RT for 20 min. All samples were analyzed by 4–20% reducing SDS-PAGE.

The immunoprecipitation experiments were independently repeated three times.

### Biolayer interferometry (BLI) assays to measure binding between POLD3, MCM2, and the (H3.1-H4)_2_ tetramers

Real-time binding assays of POLD3 with MCM2, MCM2-HBD, MCM2-2A-HBD, or (H3.1-H4)_2_ tetramers were conducted using the BLItz® system (Sartorius) in a binding buffer (1x PBS and 0.02% Tween). Purified POLD3 was immobilized onto Dip and Read High Precision Streptavidin (SAX) Biosensors (Sartorius). To test the binding affinity of POLD3 with (H3.1-H4)_2_, the binding kinetics were assessed across a range of purified (H3.1-H4)_2_ tetramer concentrations. The experiments for POLD3 binding (H3.1-H4)_2_ tetramers employed the following advanced kinetics settings: (1) initial baseline measurement: buffer only, measured for 60 s; (2) loading phase: immobilization of POLD3 onto sensors for 300 s; (3) effective baseline measurement: buffer only, measured for 120 s; (4) association phase: interaction of POLD3 with (H3.1-H4)_2_ tetramers for 180 s; (5) dissociation phase: monitoring the dissociation of (H3.1-H4)_2_ tetramers from POLD3 for 226 s. To test the effect of histone binding domain from MCM2 on the interaction of MCM2 and POLD3, we tested the binding affinity of POLD3 with MCM2-HBD or MCM2-2A-HBD. The experiments employed the following advanced kinetics settings: (1) initial baseline measurement: buffer only, measured for 60 s; (2) loading phase: immobilization of POLD3 onto sensors for 300 s; (3) effective baseline measurement: buffer only, measured for 120 s; (4) association phase: interaction of POLD3 with MCM2, MCM2-HBD or MCM2-2A-HBD for 300 s; (5) dissociation phase: monitoring the dissociation of MCM2, MCM2-HBD or MCM2-2A-HBD from POLD3 for 600 s.

Real-time binding assays to explore the interactions between MCM2 and either POLD3 or (H3.1-H4)_2_ tetramers were conducted using the BLItz® system (Sartorius) with a binding buffer (1x PBS and 0.02% Tween). MCM2 was immobilized on Dip and Read High Precision Anti-GST Biosensors (Sartorius). Binding kinetics were measured across a range of POLD3 or (H3.1-H4)_2_ tetramer concentrations under advanced kinetics settings, as outlined below: (1) initial baseline measurement: buffer only, measured for 120 s; (2) loading of MCM2: immobilization of MCM2 onto the biosensors for 300 s; (3) effective baseline measurement: buffer only, measured for 120 s; (4) association phase: interaction between MCM2 and POLD3 or (H3.1-H4)_2_ tetramers recorded for 180 s; (5) dissociation phase: monitoring the dissociation of (H3.1-H4)_2_ tetramers from MCM2 for 300 s and monitoring the dissociation of POLD3 from MCM2 for 260 s.

Real-time binding assays were performed to study the interactions between POLD3, MCM2, and (H3.1-H4)_2_ tetramers using the BLItz® system (Sartorius) with a binding buffer (1x PBS and 0.1% Tween). POLD3 was immobilized onto Dip and Read High Precision Streptavidin (SAX) Biosensors (Sartorius). Following immobilization, MCM2 was added, and binding kinetics were measured using advanced kinetics settings as described below: (1) initial baseline measurement: buffer only, measured for 120 s; (2) loading of POLD3 onto sensors for 300 s; (3) effective baseline measurement: buffer only, measured for 120 s; (4) association of POLD3 and MCM2: interaction measured for 300 s. Next, (H3.1-H4)_2_ tetramers were added, and the following advanced kinetics settings were applied: (1) association of POLD3, MCM2 and (H3.1-H4)_2_ tetramers: measured for 300 s; (2) dissociation of POLD3, MCM2 and (H3.1-H4)_2_ tetramers: measured for 600 s.

All data were normalized to the effective baseline signal and analyzed using Octet Analysis Studio software for quantitative assessment of binding kinetics. All BLI experiments were independently repeated twice.

## Supplementary information

Supplementary Figure, Supplementary Table, and Text

Original Western blot of the Figures

## Data Availability

Data supporting the findings of this study are available in the article and its supplementary figures and tables. In addition, data reported in this paper can be also shared by the lead contact, HaiyunGan, PhD (hy.gan@siat.ac.cn) upon request. Further information and requests for resources and reagents should be directed to and will be fulfilled by lead contact. Plasmids used in this study are available upon request from the lead contact.

## Code availability

All MS proteomics data are available from the PRoteomics IDEntifications database (PRIDE: PXD048030), publicly accessible at http://proteomecentral.proteomexchange.org. Username: reviewer_pxd048030@ebi.ac.uk. Password: 0Xnyh3Fg. This paper does not report on the original code. Analysis of flow cytometry data was performed using BD FACSDiva (Version 8.0.1.1) and FlowJo (v10.8.1) software package. Raw MS data were analyzed with MaxQuant software, version 1.6.10.43. The network analysis of candidate proteins was visualized using Cytoscape v.3.8.0. Gene Ontology (GO) enrichment analysis for enriched proteins was performed using Metascape (https://metascape.org). The quantification analysis of Western blot band intensity was determined with ImageJ (v150) software. The eSPAN anaylsis was implemented as described in the publication, Watson and Crick strands were merged and partition or RFD was calculated using public R scripts [52]. All BLI data were analyzed using Octet Analysis Studio software. Data visualization to generate the PCA plots, heatmaps, and volcano plots was carried out using the following R packages: ggplot2 (v3.4.2), pheatmap (v1.0.12), and EnhancedVolcano (v1.20.0).
