# Supplementary material for: Proximal proteomics analysis reveals DNA polymerase δ subunit 3 is a new MCM2 binding partner and promotes parental histones inheritance in mammalian cells
Source: Cell Death Differ. 2025 Nov 27;33(5):1050–64. doi: 10.1038/s41418-025-01619-z (PMC13156308; doi:10.1038/s41418-025-01619-z)
Supplement: Supplementary file 1 — Supplementary Figure, Supplementary Table, and Text [file 41418_2025_1619_MOESM1_ESM.pdf]

Supplementary Materials for

Proximal proteomics analysis reveals DNA polymerase δ subunit 3 is a new MCM2 binding partner and promotes parental histones inheritance in mammalian cells.

Yaping Sun[1, 2,#], Xiaoyan Liang[1,#], Fang Liu[3,#], Wenjuan Zhao[4,12#], Jiaqi Zhou[1], Yue Li[5], Yuan Yao[1], Ziwei Zhang[1], Gang Li[6], Kuiming Chan[7], Daoqin Zhang[8], Zhiquan Wang[9], Yuan Gao[10], Chuanhe Yu[11], Yuchun Wu[1], Xing Kang[1], Lingyu Qiu[1], Nan Li[3,4,*], and Haiyun Gan[1,*]

Supplementary Figures:

Fig.S1. Schematic of proteomics for MCM2-TurboID and MCM2-2A TurboID proximity labeling.

Fig.S2. Optimization of TurboID proximity labeling process by testing different fetal bovine serum (FBS) and various labelling times.

Fig.S3. Western blots showing biotinylated proteins (streptavidin-HRP) after successful TurboID proximity labeling.

Fig.S4. Volcano plots depict proteins enriched through TurboID proximity labeling in mouse NIH3T3 cells for POLE3 and MCM2.

Fig.S5. GO term analysis of proteins closely associated with histone chaperones enriched in MCM2-TurboID and POLE3/POLE4-TurboID proteomic data.

Fig.S6. MCM2 immunoprecipitation reveals that its interaction with BRD4 or SSRP1 depends on its histone-binding activity.

Fig.S7. Normalized enrichment intensity of histone H3 and H4 proteins in TurboID proximity labeling proteomics for MCM2 and MCM2-2A in NIH3T3 cells.

Fig.S8. Validation of WDHD1 knockdown and eSPAN analysis of histone distribution bias in mouse ES cells.

Fig.S9. Knockdown of WDHD1 or POLD3 with siRNA does not affect the cell cycle for mouse E14TG2a embryonic stem cells.

Fig.S10. Effects of POLD3 knockdown on cell viability and replication fork progression.

Fig.S11. Doxycycline-inducible knockdown of endogenous POLD3 in Hela cells expressing full-length or truncated POLD3 and their interaction with histone H3.

Fig.S12. Purified strep-tagged POLA1 interacts with purified $(H3\text{-}H4)_2$ tetramers outside of the cells.

Fig.S13. A biolayer interferometry (BLI) assay using immobilized POLD3 shows that the interaction of POLD3 and MCM2 occurs independent of MCM2's histone-binding domain.

Fig.S14. Endogenous MCM2 co-immunoprecipitated with POLD3 and histone H3 in 293T cells.

Fig.S15. The correlation analysis of eSPAN revealed that the parental histone distribution process of MCM2 has a close relationship with that of POLD3.

Fig.S16. Expression analysis of endogenous and overexpressed MCM2, POLE3, and POLE4 proteins.

Fig.S17. Successful synchronization of wild-type and bait-TurboID-tagged NIH3T3 cells in S phase, using a double thymidine block treatment.

Table.S1. Antibody information used in this study.

Table.S2. Commercial reagents and materials.

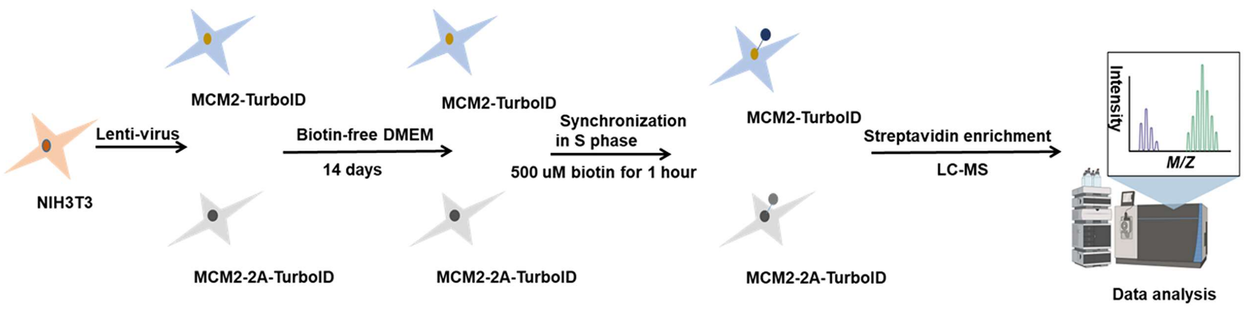

Supplementary Figure 1: Schematic of proteomics for MCM2-TurboID and MCM2-2A TurboID proximity labeling.

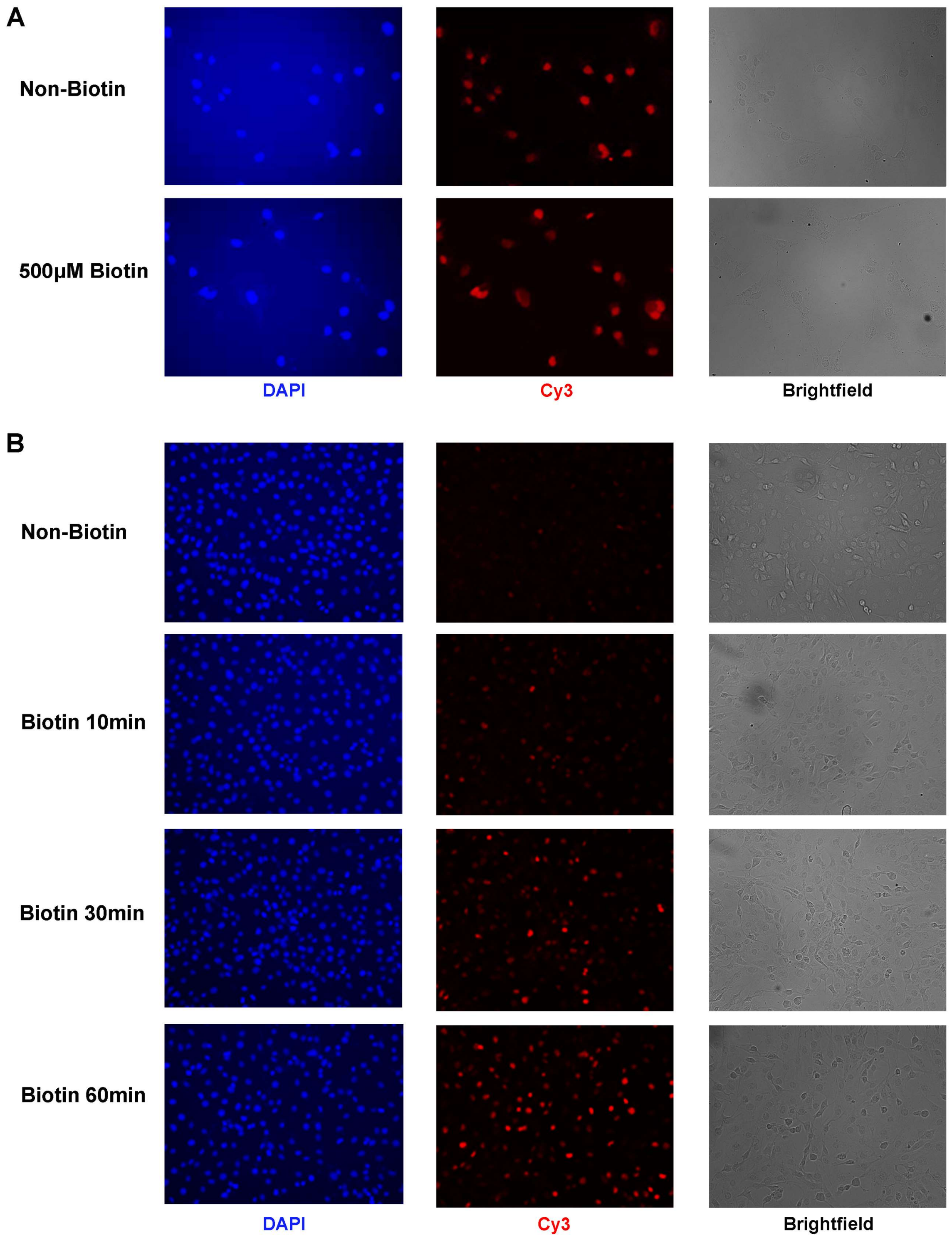

Supplementary Figure 2: Optimization of TurboID proximity labeling process by testing different fetal bovine serum (FBS) and various labelling times.

A and B. Representative immunoflurescence images show biotinylated proteins (streptavidin-Cy3) and DAPI-stained nucleus in mouse NIH3T3 cells transfected with MCM2-TurboID-HA expression plasmids and cultured in DMEM medium containing (A) normal or (B) dialyzed FBS. A. Even if no extra biotin were added, normal FBS with biotin caused lots of non-specific background labeling in TurboID-bait overexpressing cells. B. Dialyzed FBS was used to reduce the non-specific background labeling in TurboID-bait overexpressing cells. Incubation with biotin for 1 h was enough to perform proximity labeling.

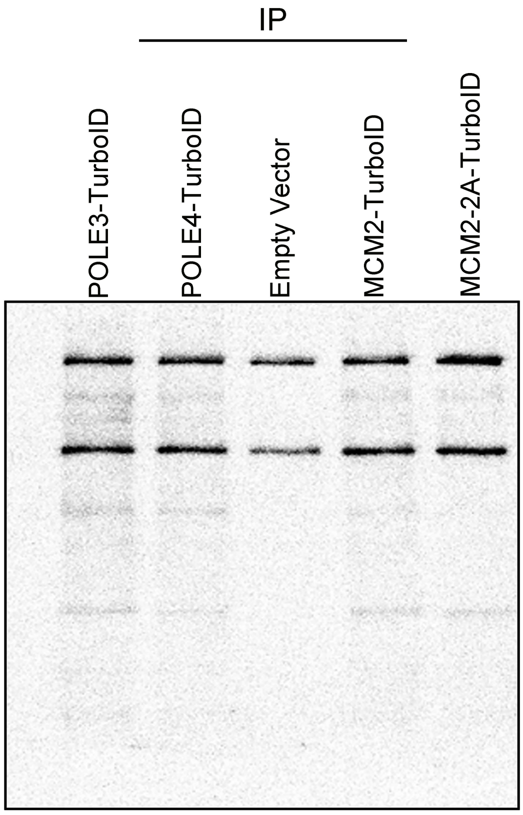

Supplementary Figure 3: Western blots showing biotinylated proteins (streptavidin-HRP) after successful TurboID proximity labeling.

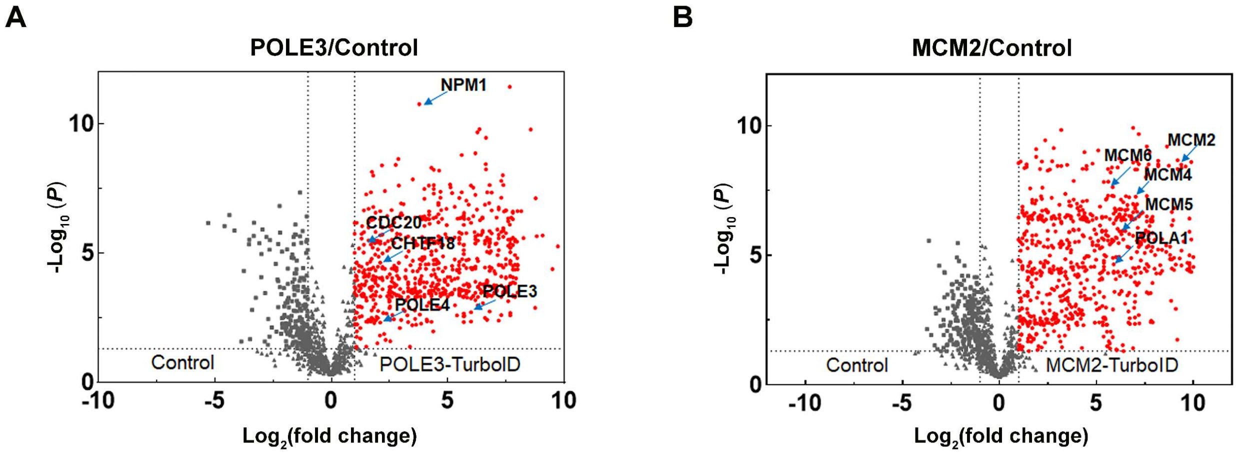

Supplementary Figure 4: Volcano plots depict proteins enriched through TurboID proximity labeling in mouse NIH3T3 cells for POLE3 and MCM2.

A and B. The plots display statistically significant proteins based on fold-change enrichment and p-values, highlighting the interactome data for each bait protein. The gray dotted threshold lines indicate a P-value of 0.05 (horizontal) and a $\log_2$ (fold change) of -1 or $+1$ (vertical). Proteins significantly enriched in the POLE3-TurboID (A) and MCM2-TurboID (B) proteomics are marked with red dots.

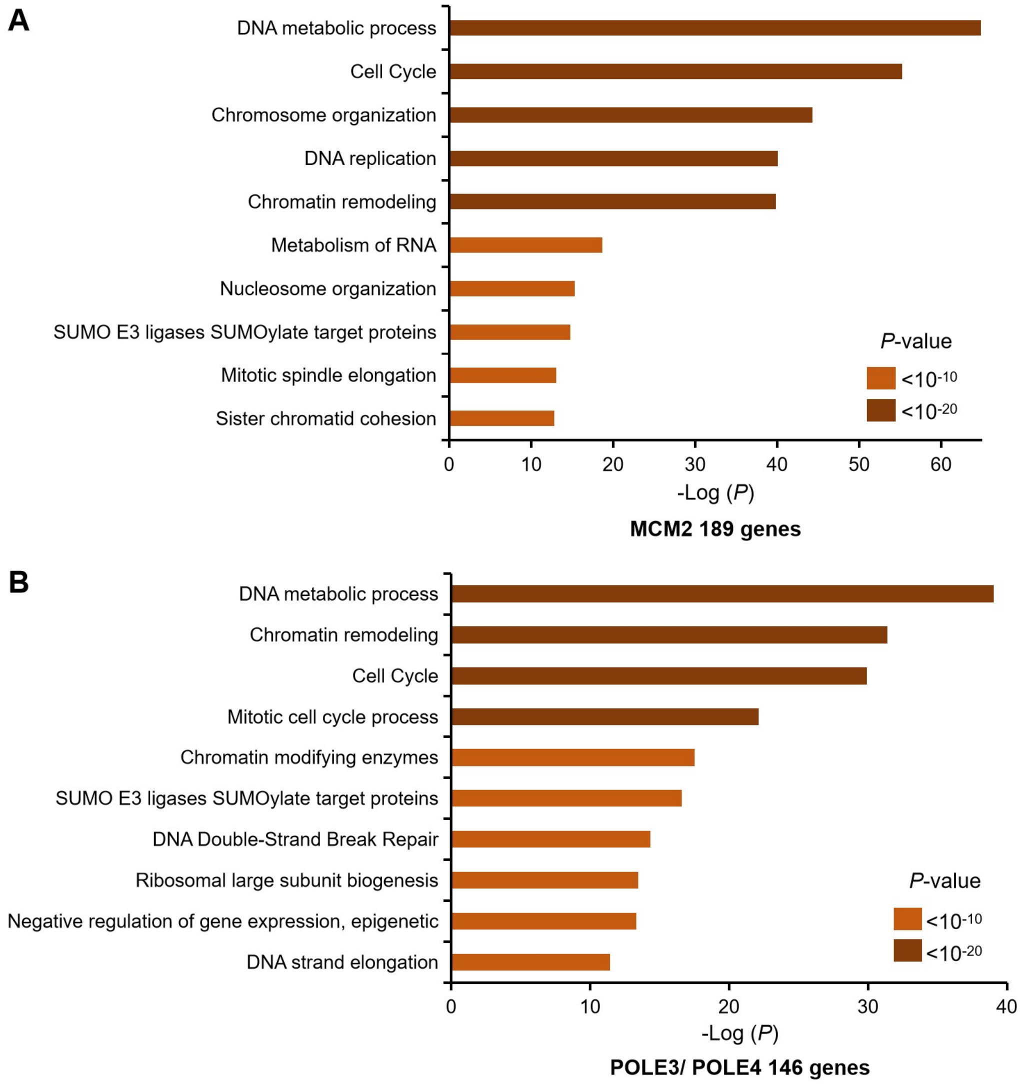

Supplementary Figure 5: GO term analysis of proteins closely associated with histone chaperones enriched in MCM2-TurboID and POLE3/POLE4-TurboID proteomic data.

A. GO term analysis of proteins enriched in MCM2-TurboID proximity labeling. B. GO term analysis of proteins enriched in POLE3/POLE4-TurboID proximity labeling. These analyses highlight the functional categories of proteins closely linked to histone chaperones, underscoring the robustness of the identified histone chaperone candidate library.

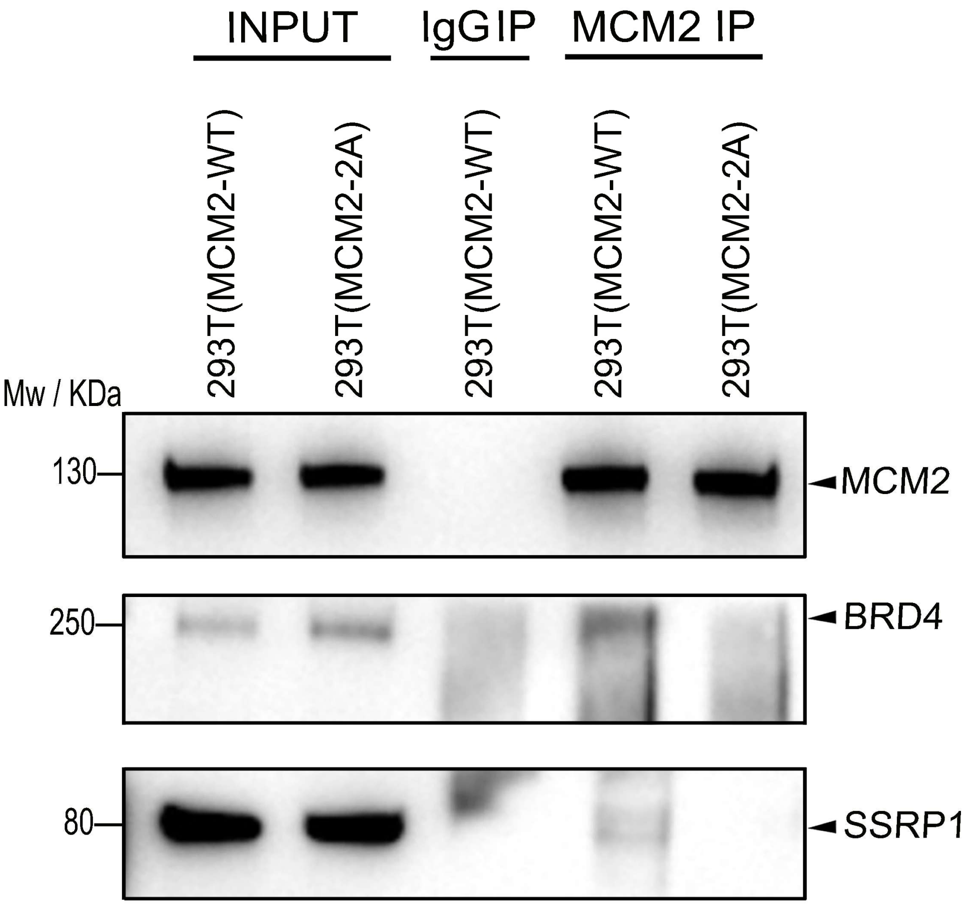

Supplementary Figure 6: MCM2 immunoprecipitation reveals that its interaction with BRD4 or SSRP1 depends on its histone-binding activity.

Immunoprecipitation (IP) was performed using an anti-MCM2 antibody in wild-type and MCM2-2A mutant 293T cells. In wild-type cells, MCM2 successfully co-immunoprecipitated BRD4 and SSRP1. However, in MCM2-2A mutant cells, which lack histone-binding activity, these interactions were lost, suggesting that the association of MCM2 with BRD4 and SSRP1 was dependent on its histone-binding domain. The IgG IP group served as the negative control, in which IgG was used for immunoprecipitation instead of the anti-MCM2 antibody.

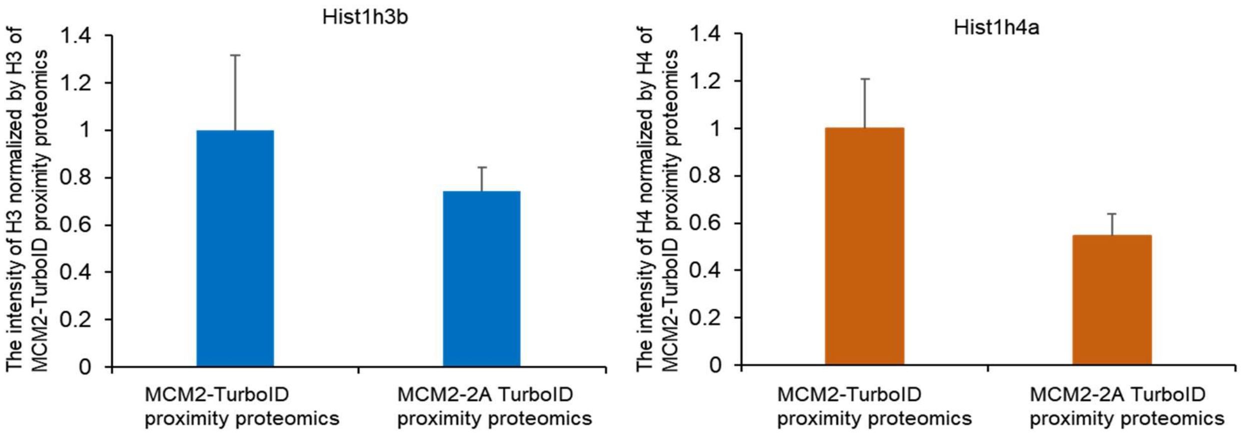

Supplementary Figure 7: Normalized enrichment intensity of histone H3 and H4 proteins in TurboID proximity labeling proteomics for MCM2 and MCM2-2A in NIH3T3 cells.

The intensity of histone H3 or H4 was normalized to the levels observed in MCM2-TurboID proximity labeling proteomics. Data are shown as mean ± SD (standard deviation).

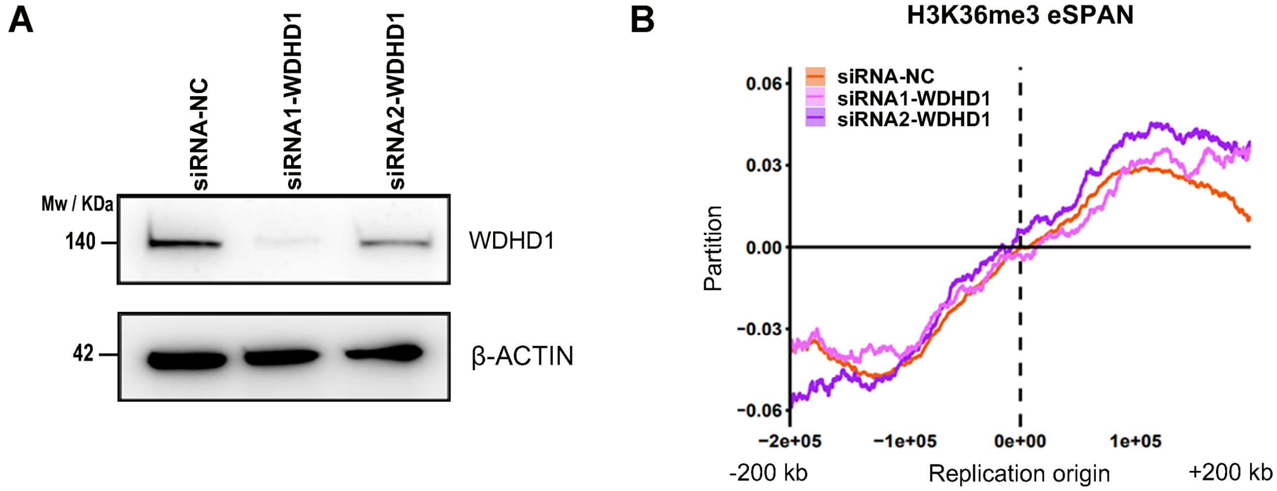

Supplementary Figure 8: Validation of WDHD1 knockdown and eSPAN analysis of histone distribution bias in mouse ES cells.

A. Western blot confirmed the successful knockdown of WDHD1 expression in mouse embryonic stem cells E14TG2a using small interfering (si)RNA. siRNA-NC represents cells transfected with a negative control siRNA. B. Average bias of H3K36me3 eSPAN results at selected initiation zones in wildtype (WT) and WDHD1-knockdown mouse E14TG2a cells, each with two repeats shown.

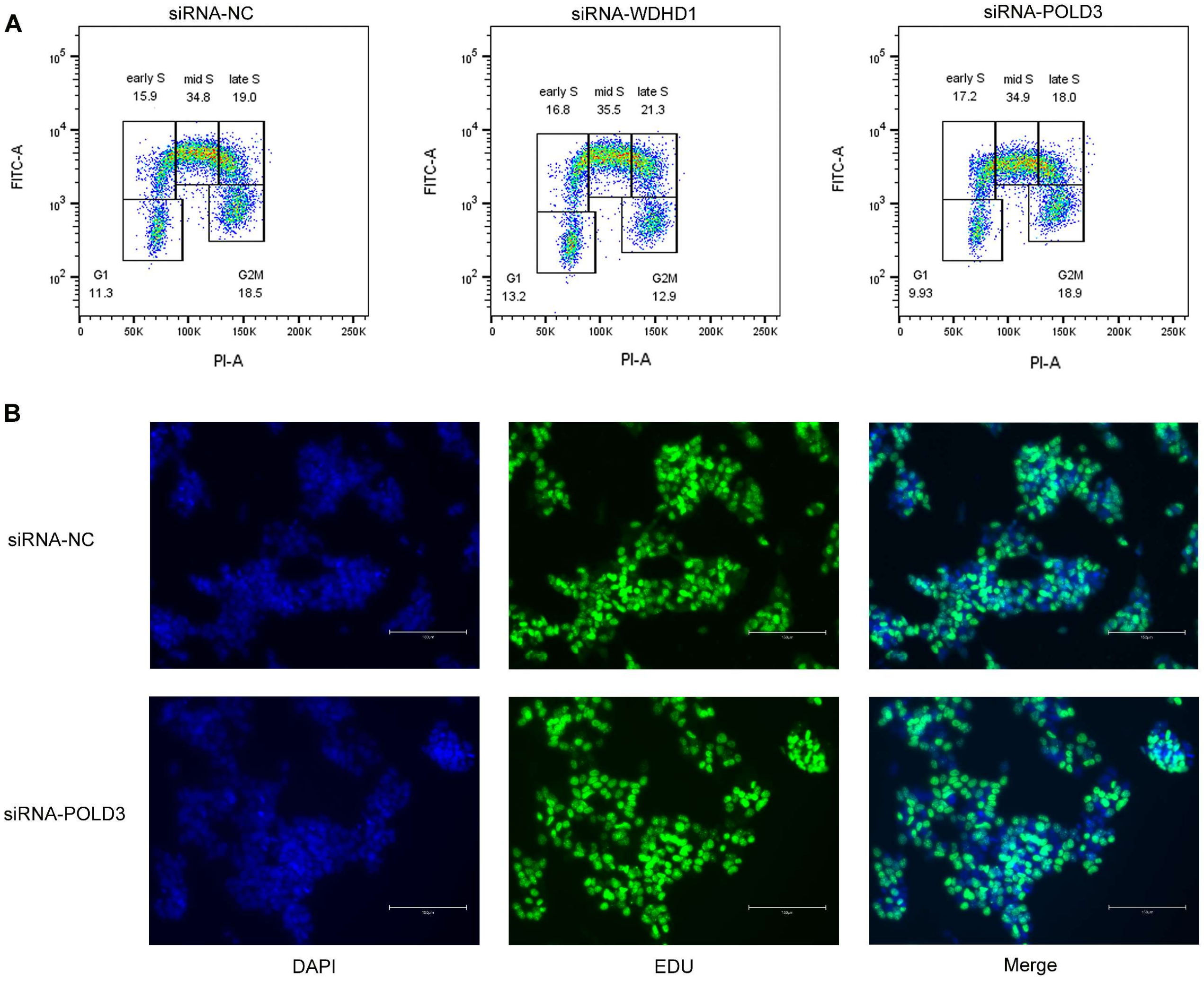

Supplementary Figure 9: Knockdown of WDHD1 or POLD3 with siRNA does not affect the cell cycle for mouse E14TG2a embryonic stem cells.

A. Cell cycle analysis of wildtype with negative control siRNA (*left*), WDHD1-knockdown (*middle*) and POLD3-knockdown (*right*) cells. Scatterplot of FITC fluorescence intensities (Y-axis) versus propidium iodide (PI) intensities (X-axis) are shown for a representative experiment out of three biological replicates. The results indicate no significant differences in cell cycle progression between groups. B. Representative immunofluorescent images show the knockdown of POLD3 has no effect on the cell cycle. After POLD3 knockdown with siRNA, cells were stained with DAPI, a nucleic-acid specific fluorophore and labeled with EDU to detect newly synthesized DNA. DAPI and EDU labeling patterns are indistinguishable between wild-type and POLD3-knockdown cells, further supporting the lack of cell cycle effects.

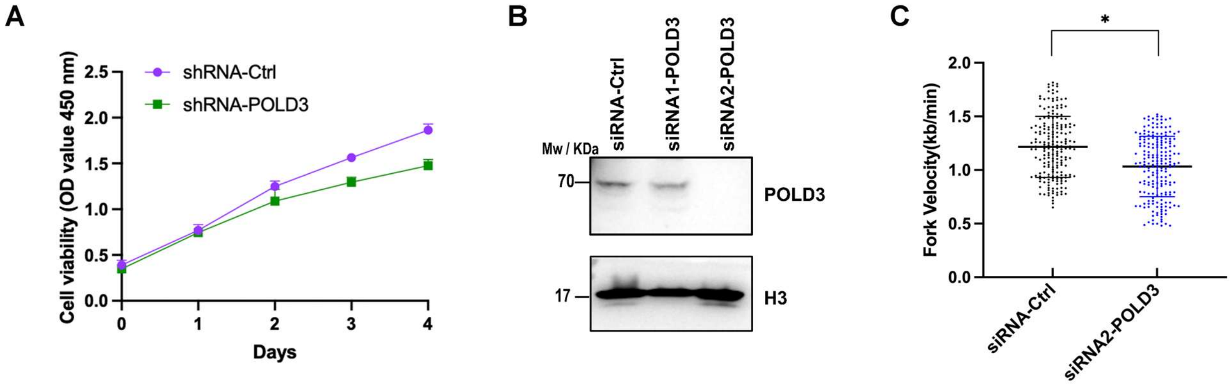

Supplementary Figure 10: Effects of POLD3 knockdown on cell viability and replication fork progression.
A. Cell viability of the doxycycline-inducible POLD3 knockdown Hela cells (shPOLD3-Hela) was measured using the cell counting kit-8 assay. Upon doxycycline induction of shRNA-mediated POLD3 knockdown for 48 hours, cell viability was comparable to that of the non-knockdown control group. B. Western blot confirmed the successful knockdown of POLD3 expression in 293T cells using siRNA. siRNA-Ctrl represented cells transfected with a negative control siRNA. C. Quantification of replication fork speed in wild-type and POLD3-knockdown 293T cells. Fork speed was slightly reduced from an average of ~1.25 kb/min in control cells to ~1.0 kb/min in POLD3-depleted cells (48 h post-transfection). Data are presented as mean ± SD from n = [200] fibers per condition. Statistical significance was determined using Mann–Whitney U test; *$p < 0.05$.

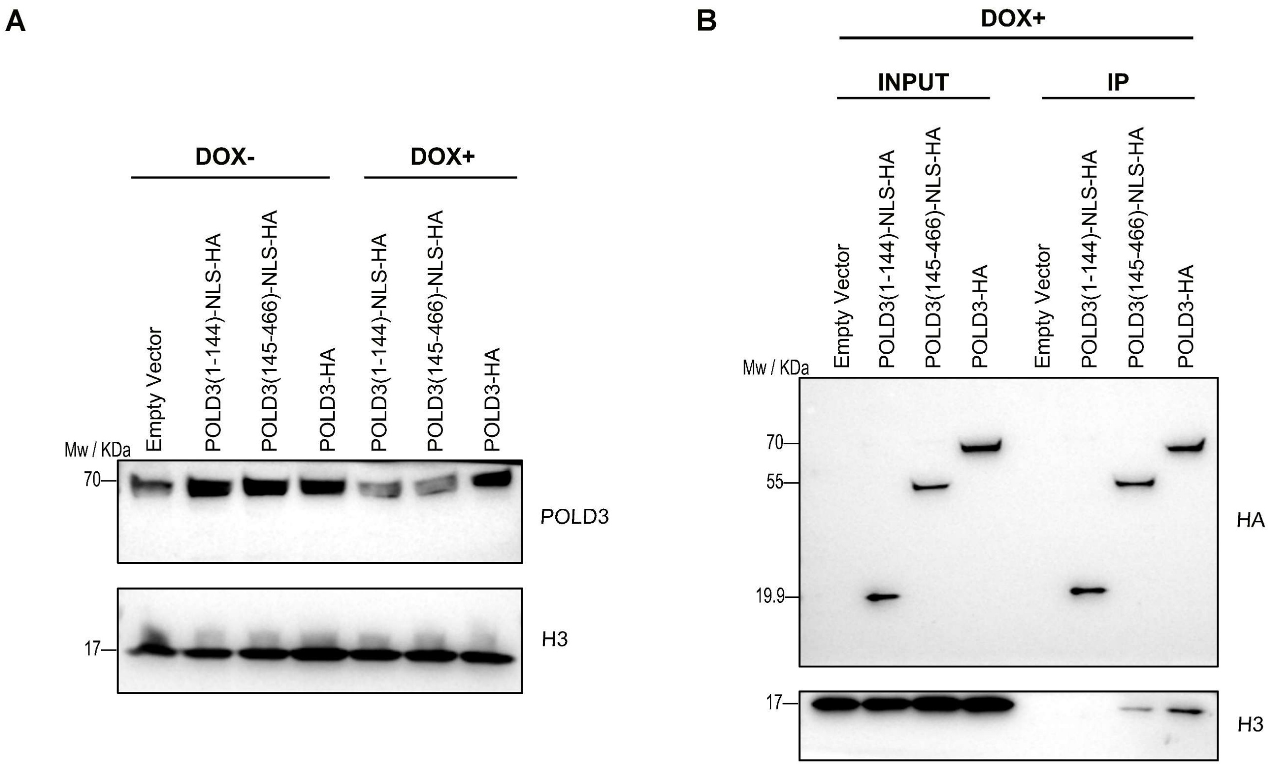

Supplementary Figure 11: Doxycycline-inducible knockdown of endogenous POLD3 in Hela cells expressing full-length or truncated POLD3 and their interaction with histone H3.

A. Validation of doxycycline-inducible knockdown of endogenous POLD3 in Hela cell lines expressing full-length or truncated POLD3 constructs. After 48 hours of Dox treatment, western blotting revealed a marked reduction of endogenous POLD3 in POLD3(1-144)- shPOLD3-Hela and POLD3(145-466)- shPOLD3-Hela cells, whereas total POLD3 levels in full length POLD3-HA-shPOLD3-Hela cells appeared largely unchanged due to overexpression of the exogenous POLD3-HA protein. This indicated effective knockdown of endogenous POLD3 despite the continued expression of the exogenous fusion protein. B. Under Dox-induced knockdown of endogenous POLD3, co-immunoprecipitation assays using HA-tags demonstrated that the C-terminal fragment POLD3(145–466), like the full-length protein, retained the ability to interact with histone H3, whereas the N-terminal fragment POLD3(1–144) did not. The immunoprecipitation was performed using an anti-HA antibody against HA-tagged proteins.

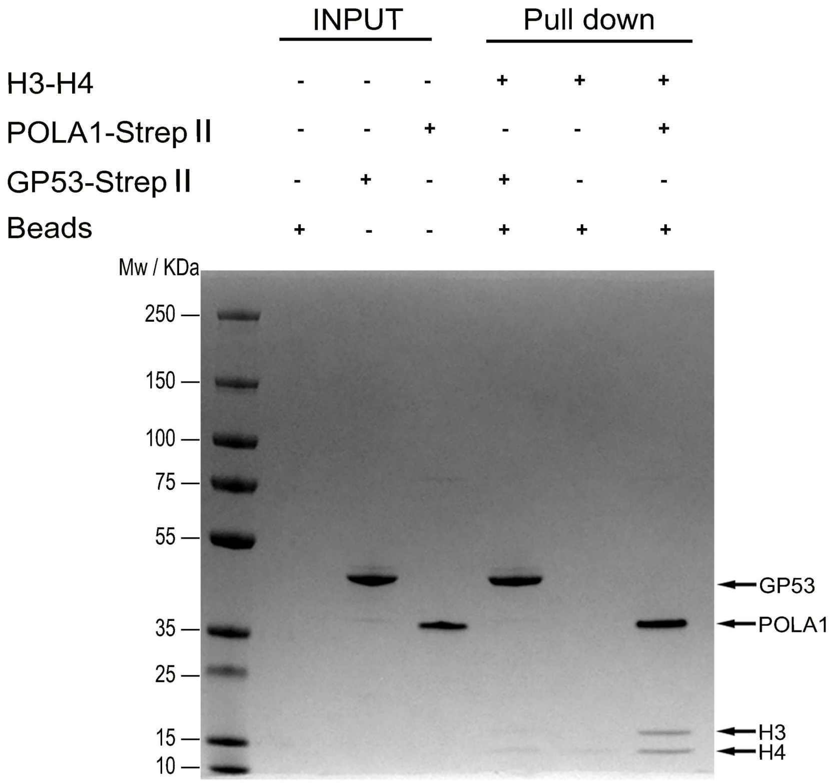

Supplementary Figure 12: Purified strep-tagged POLA1 interacts with purified $(H3\text{-}H4)_2$ tetramers outside of the cells. Gp53-strep as a negative control. Proteins and histones were stained with Coomassie Brilliant Blue (CBB).

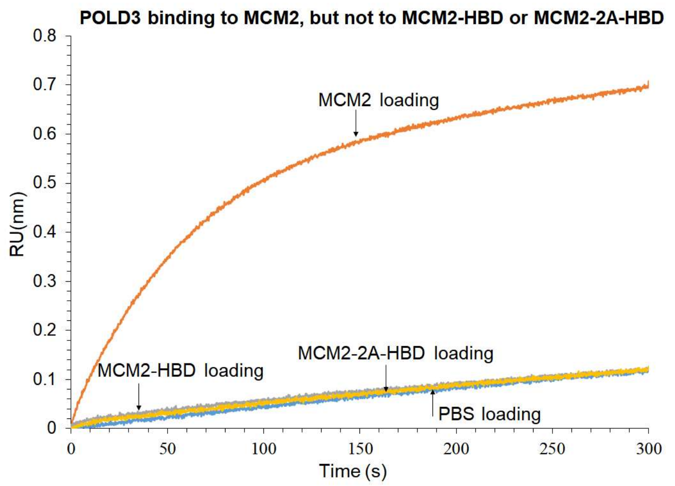

Supplementary Figure 13: A biolayer interferometry (BLI) assay using immobilized POLD3 shows that the interaction of POLD3 and MCM2 occurs independent of MCM2's histone-binding domain.

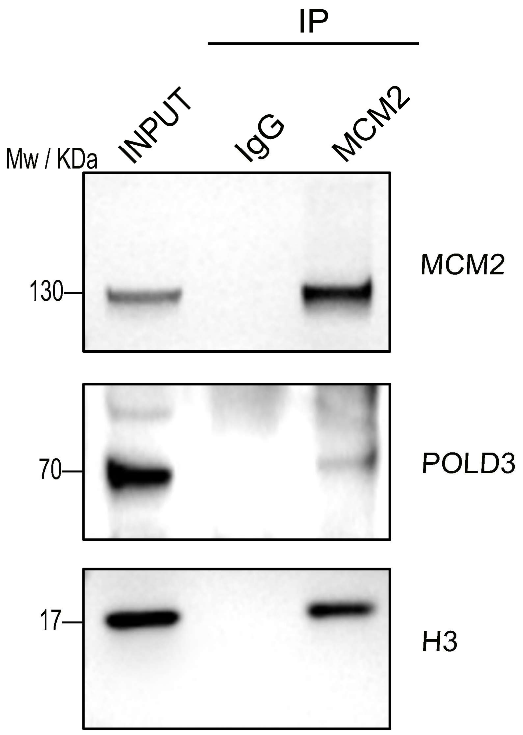

Supplementary Figure 14: Endogenous MCM2 co-immunoprecipitated with POLD3 and histone H3 in wild type 293T cells.

Immunoprecipitation (IP) was performed in wild type 293T cells using an anti-MCM2 antibody. The IgG group served as the negative control, in which IgG was used for immunoprecipitation instead of the anti-MCM2 antibody.

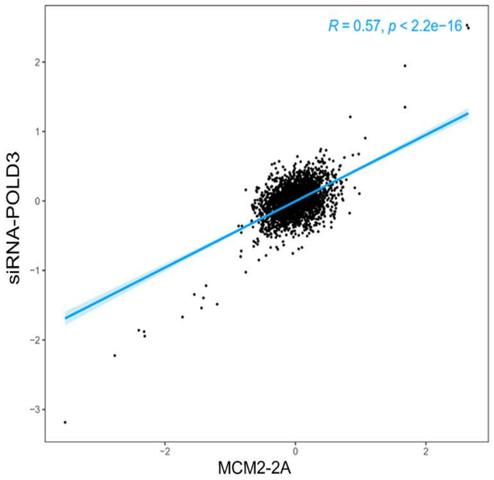

Supplementary Figure 15: The correlation analysis of eSPAN revealed that the parental histone distribution process of MCM2 has a close relationship with that of POLD3.

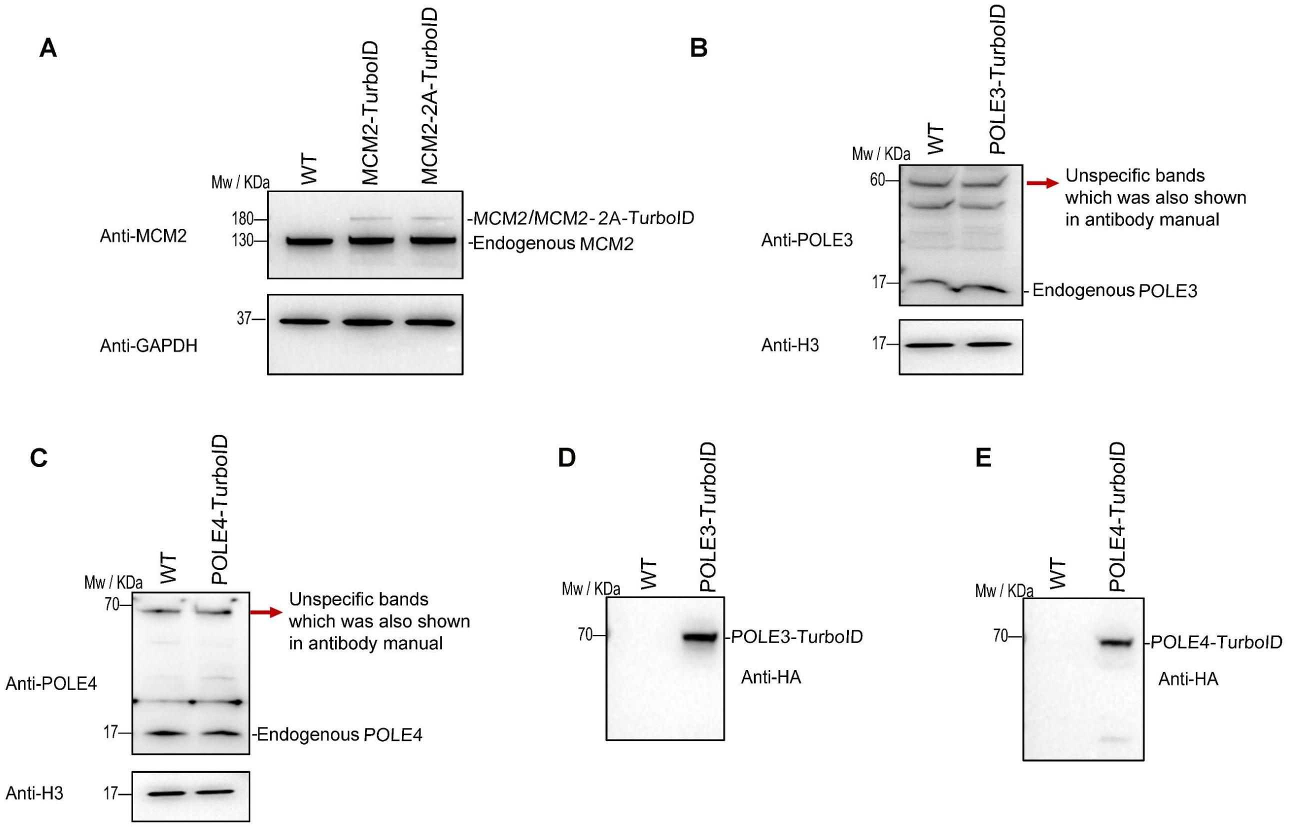

Supplementary Figure 16: Expression analysis of endogenous and overexpressed MCM2, POLE3, and POLE4 proteins.

A. Western blots using an anti-MCM2 antibody showed that endogenous MCM2 was expressed at much higher levels than MCM2-TurboID/MCM2-2A-TurboID fusion proteins in cells transfected with empty vectors or overexpression constructs. WT, MCM2-TurboID, and MCM2-2A-TurboID referred to wildtype cells transfected with empty vectors, MCM2-TurboID-NIH3T3 and MCM2-2A-TurboID-NIH3T3 cells, respectively. GAPDH was used as a loading control to confirm the equal protein loading. B and C. Western blots with commercial antibodies against POLE3 and POLE4[1, 2], detected only endogenous POLE3 and POLE4, along with nonspecific bands consistent with antibody datasheets, but did not identify overexpressing fusion proteins in cells transfected with empty vectors or overexpression constructs. WT, POLE3-TurboID, and POLE4-TurboID referred to wildtype cells transfected with empty vectors, POLE3-TurboID-NIH3T3 and POLE4-TurboID-NIH3T3 cells, respectively. H3 was used as a loading control to confirm the equal protein loading. D and E. Western blots with an anti-HA antibody, detected HA-tagged POLE3-TurboID and HA-tagged POLE4-TurboID, confirming successful overexpression in POLE3-TurboID-NIH3T3 and POLE4-TurboID-NIH3T3 cells. Collectively, these results (B-E) demonstrate that the expression levels of the POLE3-TurboID and POLE4-TurboID fusion proteins were lower than those of their endogenous counterparts.

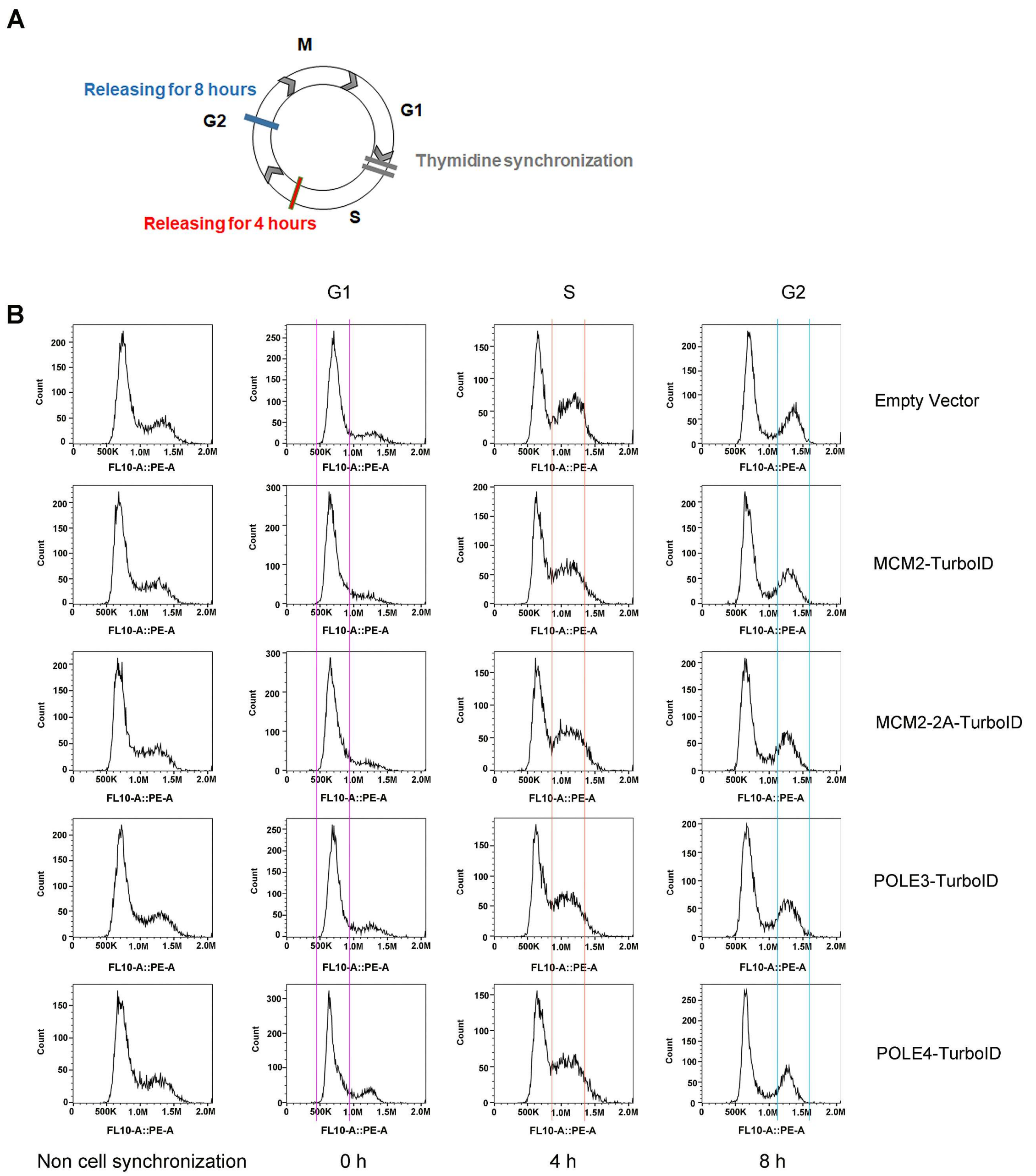

Supplementary Figure 17: Successful synchronization of wild-type and bait-TurboID-tagged NIH3T3 cells in S phase, using a double thymidine block treatment.

A. Schematic representation of cell cycle synchronization method. B. Flow cytometry analysis results of cells released from G1 phase at different time points showed effective synchronization. After double-treated thymidine, cells were released at different time points (0, 4, and 8 h), then trypsinized, fixed and stained with PI. The DNA content of each cell population was measured by flow cytometry.

Supplementary Table 1: Antibody information used in this study.

Antibodies	SOURCE	IDENTIFIER
Flag	Proteintech	Cat#66008-4-Ig
H3	Abcam	Cat#ab1791
H2A	Cell signaling technology	Cat#2718S
H2B	Abclonal	Cat#A19812
H3K36me3	Active Motif	Cat#61021
H4K12ac	Abcam	Cat#ab46983
H4K20me2	Diagenode	Cat#C15200205
HA	Proteintech	Cat#51064
MCM2	Cell signaling technology	Cat#3619
NPM1	Proteintech	Cat#10306-1-AP
POLD3	Proteintech	Cat#21935-1-AP
POLD3	Abscience	Cat#E-AB-61602
POLE3[1]	Bethyl	Cat#a301-245
POLE4[2]	Abclonal	Cat#A9882
WDHD1	Abcam	Cat#ab224221
SSRP1	Proteintech	Cat#15696-1-AP
BRD4	Proteintech	Cat#28486-1-AP
Mouse anti-IdU	BD Biosciences	Cat#347580
Rat anti-CldU	Abcam	Cat#ab6326
β-ACTIN	Beyotime	Cat#AF0003
Goat anti-Mouse IgG (H + L) HRP, Secondary Antibody	Beyotime	Cat#A0216
Goat anti-Rabbit IgG (H + L) HRP, Secondary Antibody	Beyotime	Cat#A0208
Rabbit-anti mice IgG H&L	Abcam	Cat#ab46540
Donkey anti-mouse Alexa Fluor 594	Life Technologies	Cat#A21203
Donkey anti-rat Alexa Fluor 488	Jackson ImmunoResearch	Cat#712-546-150

Supplementary Table 2: Commercial reagents and materials

Reagents and materials	SOURCE	IDENTIFIER
Glutathione Sepharose 4B	Cytiva	Cat#17075601
DNase I	Sigma-Aldrich	Cat#10104159001
Protein A/G magnetic beads	MedChemExpress	Cat#HY-K0202
Cell Counting Kit-8	MedChemExpress	Cat#HY-K0301

1. Li Z, Duan S, Hua X, Xu X, Li Y, Menolfi D*, et al.* Asymmetric distribution of parental H3K9me3 in S phase silences L1 elements. *Nature* 2023, **623**(7987)**:** 643-651.

2. Xu X, Duan S, Hua X, Li Z, He R, Zhang Z. Stable inheritance of H3.3-containing nucleosomes during mitotic cell divisions. *Nat Commun* 2022, **13**(1)**:** 2514.
