# Supplementary material for: Proximal proteomics analysis reveals DNA polymerase δ subunit 3 is a new MCM2 binding partner and promotes parental histones inheritance in mammalian cells
Source: Cell Death Differ. 2025 Nov 27;33(5):1050–64. doi: 10.1038/s41418-025-01619-z (PMC13156308; doi:10.1038/s41418-025-01619-z)
Supplement: Supplementary file 2 — Original Western blot of the Figures [file 41418_2025_1619_MOESM2_ESM.docx]

**Figure 2C**


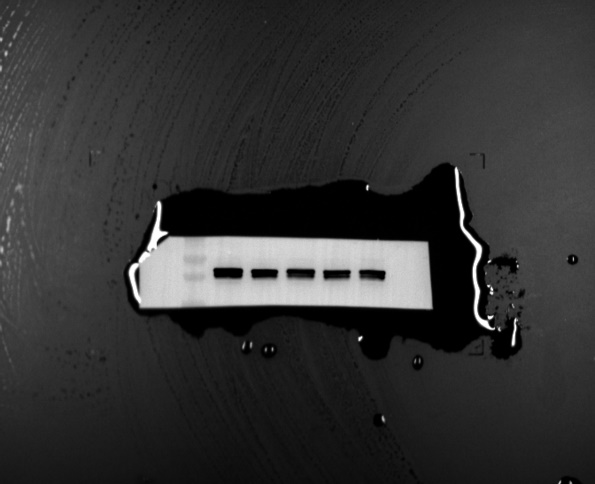

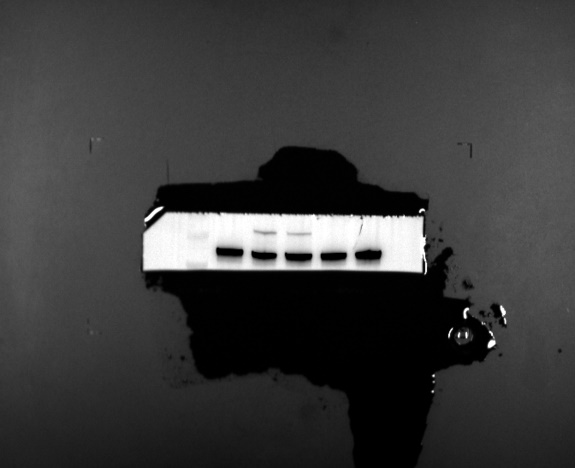


Empty Vector

MCM2-TurboID

MCM2-2A-TurboID

POLE3-TurboID

POLE4-TurboID

INPUT

MCM2

NPM1

KDa

130-

33-

180-

Empty Vector

MCM2-TurboID

MCM2-2A-TurboID

POLE3-TurboID

POLE4-TurboID


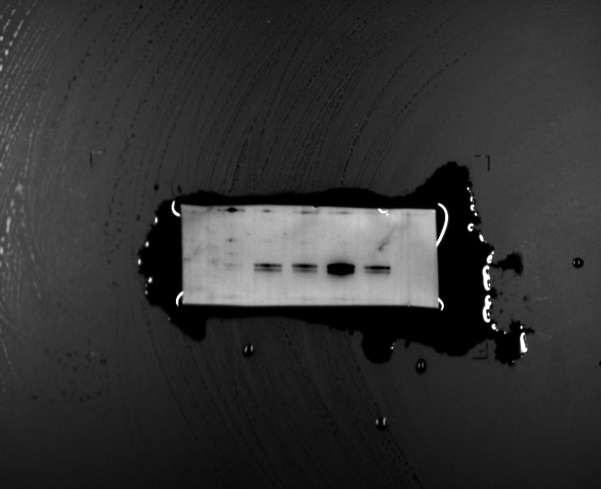

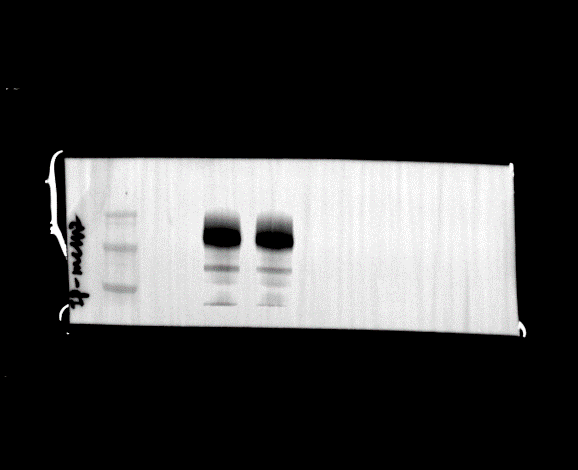


MCM2

NPM1

IP

130-

33-

180-

KDa

**Figure 3E**


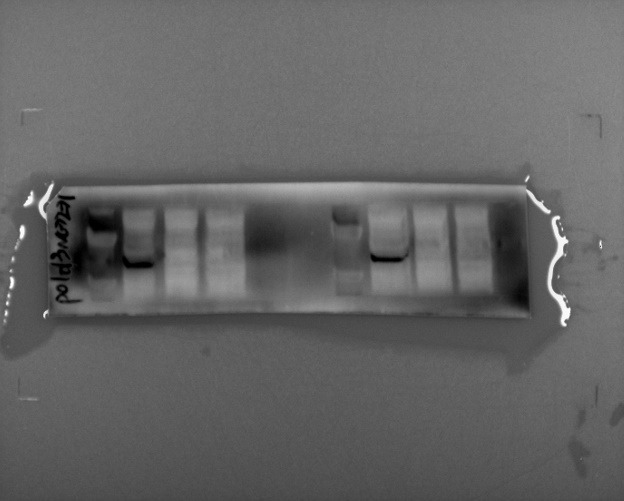


KDa

70-

KDa

POLD3

R1

R2

siRNA-NC

siRNA1-POLD3

siRNA2-POLD3

siRNA-NC

siRNA1-POLD3

siRNA2-POLD3


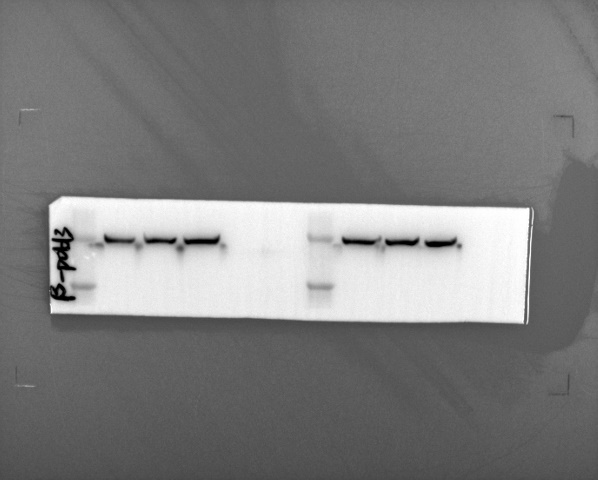


42-

β-ACTIN

R2

R1

siRNA-NC

siRNA1-POLD3

siRNA2-POLD3

siRNA-NC

siRNA1-POLD3

siRNA2-POLD3

KDa

KDa

**Figure 4A**


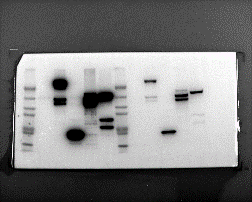

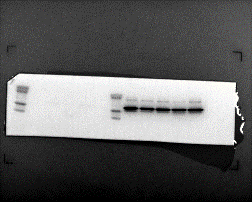


INPUT

HA

H3

70-

55-

17-

KDa

Empty Vector

POLD3-HA

POLD3(1-144)-NLS-HA

POLD3(145-466)-NLS-HA

19.9-

IP


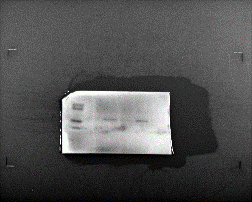


H3

17-


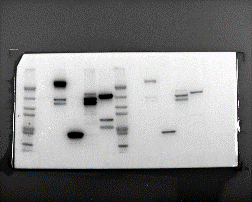


HA

KDa

70-

19.9-

55-

Empty Vector

POLD3-HA

POLD3(1-144)-NLS-HA

POLD3(145-466)-NLS-HA

**Figure 4B**


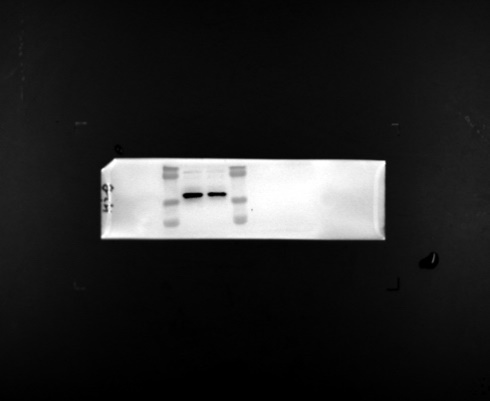


25-

15-

H2B


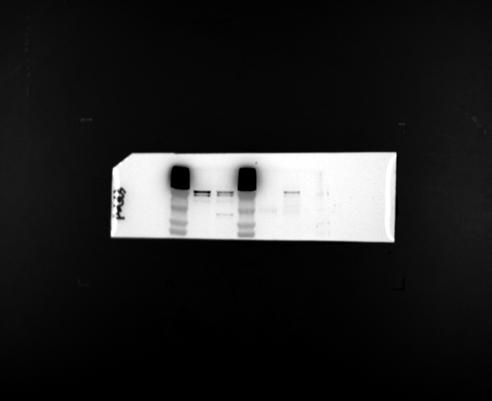


70-

50-

POLD3


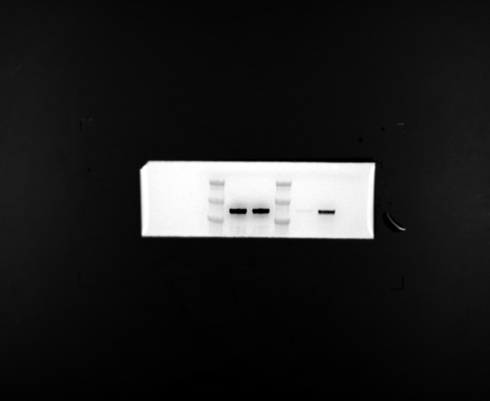


150-

100-

MCM2


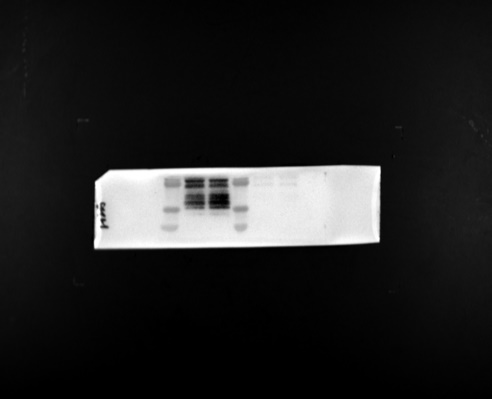


H2A

25-

15-


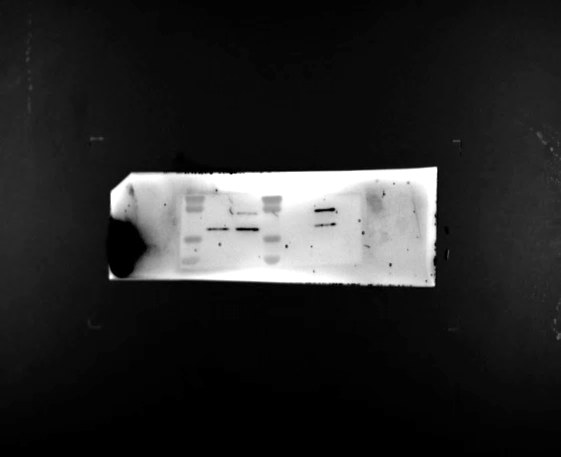


H3

25-

15-


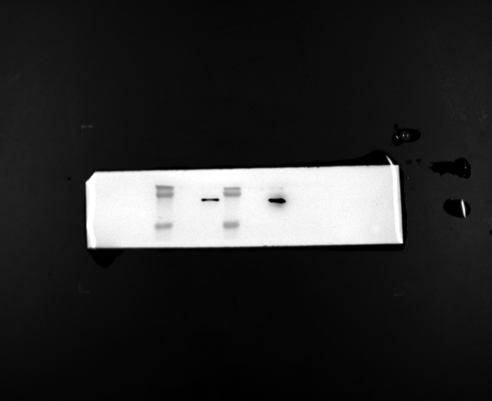


KDa

KDa

25-

15-

FLAG

INPUT

IP

Empty Vector

Flag-H3.1

Empty Vector

Flag-H3.1

**Figure 5H**

KDa

KDa

INPUT

IP


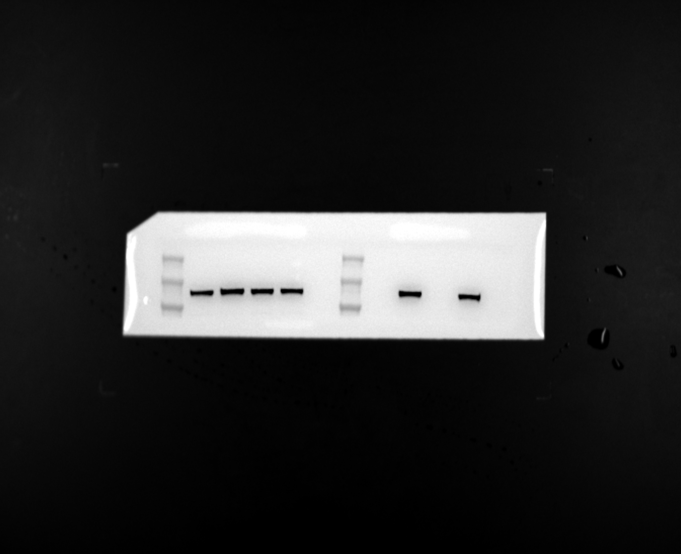


250-

150-

100-

MCM2


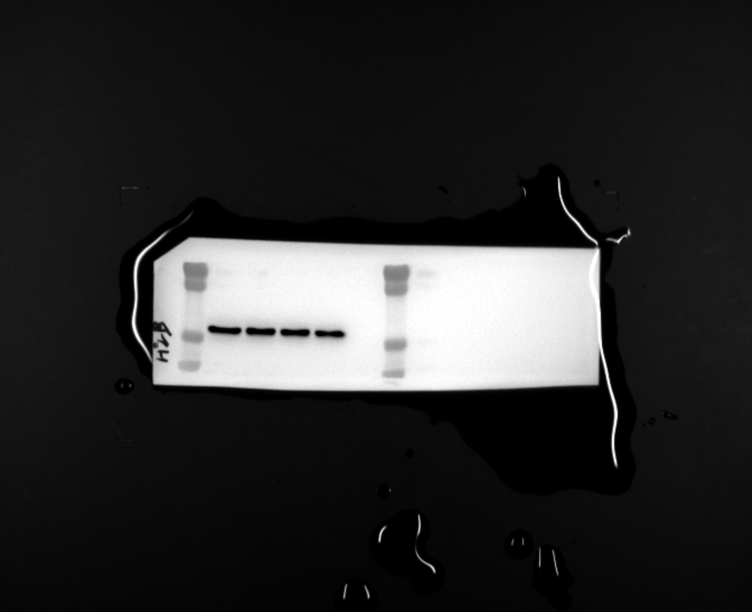


H2B

15-


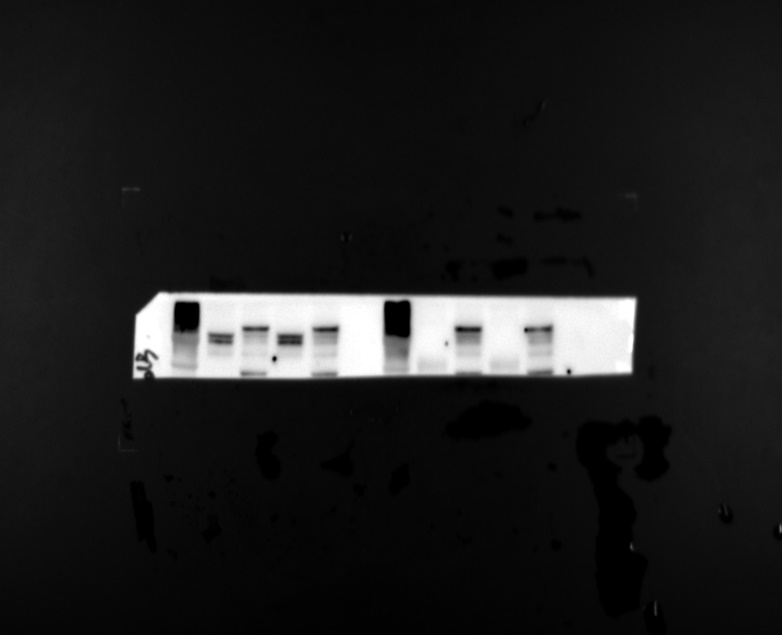


POLD3

70-

15-


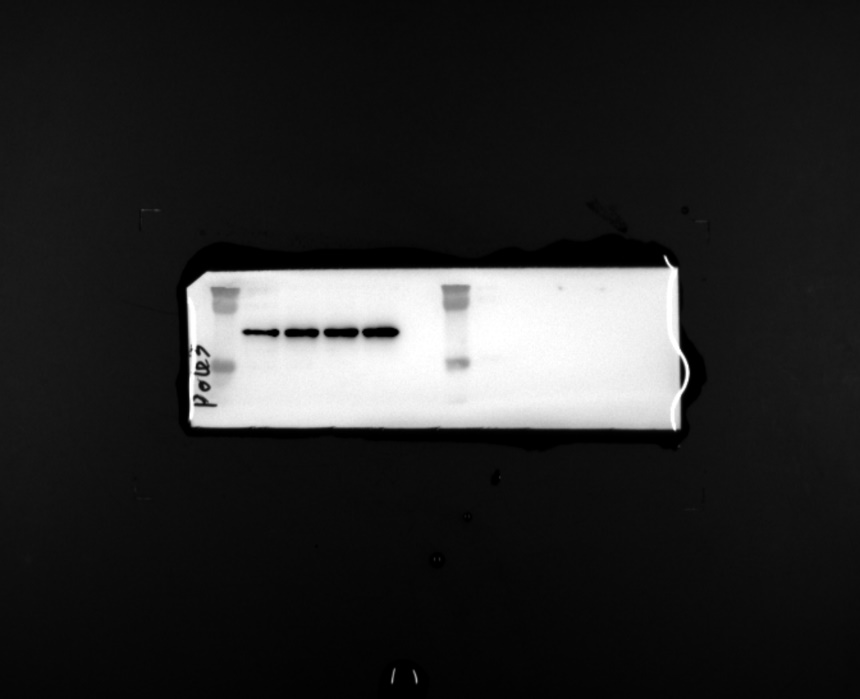


POLE3

20-


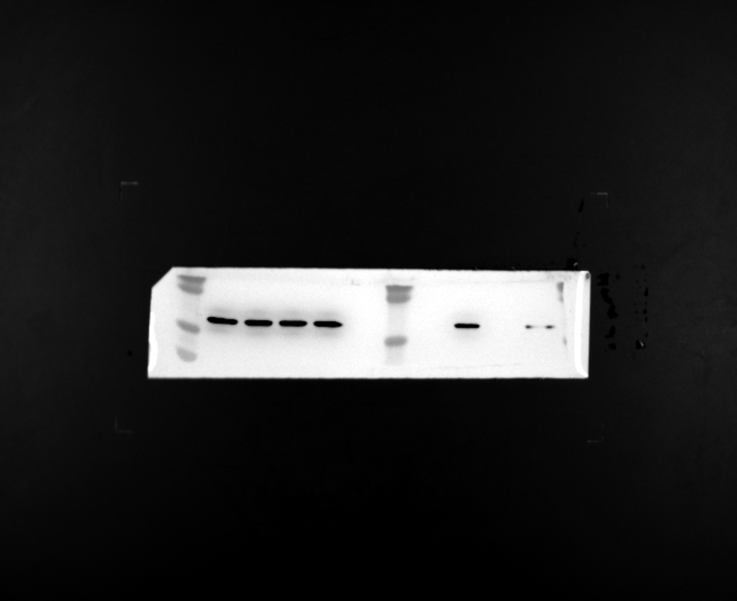


H3

20-

15-


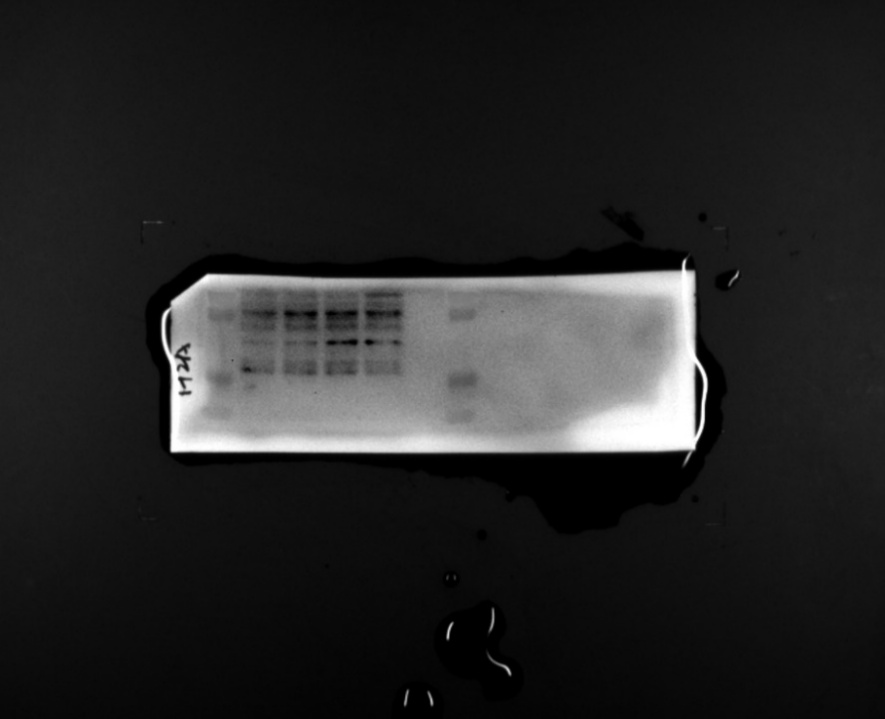


H2A

20-

15-

Empty Vector

POLD3-HA

Empty Vector

POLD3-HA

**MCM2-WT MCM2-2A**

Empty Vector

POLD3-HA

Empty Vector

POLD3-HA

**MCM2-WT MCM2-2A**


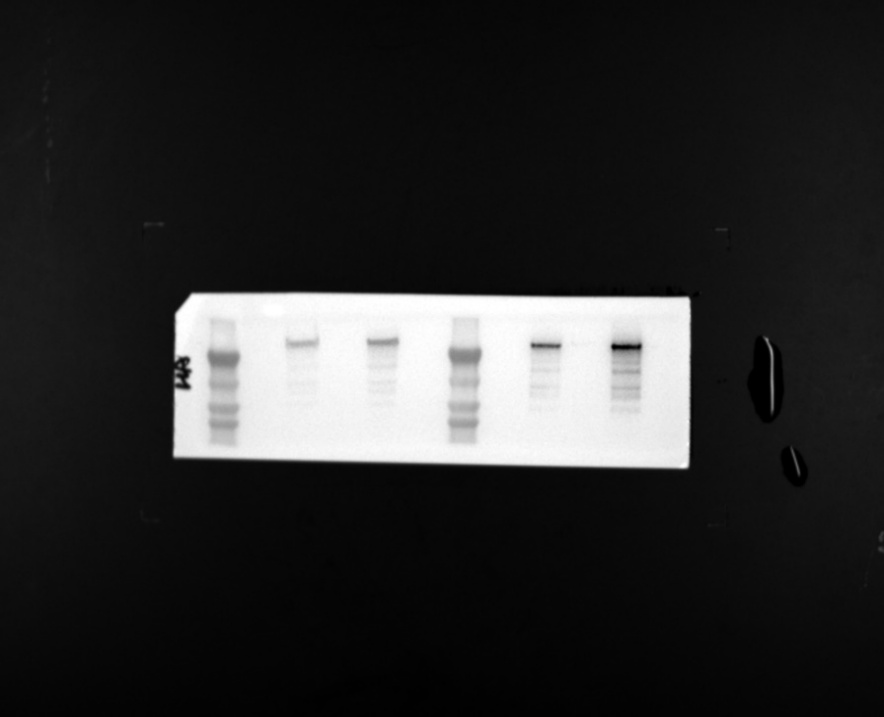


HA

70-

**Supplementary Figure 3**

IP

POLE3-TurboID

POLE4-TurboID

Empty Vector

MCM2-TurboID

MCM2-2A-TurboID


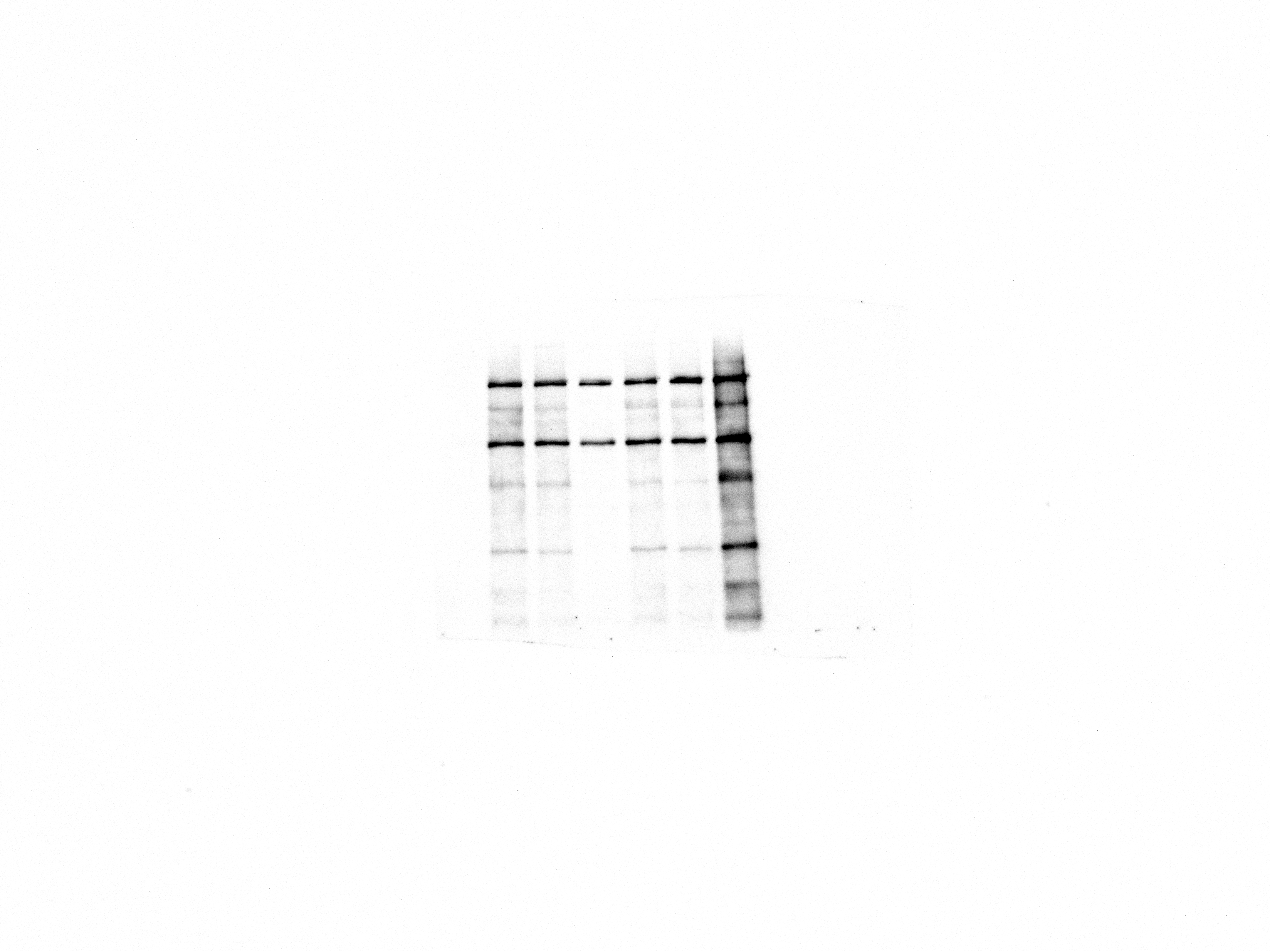


**Supplementary Figure 6**

KDa


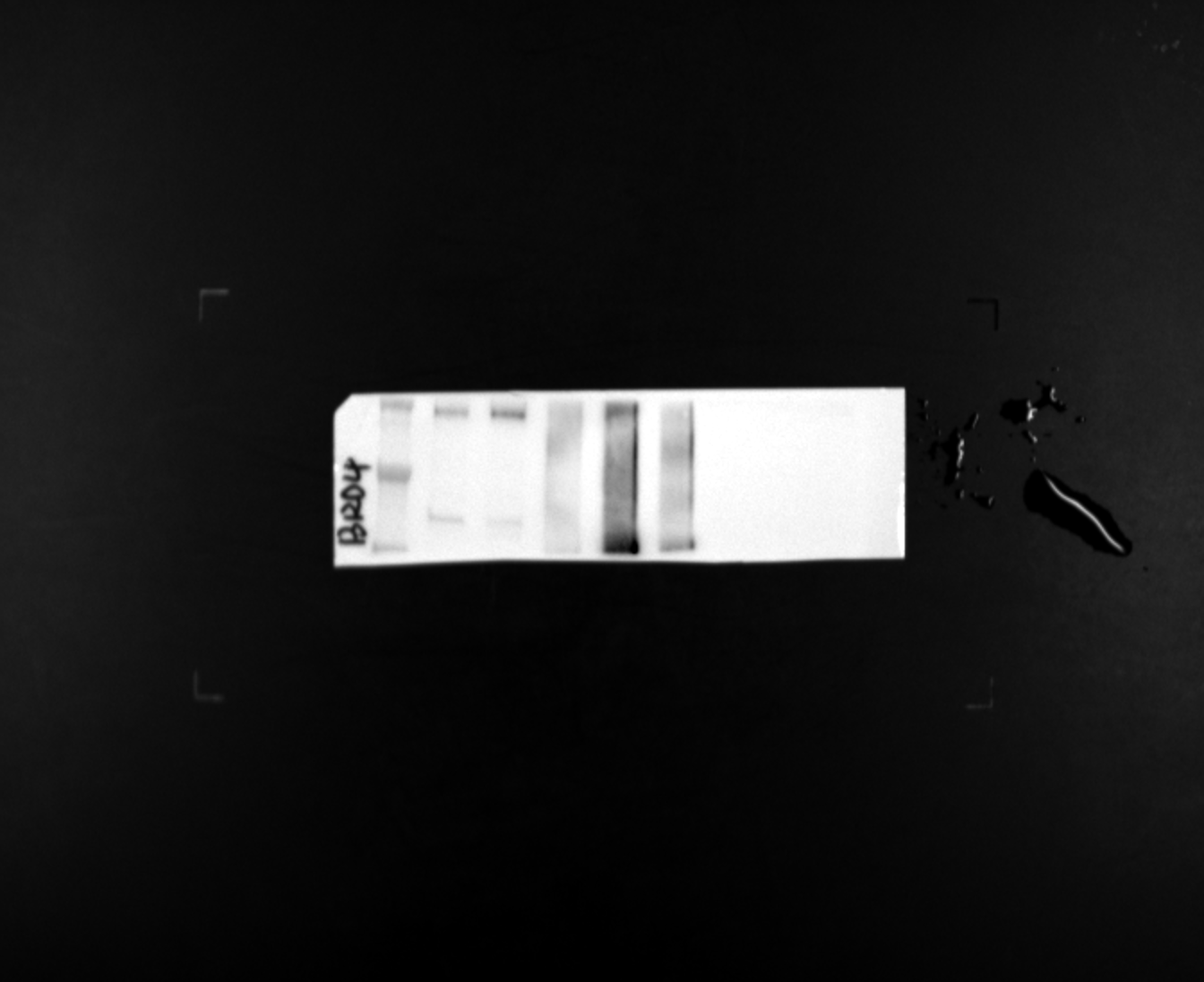


BRD4

Input

IgG IP MCM2-IP

293T(MCM2-WT)

293T(MCM2-2A)

293T(MCM2-WT)

293T(MCM2-WT)

293T(MCM2-2A)

MCM2


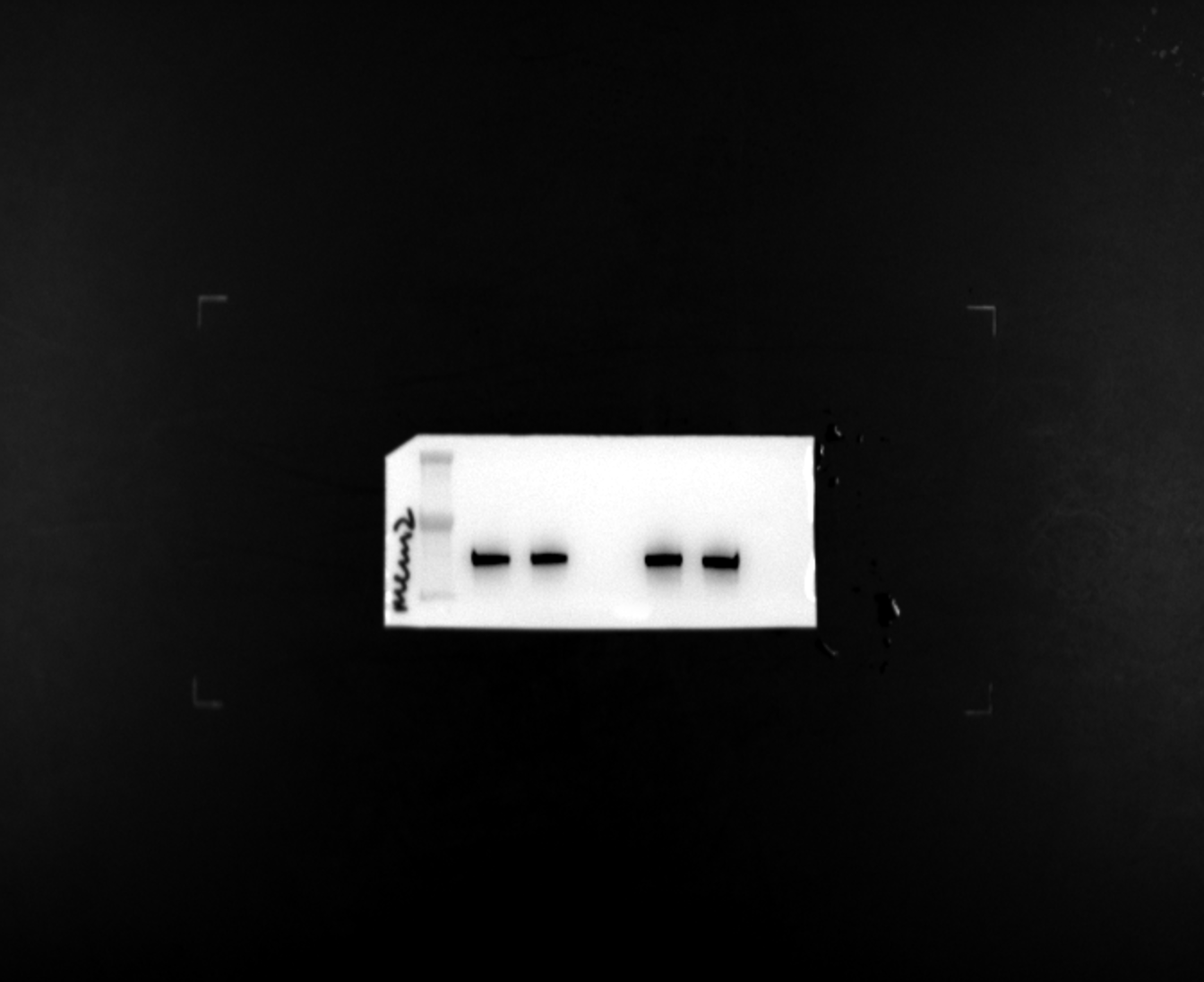


130-


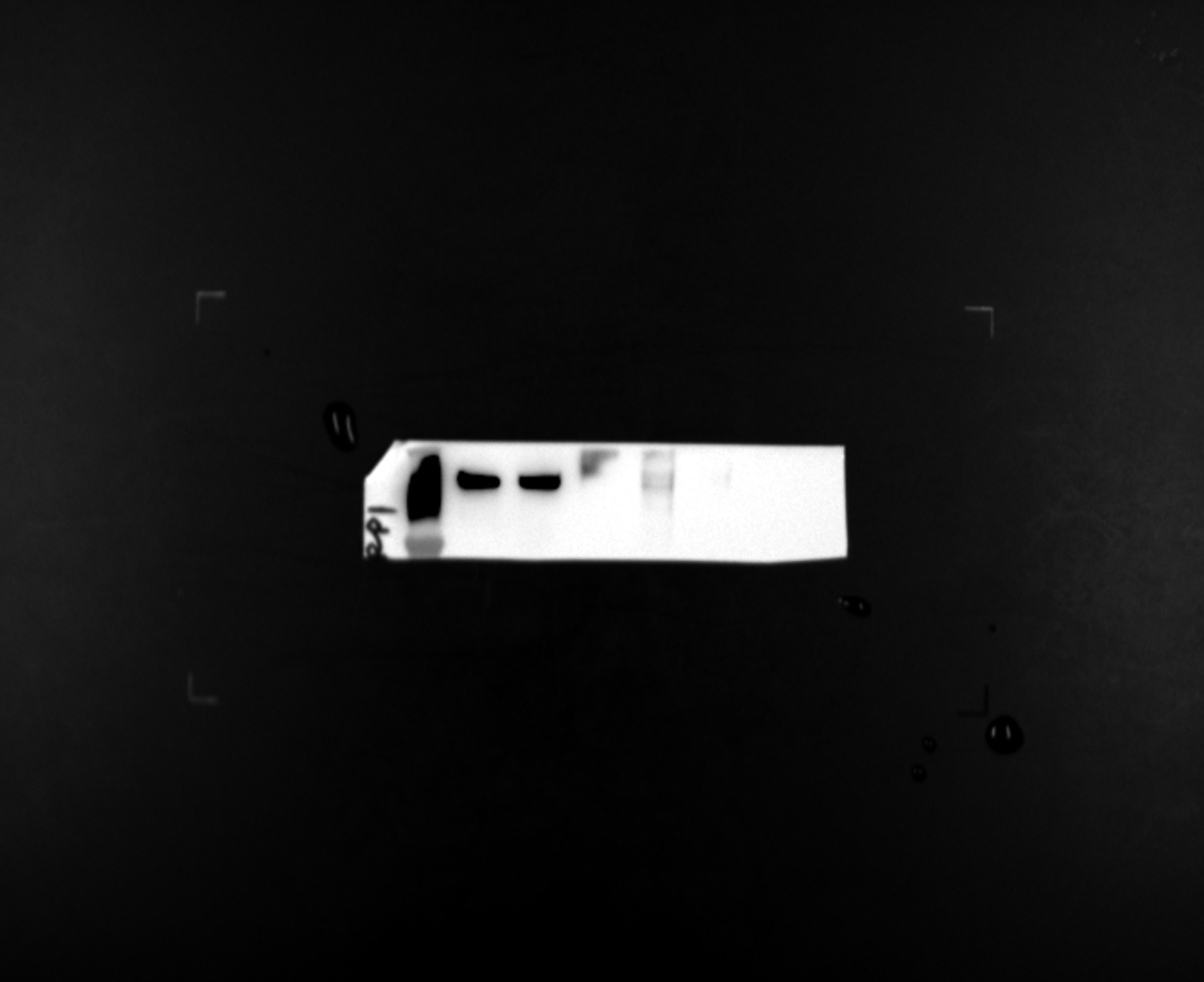


SSRP1

-80

250-

250-

**Supplementary Figure 8A**


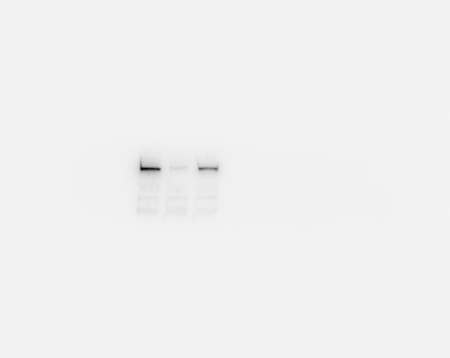

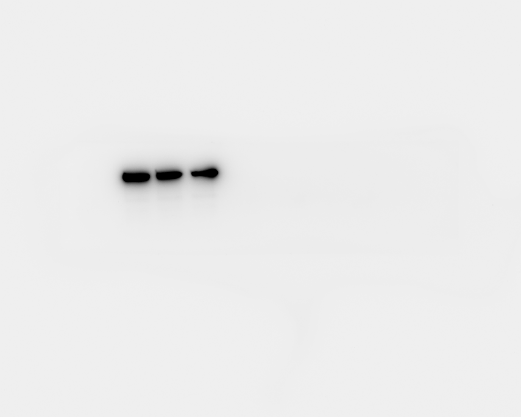


WDHD1

β-ACTIN

siRNA-NC

siRNA1-WDHD1

siRNA2-WDHD1

KDa

140-

42-

**Supplementary Figure 10B**

KDa

H3

POLD3


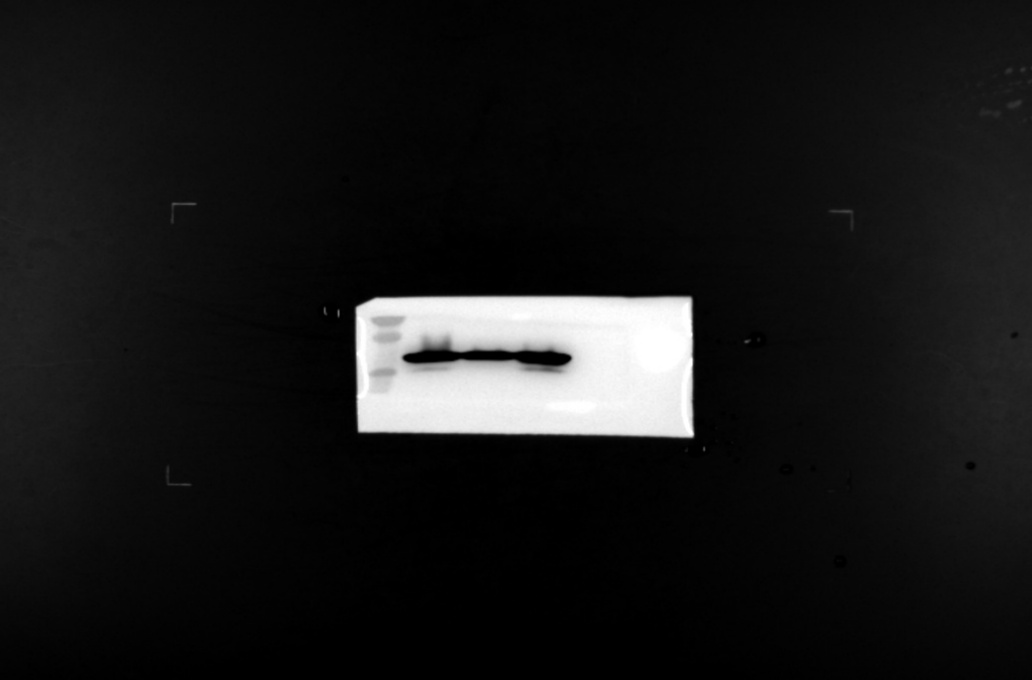


17-


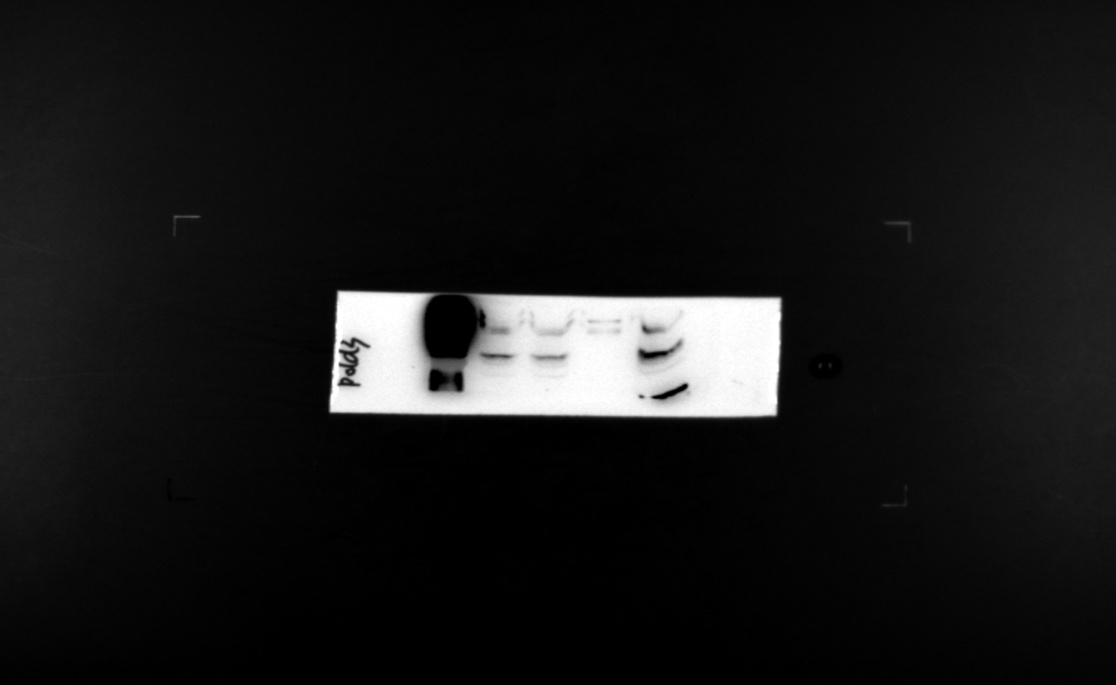


siRNA-Ctrl

siRNA1-POLD3

siRNA2-POLD3

-70

**Supplementary Figure 11A**


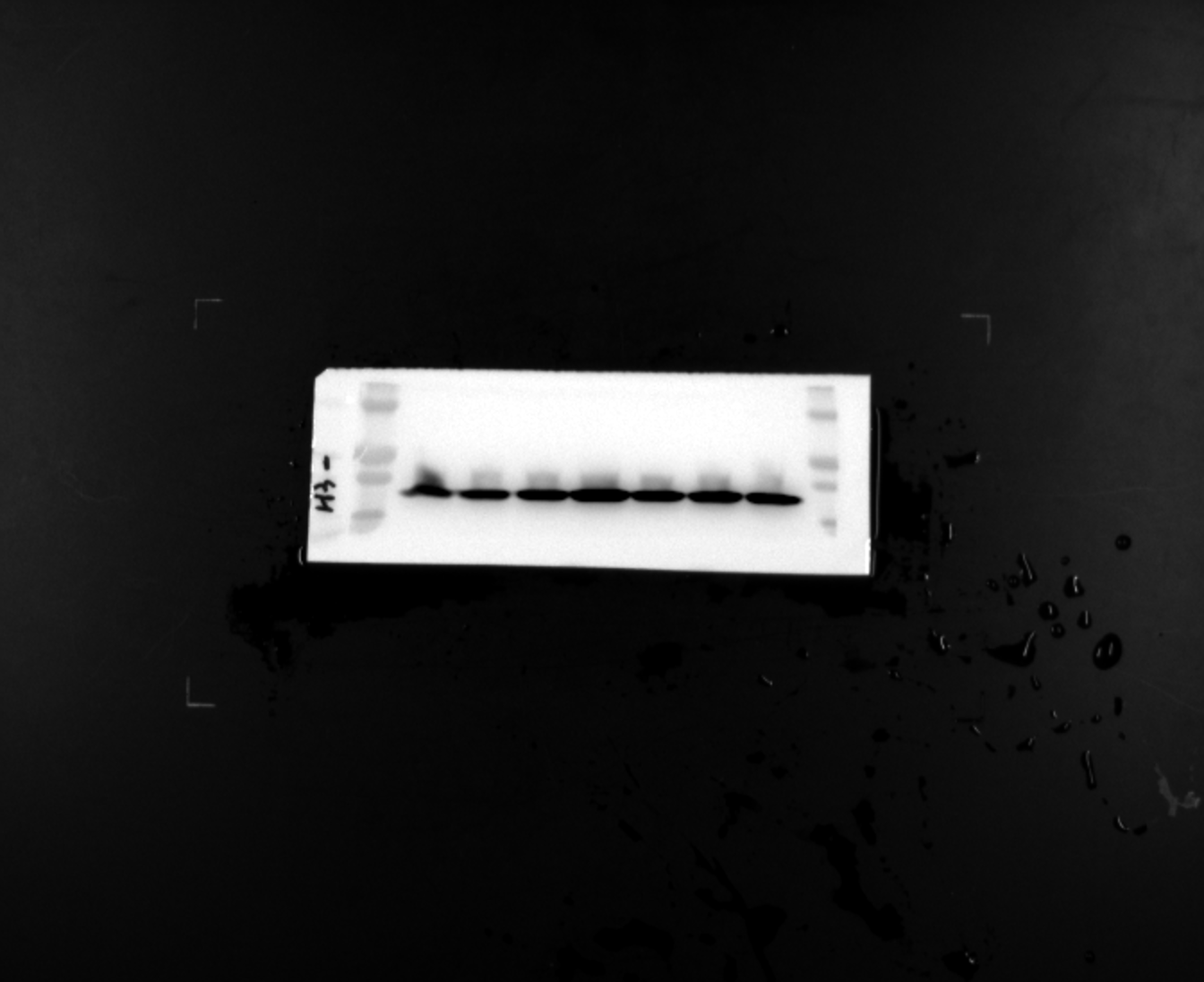


H3

17-


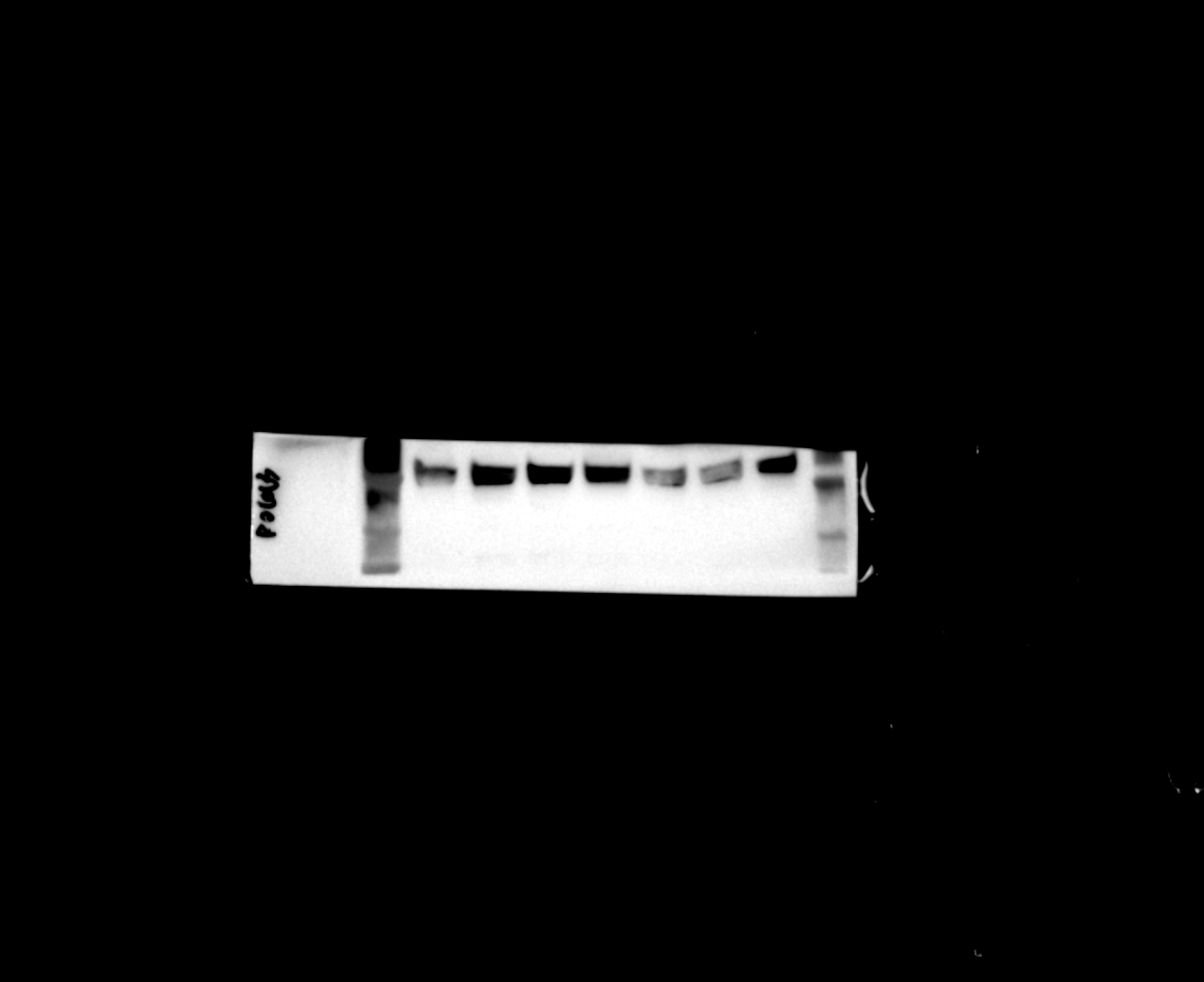


POLD3

70-

KDa

Empty Vector

POLD3(1-144)-NLS-HA

POLD3(145-466)-NLS-HA

POLD3-HA

POLD3(1-144)-NLS-HA

POLD3(145-466)-NLS-HA

POLD3-HA

Dox-

Dox+

**Supplementary Figure 11B**


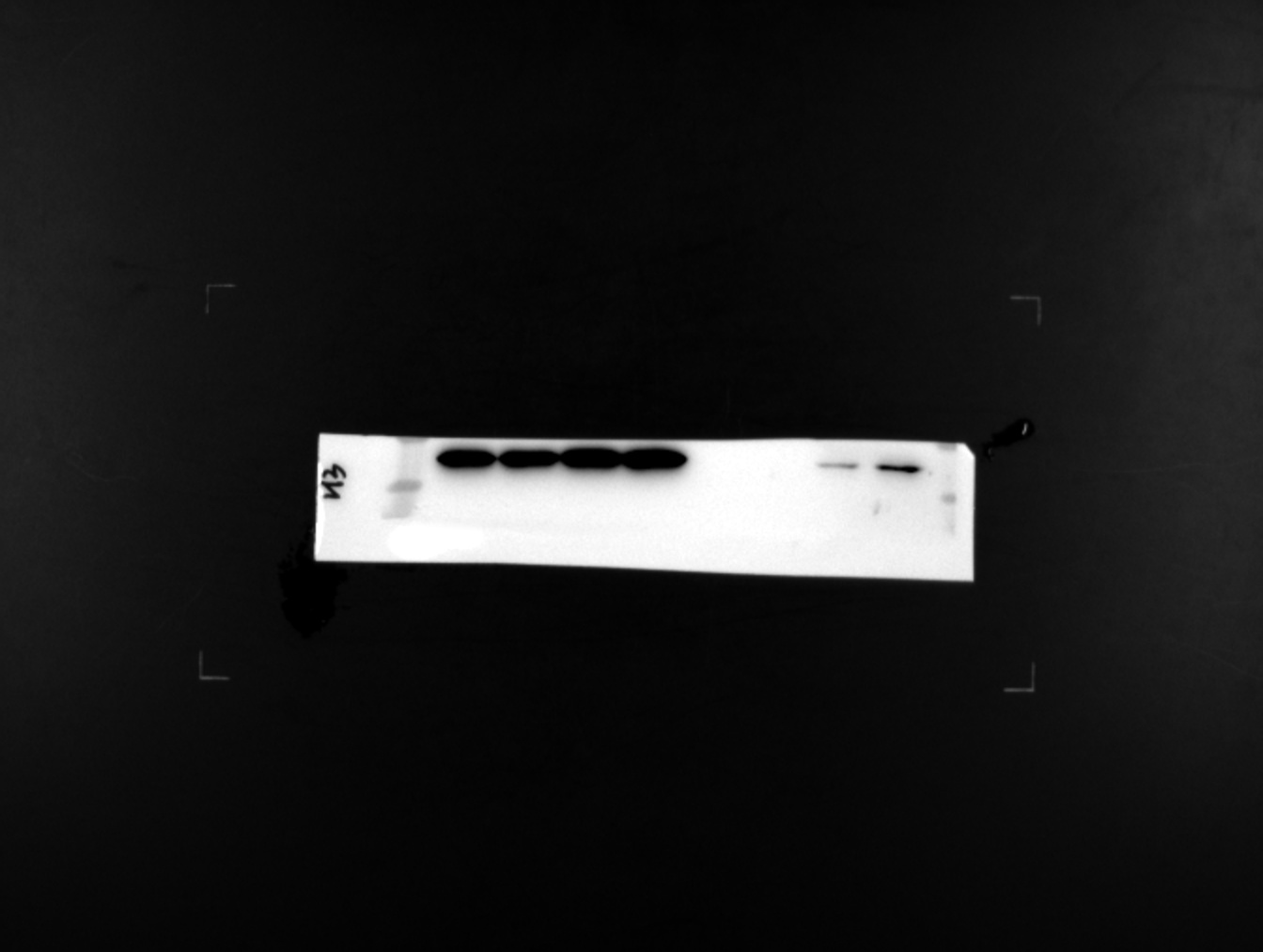

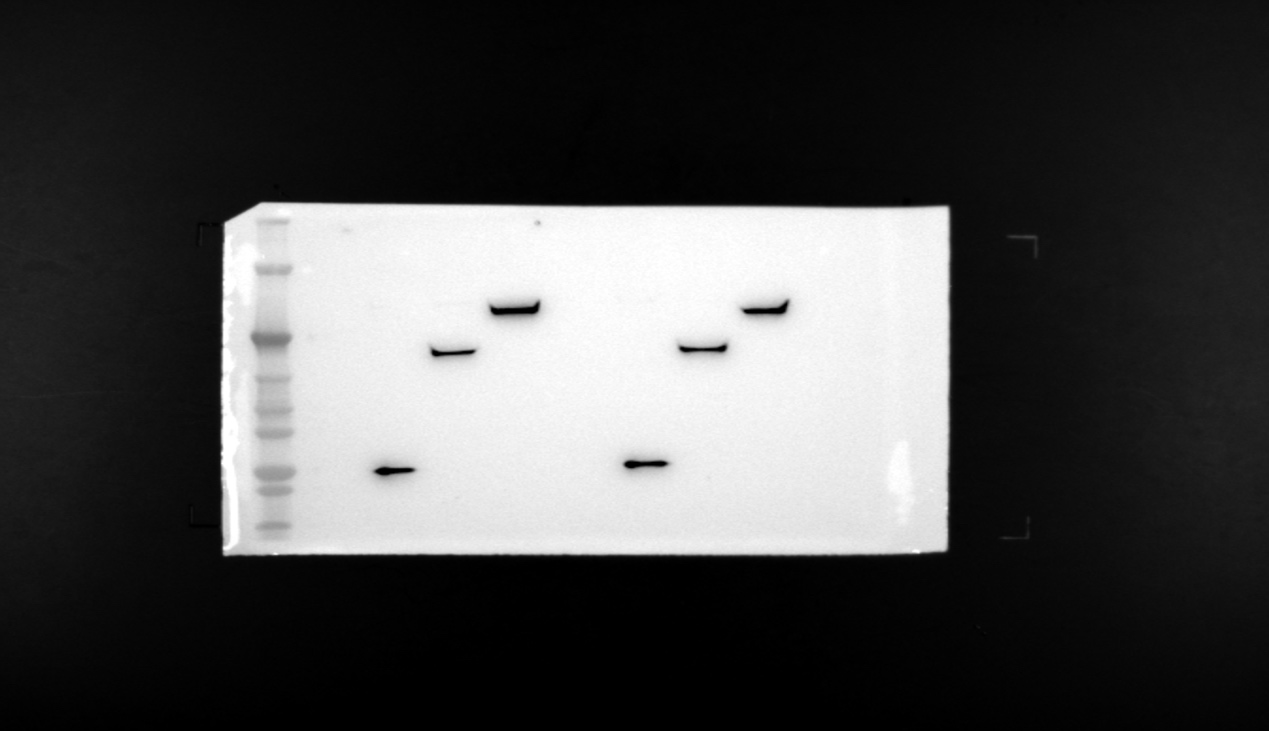


70-

19.9-

55-

H3

HA

17-

KDa

Empty Vector

POLD3(1-144)-NLS-HA

POLD3(145-466)-NLS-HA

POLD3-HA

Empty Vector

POLD3(1-144)-NLS-HA

POLD3(145-466)-NLS-HA

POLD3-HA

INPUT

IP

Dox+

**Supplementary Figure 14**


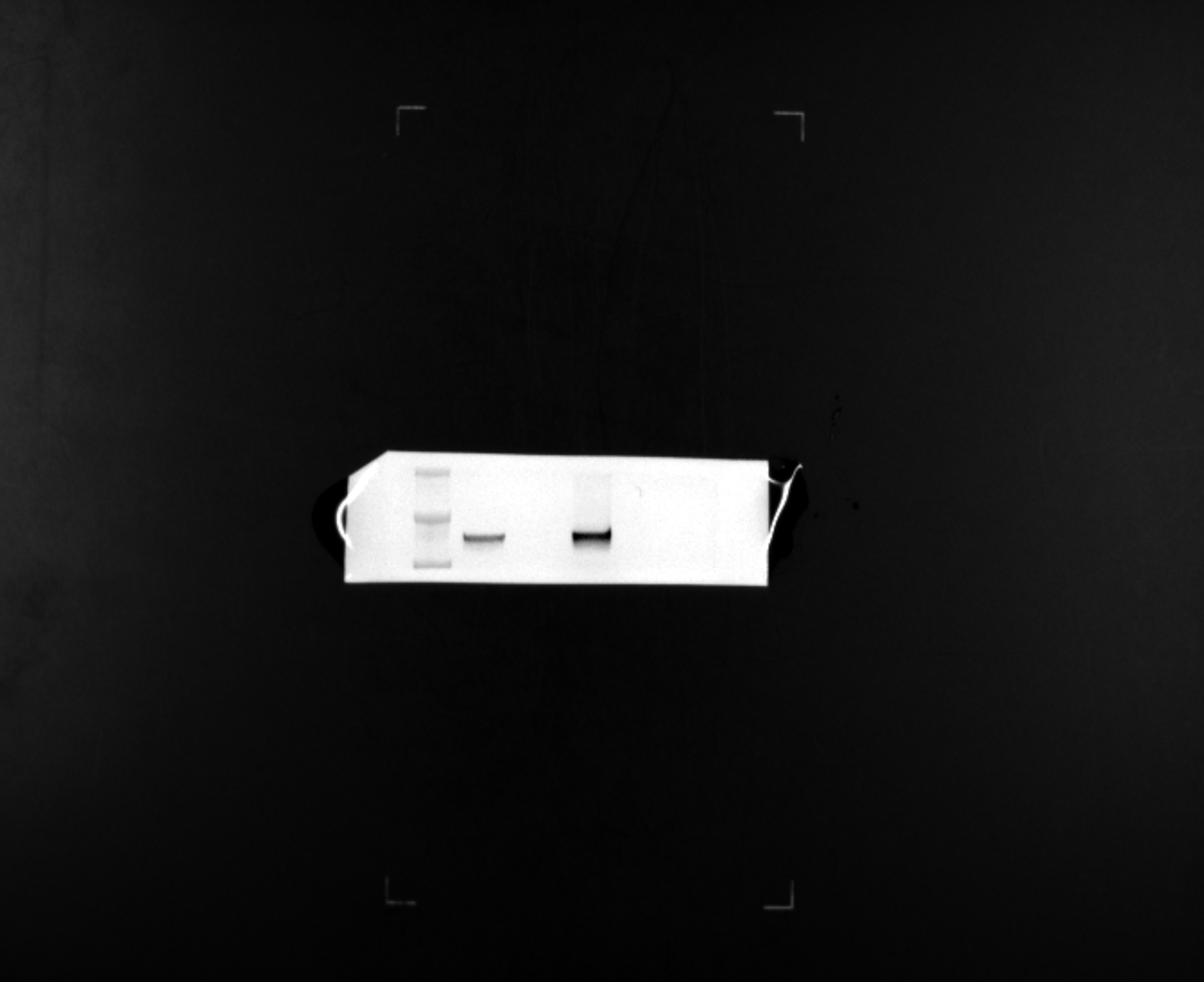

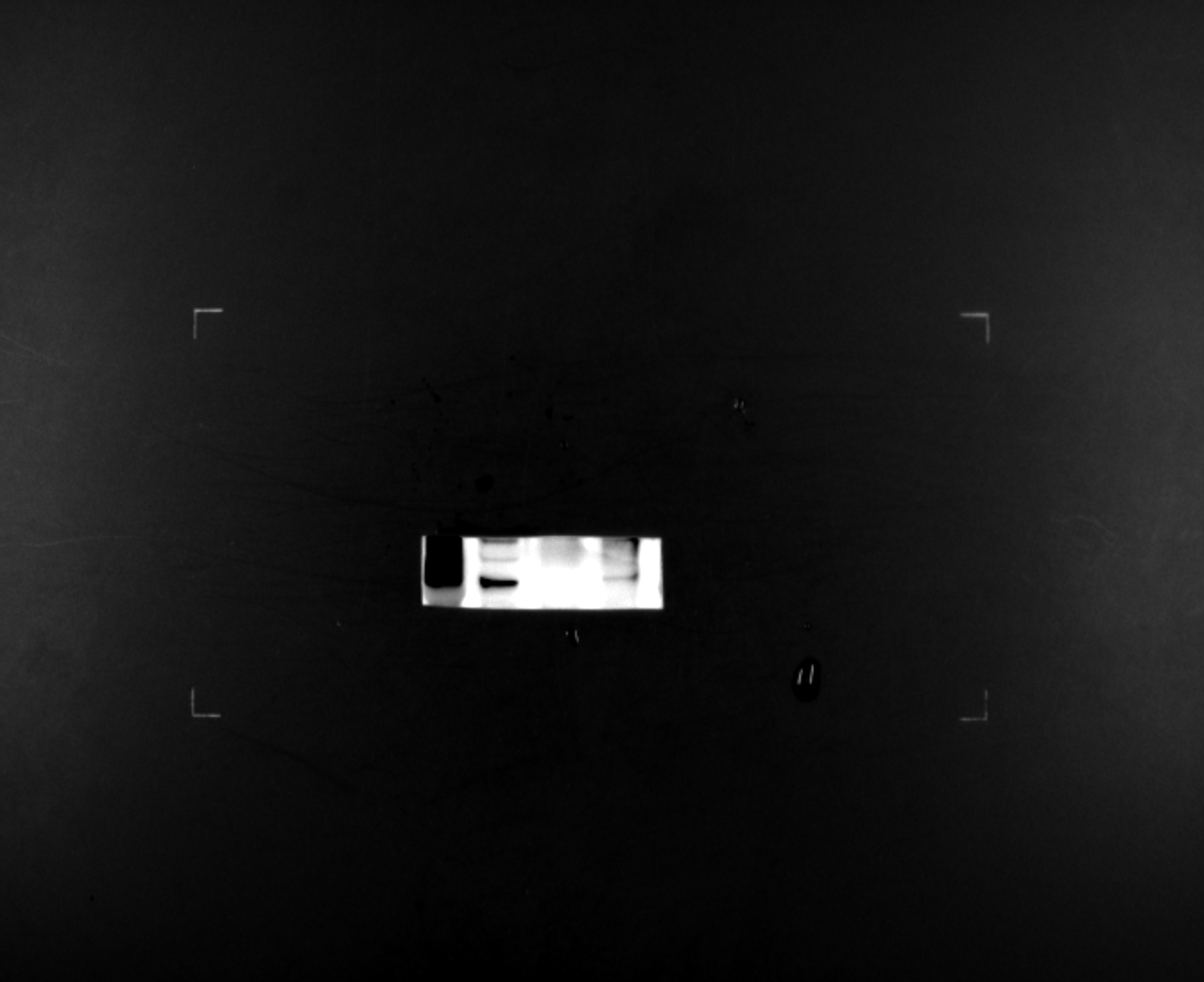

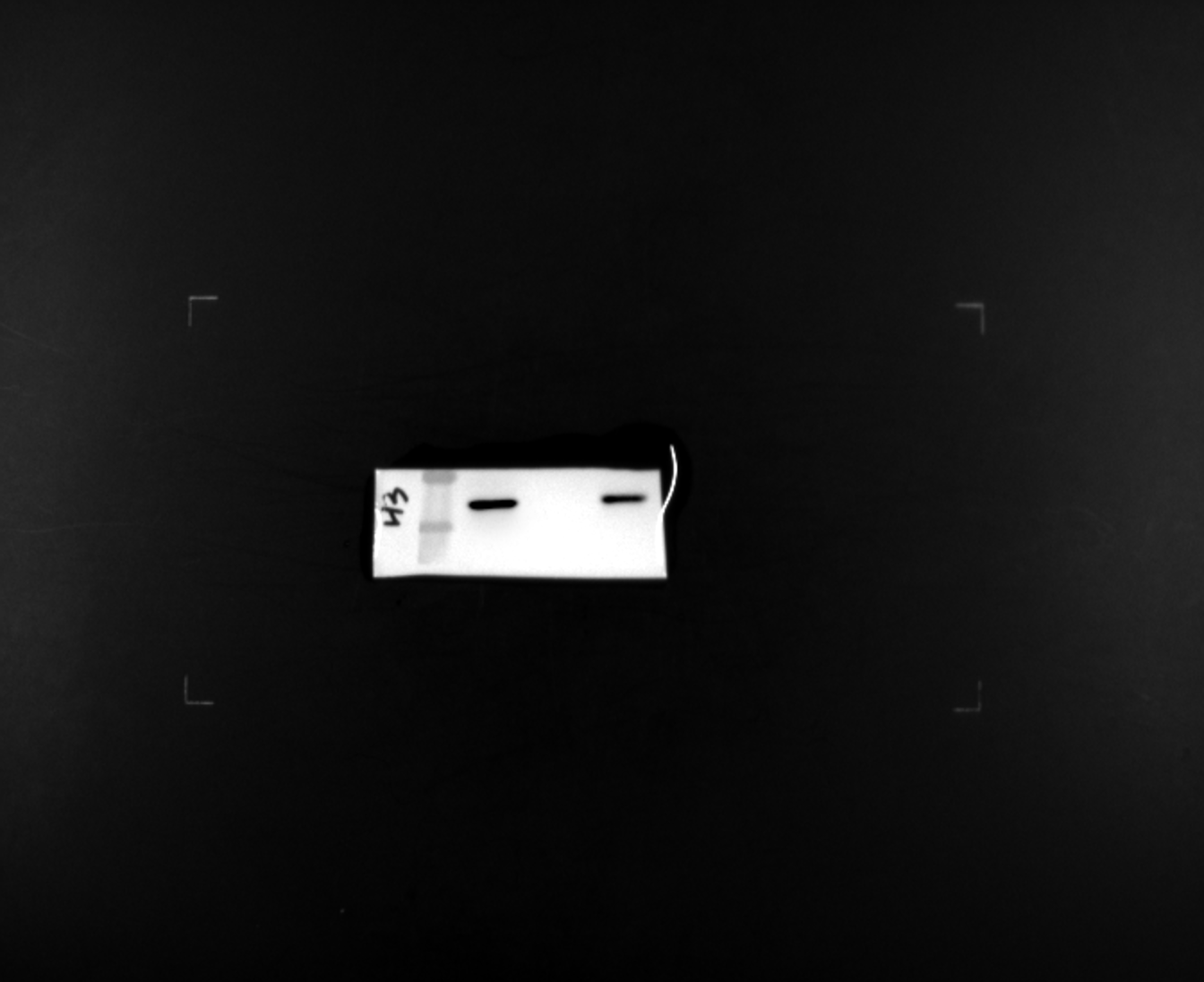


MCM2

POLD3

H3

17-

130-

70-

KDa

INPUT

IgG

MCM2

IP

**Supplementary Figure 15A**

KDa

WT

MCM2-TurboID

MCM2-2A-TurboID


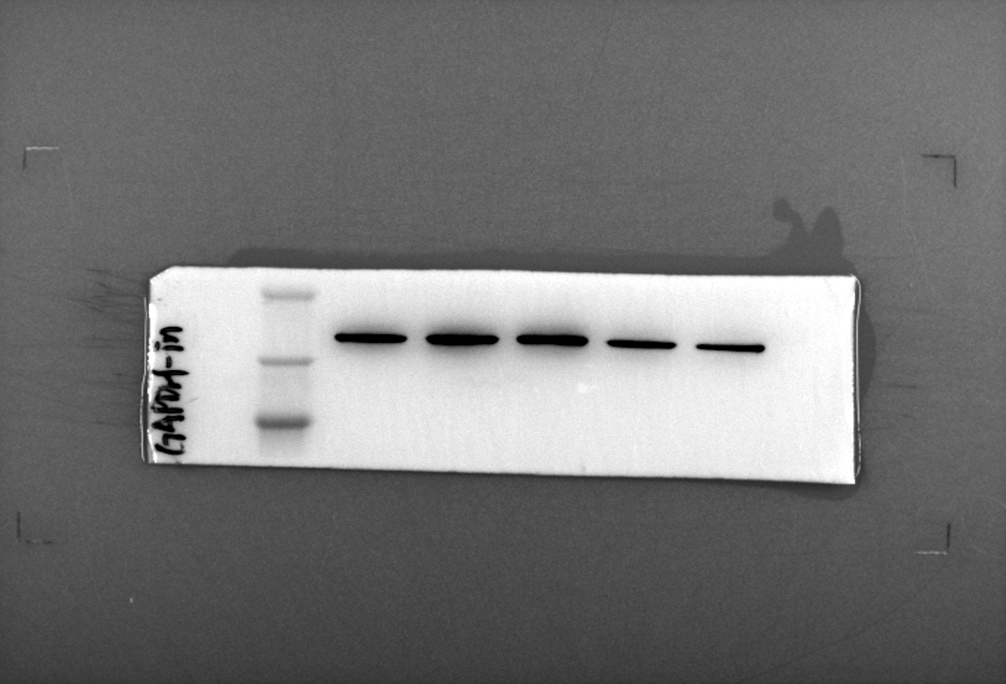


GAPDH

MCM2

37-


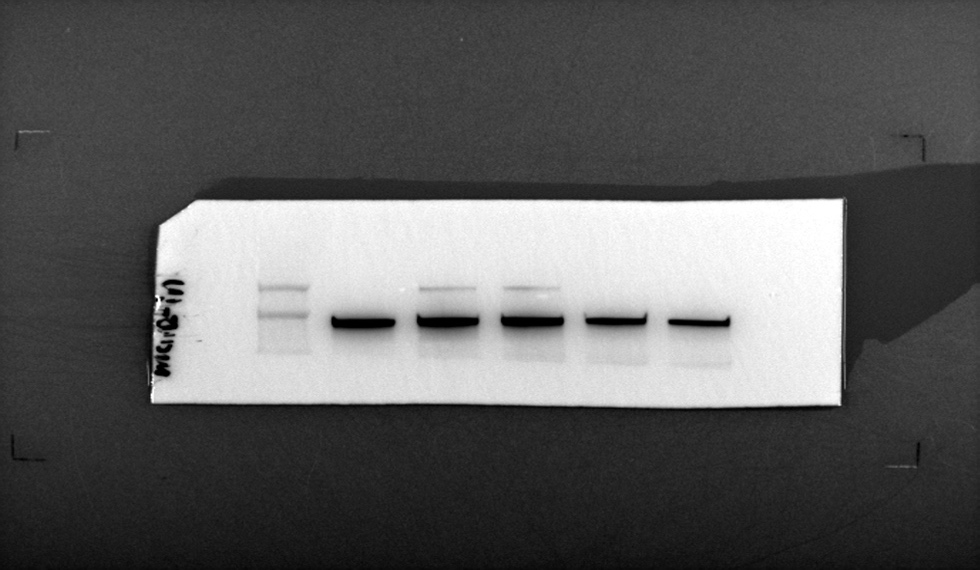


130-

180-

**Supplementary Figure 15B**

POLE3


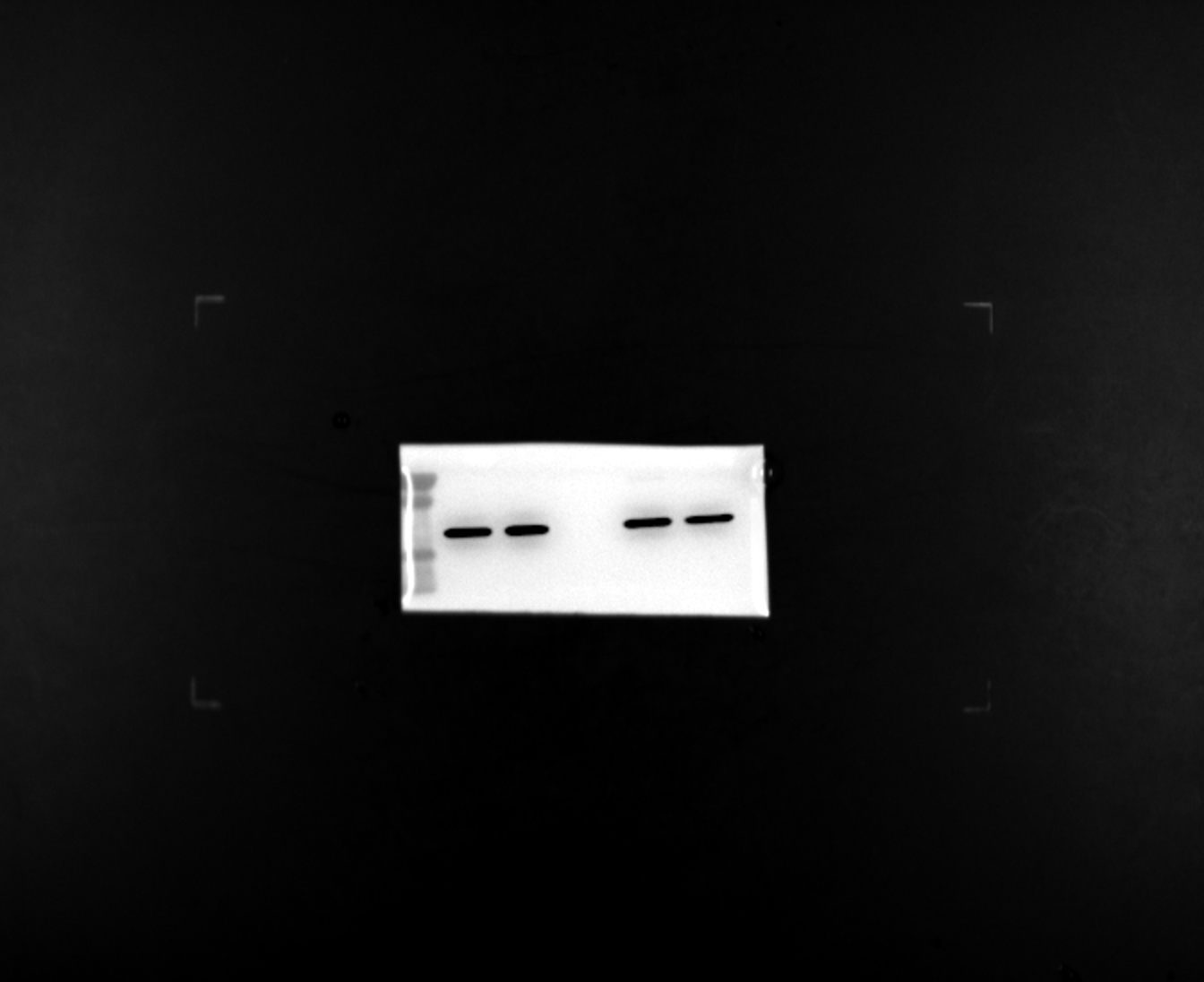


17-

H3


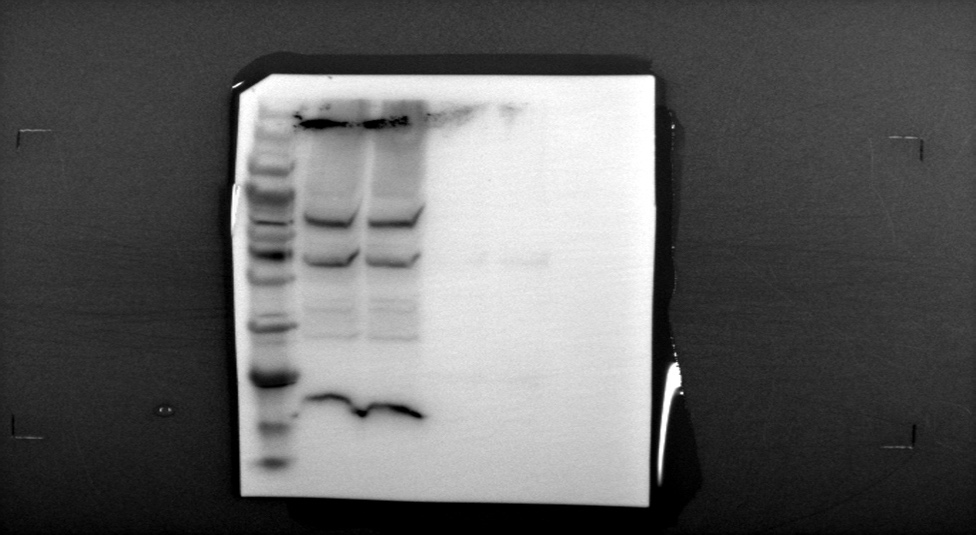


KDa

17-

60-

WT

POLE3-TurboID

**Supplementary Figure 15C**

POLE4

H3


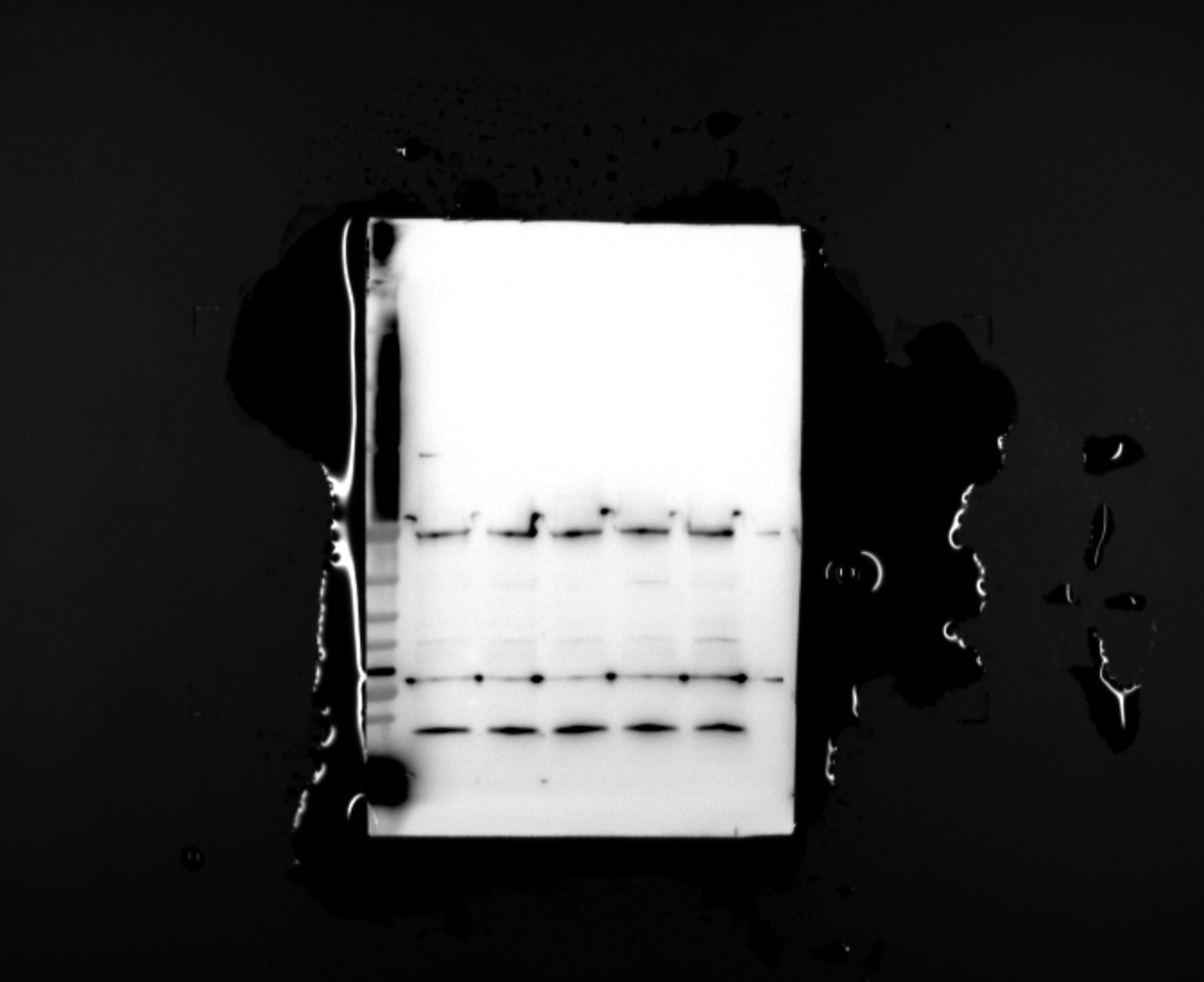


KDa

17-

70-


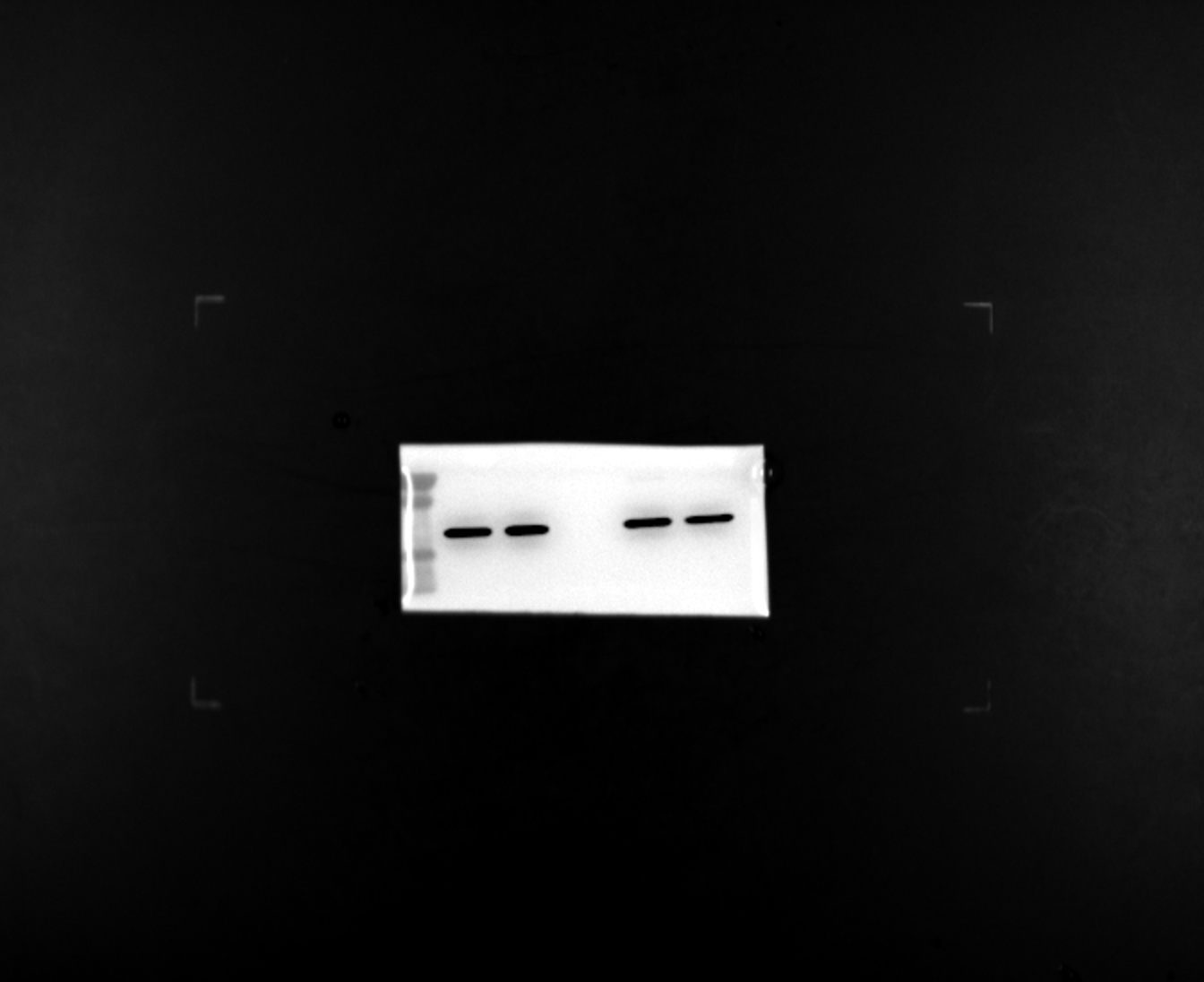


17-

WT

POLE4-TurboID

**Supplementary Figure 15D**


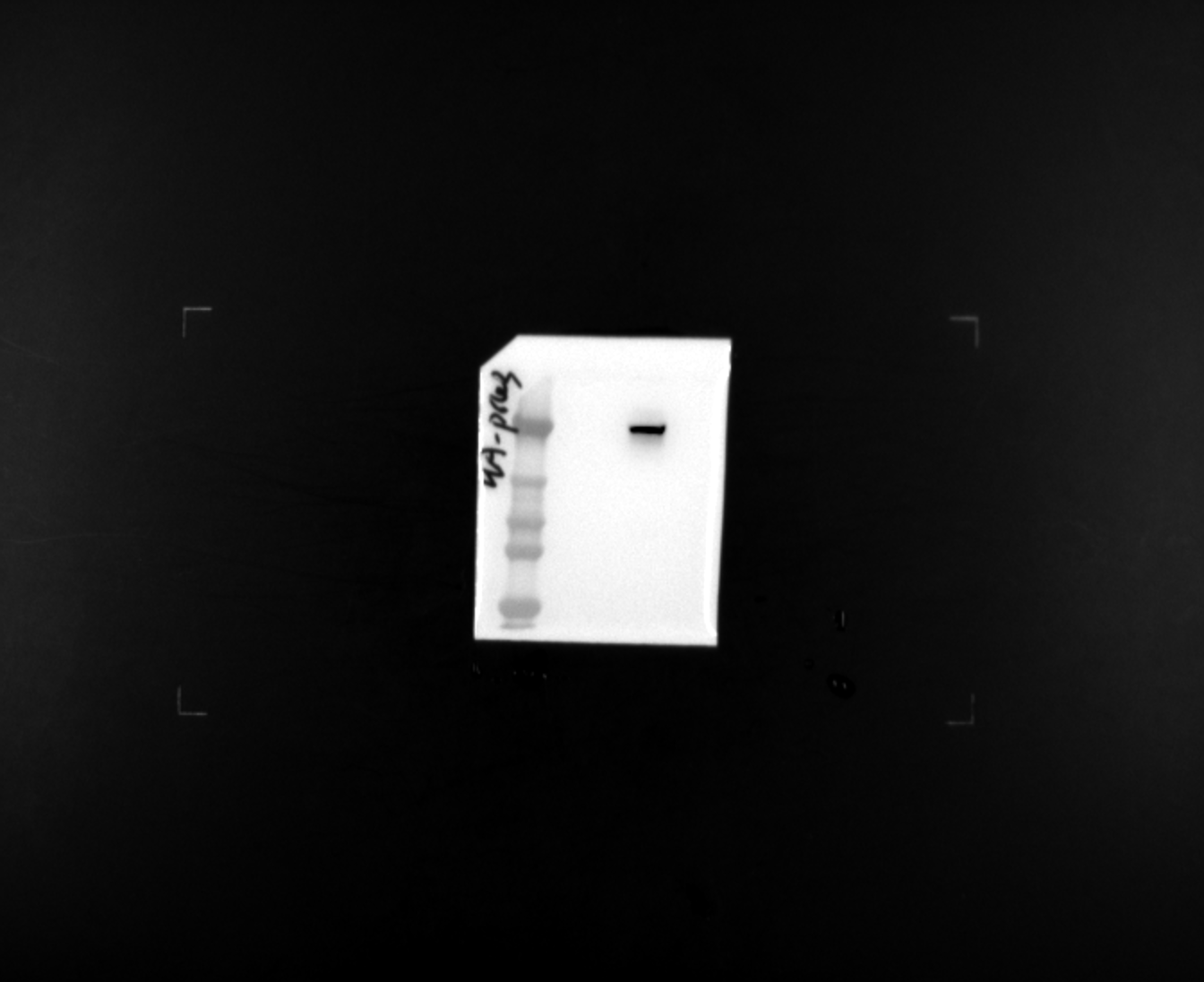


70-

KDa

HA

WT

POLE3-TurboID

**Supplementary Figure 15E**


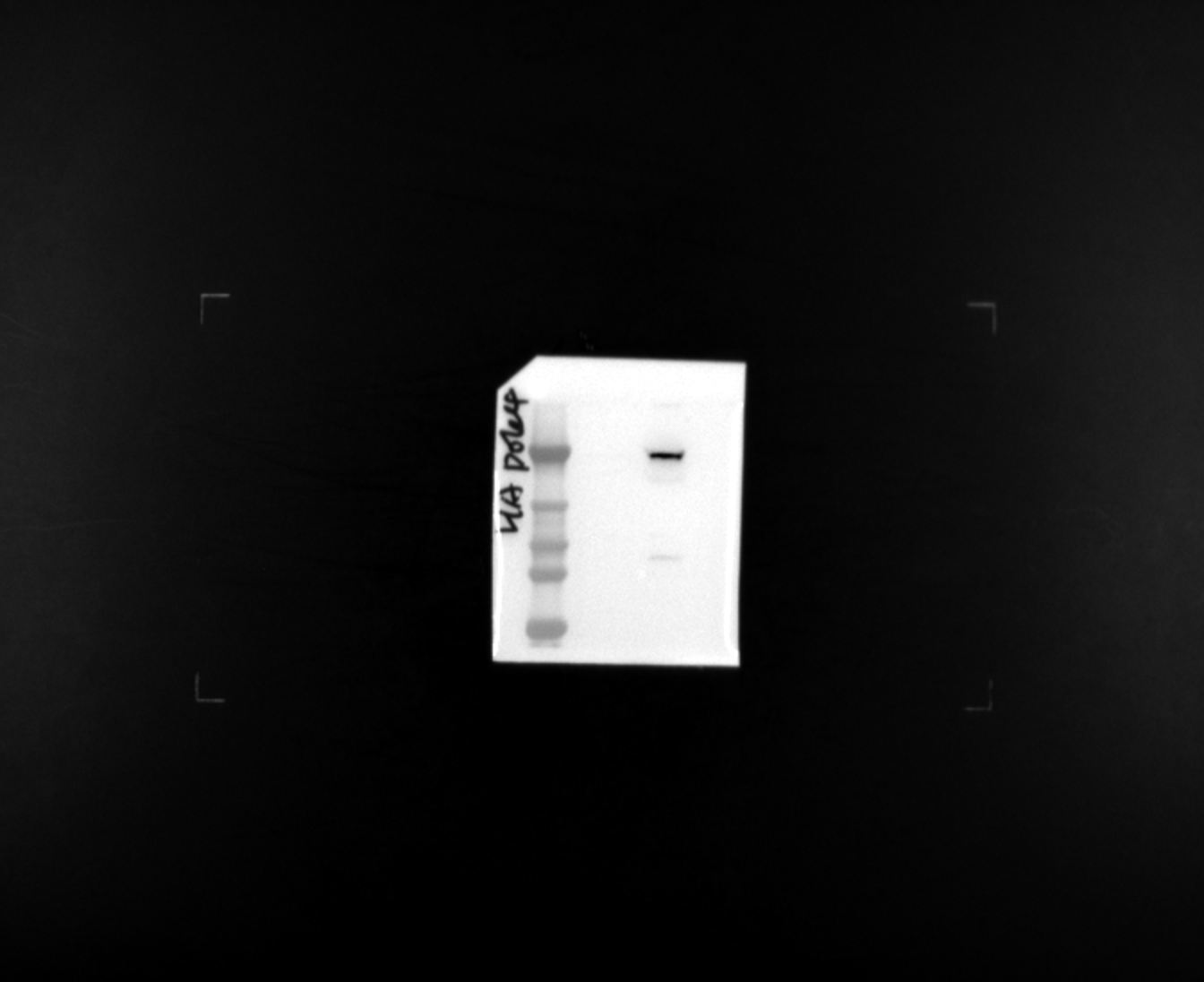


KDa

HA

70-

WT

POLE4-TurboID

20-

15-
